# Efficacy and Safety of Systemic Treatments Among Colorectal Cancer Patients: A Network Meta-Analysis of Randomized Controlled Trials

**DOI:** 10.3389/fonc.2021.756214

**Published:** 2022-02-09

**Authors:** Tung Hoang, Dae Kyung Sohn, Byung Chang Kim, Yongjun Cha, Jeongseon Kim

**Affiliations:** ^1^ Department of Cancer Biomedical Science, National Cancer Center Graduate School of Cancer Science and Policy, Goyang, South Korea; ^2^ Center for Colorectal Cancer, Research Institute and Hospital, National Cancer Center, Goyang, South Korea

**Keywords:** network meta-analysis, colorectal cancer, metastasis, chemotherapy, targeted therapy

## Abstract

**Background:**

Systemic treatments, namely, either monotherapy or combination therapy, are commonly administered to patients with advanced or metastatic colorectal cancer (CRC). This study aimed to provide the complete efficacy and safety profiles and ranking of systemic therapies for the treatment of unresectable advanced or metastatic CRC.

**Methods:**

We searched PubMed, Embase, the Cochrane Library, and ClinicalTrials.gov from inception until June 30, 2021, and also the bibliographies of relevant studies. Randomized controlled trials comparing two or more treatments, namely, at least capecitabine, 5-fluorouracil, leucovorin, irinotecan, bevacizumab, cetuximab, oxaliplatin, or panitumumab were investigated. A network meta-analysis using the Bayesian approach was performed to compare the efficacy and safety of treatments. The surface under the cumulative ranking curve (SUCRA) was calculated for the probability of each treatment as the most effective. The overall response rate (ORR), disease control rate (DCR), overall survival (OS), progression-free survival (PFS), adverse events (AEs) grade ≥3, and serious adverse events (SAEs) were evaluated.

**Results:**

One hundred two publications with 36,147 participants were assigned to 39 different treatments. Among 11 treatments with full information on six outcomes, FOLFIRI/FOLFOX/FOLFOXIRI + bevacizumab significantly improved both the ORR and DCR, compared to FOLFIRI. Although FOLFOX and FOLFIRI/FOLFOX + cetuximab significantly prolonged both OS and PFS, treatments were comparable in terms of AEs grade ≥3 and SAEs. The top highest SUCRA values were observed in the FOLFOXIRI + panitumumab group for ORR (96%) and DCR (99%), FOLFIRI + bevacizumab + panitumumab group for OS (62%) and PFS (54%), and FOLFOXIRI + bevacizumab group for AEs grade ≥3 (59%) and SAEs (59%) outcomes.

**Conclusions:**

These findings suggest an available range of systemic treatment therapies with different efficacy and safety profiles with patients. Further investigations of the side effects and mutation status are required to confirm our findings.

**Systematic Review Registration:**

https://www.crd.york.ac.uk/prospero/, identifier CRD42019127772

**Graphical Abstract:**
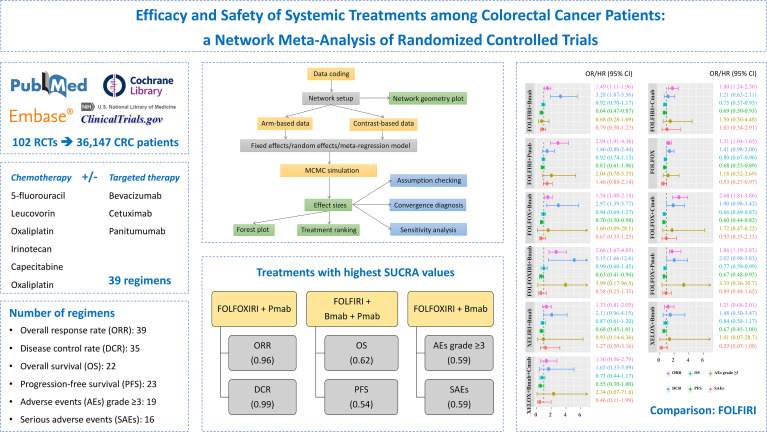


## Introduction

According to the most recent update in 2018, colorectal cancer (CRC) is still the third most common type of cancer and the second leading cause of cancer-related deaths worldwide (1). Currently, tumor resection is recommended for individuals with stage I, II, and III CRC (2). Additionally, adjuvant therapy is normally administered after completing surgery in an attempt to eliminate residual micrometastatic disease, which leads to decreased tumor recurrence and improved prolonged survival rate (3). When surgery is rarely indicated for unresectable metastatic CRC, several chemotherapy regimens are accepted for treatment (4). Among patients who are appropriate for intensive therapy with RAS mutations, 5-fluorouracil (5-FU) + leucovorin, 5-FU/leucovorin/oxaliplatin (FOLFOX), 5-FU/leucovorin/oxaliplatin/irinotecan (FOLFOXIRI), and capecitabine/oxaliplatin (XELOX) can be used alone or in combination with bevacizumab (Bmab) (4). For patients with a wild-type RAS genotype, cetuximab or panitumumab is combined with FOLFOX or 5-FU/leucovorin/irinotecan (FOLFIRI) (4). Infusions of 5-FU + leucovorin ± bevacizumab, and cetuximab (Cmab) or panitumumab (Pmab) are indicated for individuals for whom intensive therapy is not recommended and present with mutated and RAS wild-type genotypes, respectively (4).

A network meta-analysis (NMA) has been used to simultaneously compare multiple treatments by combining direct evidence from head-to-head or controlled trials and indirect results within a net-like relation to provide network evidence (5). Recently, an NMA focused on the efficacy of 17 regimens that do not differentiate the specific chemotherapies, especially capecitabine, 5-FU, leucovorin, irinotecan, and oxaliplatin, in each treatment comparison (6). Another study evaluated the efficacy of 10 regimens for first-line chemotherapy in patients with advanced CRC; however, their safety profiles have not been investigated (7). Therefore, by performing an NMA of randomized controlled trials (RCTs), we aimed to investigate the comparative efficacy and safety of several treatment regimens in patients with advanced or metastatic CRC. The results of the study are expected to provide reliable guidance for the selection of drugs in the treatment of CRC.

## Materials and Methods

### Search Strategy

This systematic search was conducted according to the Preferred Reporting Items for Systematic Reviews and Meta-Analyses (PRISMA) guidelines (8). We searched the PubMed, Embase, the Cochrane Library, and ClinicalTrials.gov databases from inception until June 30, 2021. The search was limited to human subjects and clinical trials. We also reviewed the bibliographies of relevant articles to identify additional studies related to the topic.

### Study Selection

The keywords used for the literature search were as follows: treatment (‘capecitabine’, ‘5-fluorouracil’, ‘leucovorin’, ‘irinotecan’, ‘bevacizumab’, ‘cetuximab’, ‘oxaliplatin’, and ‘panitumumab’) and CRC (‘colon cancer’, ‘rectal cancer’, and ‘colorectal cancer’). We included RCTs that (1) recruited patients with advanced or metastatic CRC; (2) investigated the efficacy and/or safety of combination therapies containing at least one regimen from the search; and (3) measured outcomes such as the hazard ratio (HR) for overall survival (OS) and progression-free survival (PFS), the overall response rate (ORR), the disease control rate (DCR), adverse events (AEs) grade ≥3, and serious adverse events (SAEs). Duplicate publications from the same study population were excluded.

Two authors independently reviewed the studies, discussed any controversies related to study selection, and extracted the information from the selected studies.

### Quality Assessment

The risk of bias for eligible studies was independently evaluated by two investigators in accordance with the Cochrane Handbook for Systematic Reviews of Intervention (9). Any discrepancies were discussed and resolved by consulting with other coauthors.

### Statistical Analysis

The pooled HRs for OS and PFS, the odds ratios (ORs) for the ORR, the DCR, AEs grade ≥3, and SAEs and their 95% credible intervals (CrIs) were calculated to evaluate the differences among regimens.

The node-splitting statistic was applied to assess the inconsistency assumption between direct pairwise meta-analyses and indirect estimates (10). The *I^2^* value was calcualated to test the heterogeneity among studies (10).

We applied the generalized linear model for Bayesian NMA, and the results of the random effects model were compared with those of the fixed effects model to compute the pooled estimates of outcomes (11). The treatment line was additionally considered as a covariate in the meta-regression model of the sensitivity analysis (12). Additionally, the convergence diagnosis of the Markov chain Monte Carlo (MCMC) with 50,000 burn-in iterations and 3 chains was used to obtain robust results. Detailed descriptions of the method are provided in the **Supplementary Appendix**. MCMC simulation analyses were performed using WinBUGS 1.4.3 software (MRC Biostatistics Unit, UK) with the Bayesian framework.

We determined the probabilities of being primary and secondary therapies for each treatment and the surface under the cumulative ranking curve (SUCRA), which ranges between 0 and 100%, for each outcome to calculate the probability of each treatment being the most effective (13). A higher SUCRA value indicates a greater likelihood that the treatment is closer to the top rank; in contrast, a lower SUCRA value indicates a greater likelihood that the treatment is closer to the bottom rank (14). The Spearman method was applied to calculate the pairwise correlation of outcomes, using the ‘psych’ R package (15). Enhanced k-means cluster analyses were used to group similar treatments with the ‘factoextra’ R package (16).

Detailed descriptions of the methodology are presented in the *eMethods*. The study methodology and progress were registered and approved by the National Institute for Health Research—International Prospective Register of Systematic Reviews (PROSPERO registration number: CRD42019127772).

## Results

### Selection of Eligible Studies

**Figure 1:**
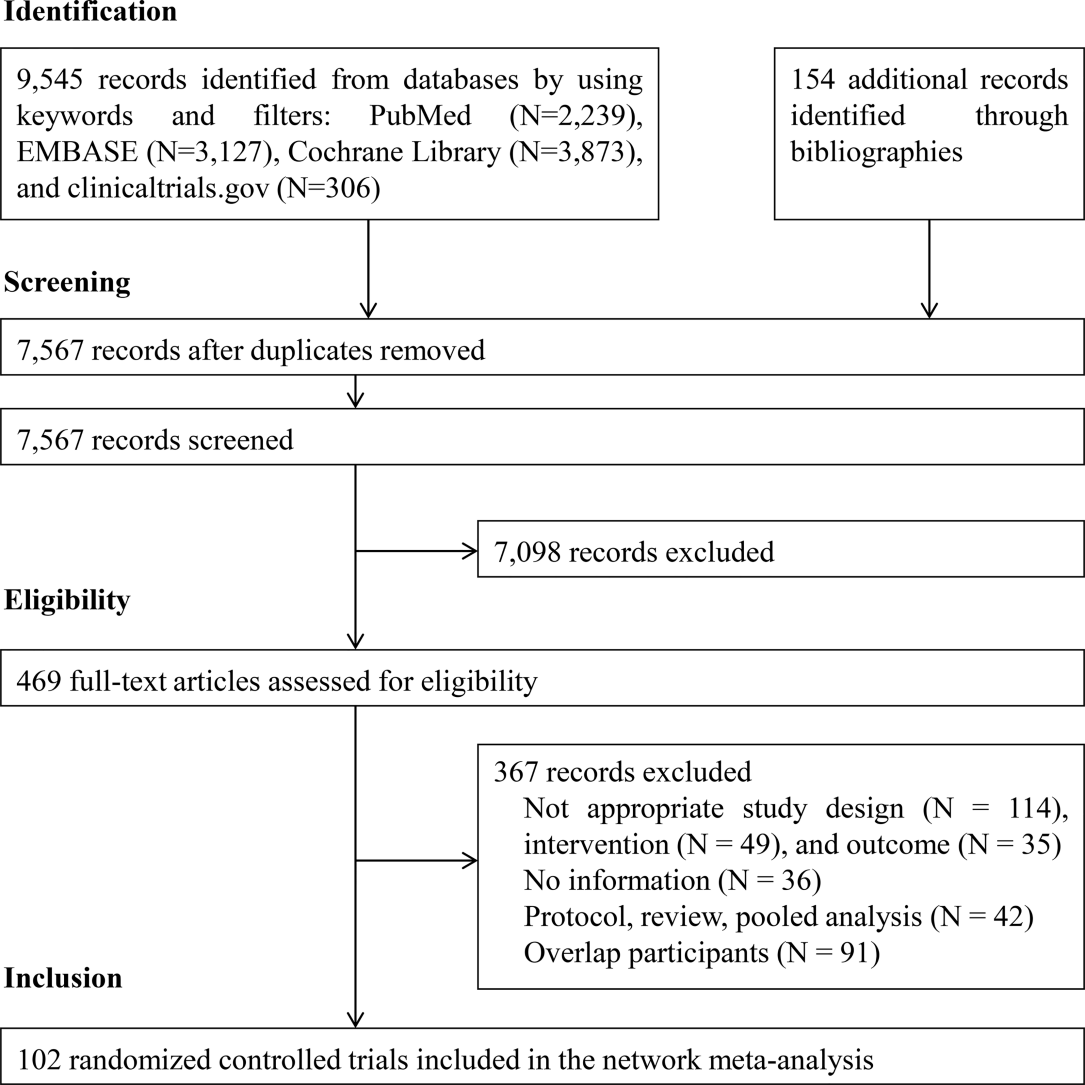
PRISMA flowchart for study selection.

**Figure 1** illustrates the PRISMA flowchart for the literature search and screening results. By searching four electronic databases and reviewing the bibliographies of five relevant cost-effectiveness analyses and NMAs, we identified 9,699 candidate reports. After screening the abstracts and titles, 469 articles remained and the full texts were assessed. Ultimately, 102 citations (92 main RCTs and 10 subgroup publications for additional outcome results) were included in the NMA (17–118).

### Characteristics of the Included Studies

Of all 94 main phase II/III RCTs, three were three-arm studies. The number of males was higher than the number of females in each research population, and the median age ranged between 60 and 70 years (**Supplementary Table S1**). Thirty-nine different regimens containing at least capecitabine, 5-FU, leucovorin, irinotecan, oxaliplatin, Bmab, Cmab, and Pmab were identified, and 39 treatments with 36,147 patients were included in the final analysis after excluding treatments not connected to the network (**Supplementary Table S2**).

### Cochrane Risk of Bias Assessment

**Figure 2:**
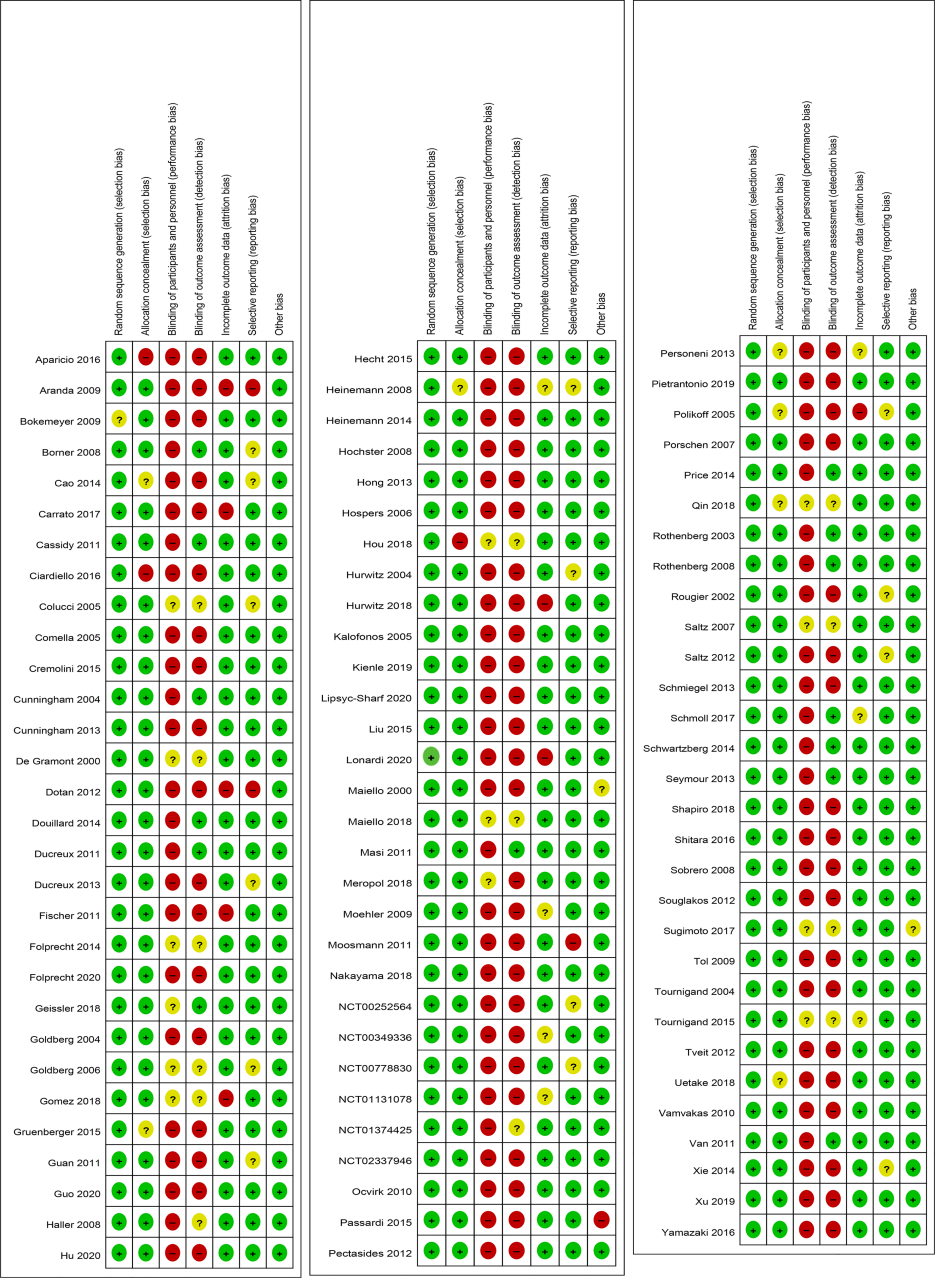
Risk of bias summary for each included study.

**Figure 3:**
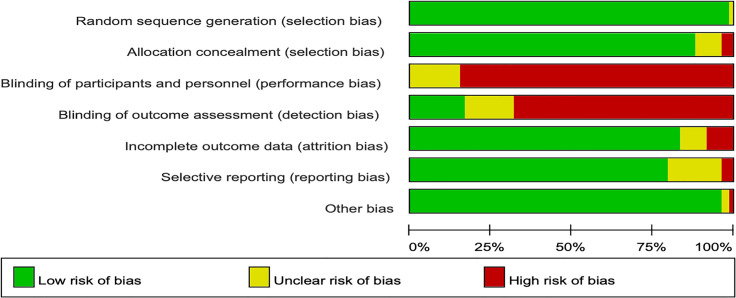
Risk of bias graph across all the included studies.

In the Cochrane risk of bias assessment, most of the RCTs originated from multicenter trials in which randomization was stratified by different baseline factors, thus minimizing selection bias. In contrast, an open-label design led to a high or unclear risk of performance bias in all the RCTs (**Figures 2**, **3**).

### Network Geometry of Available Evidence

**Figure 4:**
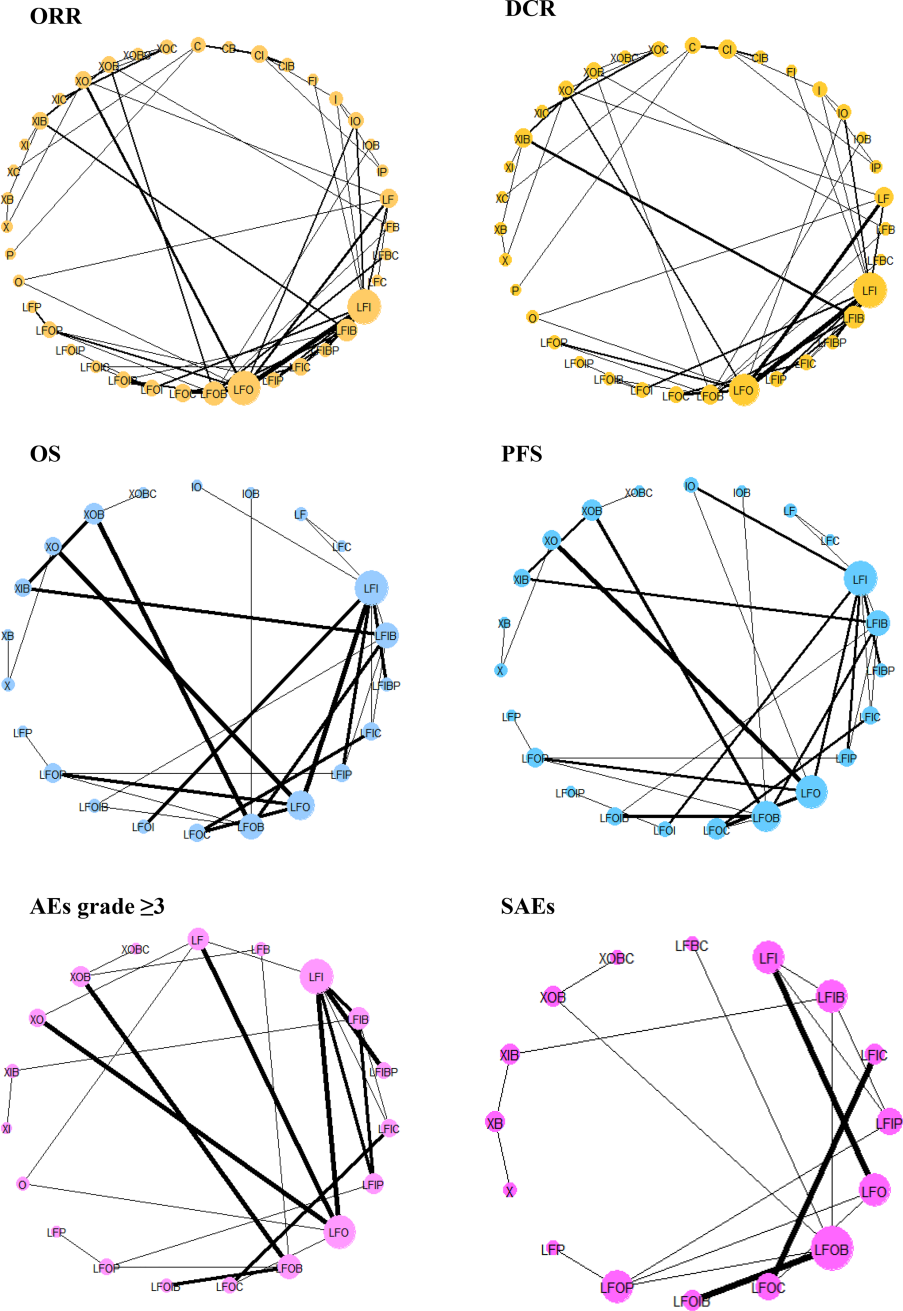
Network geometry of treatments included in the final analysis. ORR, overall response rate; DCR, disease control rate; OS, overall survival; PFS, progression-free survival; AEs, adverse events; SAEs, serious adverse events; X, capecitabine; B, bevacizumab; C, cetuximab; F, 5-fluorouracil; I, irinotecan; L, leucovorin; O, oxaliplatin; P, panitumumab. The network plots are based on treatments in a connected network according to different types of outcomes. The size of the node indicates the number of participants receiving each treatment, and the thickness of the line illustrates the number of studies included each comparison.

**Supplementary Figure S1** shows the network geometry of all the available comparisons of treatments to explore their relationships and contributions. Among them, treatments for which data on specific outcomes were not available or did not contribute to the main network were excluded (**Supplementary Table S3**). As a result, pairwise comparisons of 39, 35, 22, 23, 19, and 16 systemic therapies regarding the ORR, the DCR, OS, PFS, AEs grade ≥3, and SAEs, respectively, were eligible for the final analysis (**Figure 4**).

### Forest Plots of the Pooled Estimates of 11 Regimens

**Figure 5:**
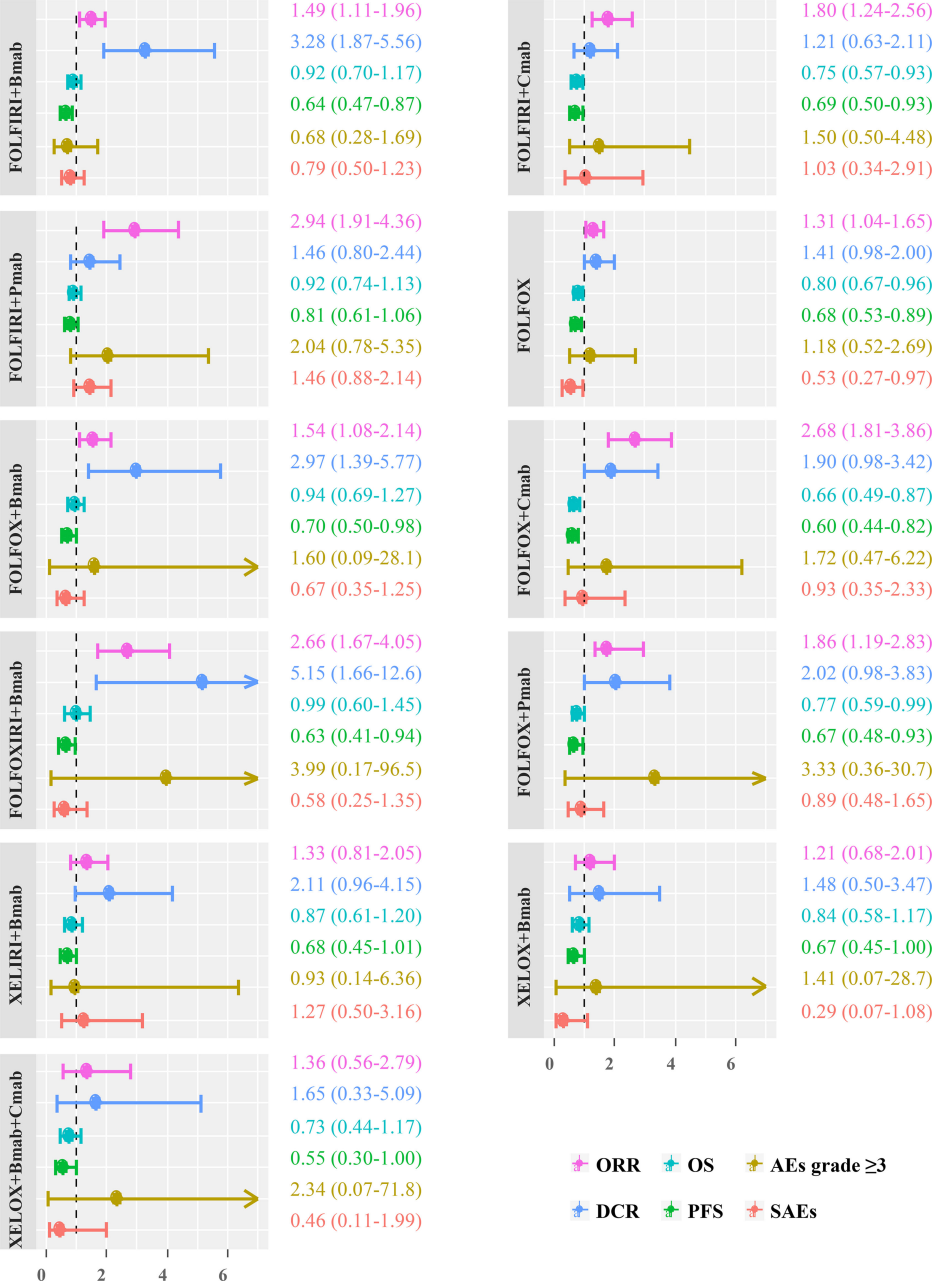
Scatter plot showing differences in estimates obtained from fixed-effects, random-effects, and meta-regression models. ORR, overall response rate; DCR, disease control rate; OS, overall survival; PFS, progression-free survival; AEs, adverse events; SAEs, serious adverse events; FOLFIRI, 5-fluorouracil/leucovorin/irinotecan; FOLFOX, 5-fluorouracil/leucovorin/oxaliplatin; FOLFOFIRI, 5-fluorouracil/leucovorin/oxaliplatin/irinotecan; XELOX, capecitabine/oxaliplatin; XELIRI, capecitabine/irinotecan; Bmab, bevacizumab; Cmab, cetuximab; Pmab, panitumumab.

The comparative treatment effects of 11 regimens for which the results were available on six outcomes relative to the FOLFIRI combination are shown in **Figure 5**. Regarding the ORR, FOLFIRI + Bmab/Cmab/Pmab, FOLFOX, FOLFOX + Bmab/Cmab/Pmab, and FOLFOXIXI + Bmab resulted in a significant improvement, with ORs (95% CrIs) of 1.49 (1.11–1.96), 1.80 (1.24–2.56), 2.94 (1.91–4.36), 1.31 (1.04–1.65), 1.54 (1.08–2.14), 2.68 (1.81–3.86), 1.86 (1.19–2.83), and 2.66 (1.67–4.05), respectively. Regarding the DCR, the ORs (95% CrIs) of FOLFIRI/FOLFOX/FOLFOXIRI + Bmab compared to FOLFIRI were 3.28 (1.87–5.56), 2.97 (1.39–5.77), and 5.15 (1.66–12.6), respectively. Regarding survival outcomes, FOLFIRI + Cmab (HR = 0.75, 95% CrI = 0.57–0.93 for OS and HR = 0.69, 95% CrI = 0.50–0.93 for PFS), FOLFOX (HR = 0.80, 95% CrI = 0.67–0.96 for OS and HR = 0.68, 95% CrI = 0.53–0.89 for PFS), FOLFOX + Cmab (HR = 0.66, 95% CrI = 0.49–0.87 for OS and HR = 0.60, 95% CrI = 0.44–0.82 for PFS), and FOLFOX + Pmab (HR = 0.77, 95% CrI = 0.59–0.99 for OS and HR = 0.67, 95% CrI = 0.48–0.93 for PFS) prolonged both OS and PFS compared with FOLFIRI. PFS was significantly longer in patients treated with the FOLFIRI + Bmab, FOLFOX + Bmab, and FOLFOXIRI + Bmab regimens (36, 30, and 37%, respectively) than in patients treated with the FOLFIRI regimen. In terms of safety endpoints, including AEs grade ≥3 and SAEs, the treatment effects were comparable between the 11 treatments and FOLFIRI; however, a 47% lower probability of SAEs was observed in the FOLFOX treatment group relative to the FOLFIRI treatment group (OR = 0.53, 95% CrI = 0.27–0.97). The complete results for pairwise comparisons of all the six outcomes are available in **Supplementary Tables S4–S6**.

### Inconsistency and Heterogeneity Assumption

In the inconsistency analysis, no significant differences were observed between direct and indirect estimates of PFS, AEs grade ≥3, and SAEs outcomes (**Supplementary Table S7**). The statistical inconsistency between direct and indirect estimates of the ORR for FOLFIRI + Pmab vs FOLFIRI + Bmab (p = 0.01) and FOLFOX + Pmab vs FOLFIRI + Pmab (p *=* 0.01); the DCR for FOLFOX + Pmab vs. FOLFOX (p = 0.04), XELOX + Cmab vs. XELOX (p = 0.01), XELOX + Bmab + Cmab vs. XELOX + Bmab (p = 0.01), and XELOX + Bmab + Cmab vs. XELOX + Cmab (p = 0.004), and the OS for FOLFOXIRI + Bmab vs. FOLFIRI + Bmab (p *=* 0.01) and FOLFOXIRI + Bmab vs. FOLFOX + Bmab (p *=* 0.01) was observed in the node-splitting model. The between-trial variance is reported as the global *I^2^* value in both pairwise and consistency estimates, and the substantial heterogeneity is reported as a measure of ORR (*I^2^ =* 39%), DCR (*I^2^ =* 32%), OS (*I^2^ =* 30%), PFS (*I^2^ =* 69%), and AEs grade ≥3 (*I^2^ =* 84%) for pairwise *I^2^* and ORR (*I^2^ =* 38%), DCR (*I^2^ =* 49%), OS (*I^2^* = 53%), PFS (*I^2^* = 70%), and AEs grade ≥3 (*I^2^* = 81%) for consistent *I^2^* (**Supplementary Table S8**).

### Sensitivity Analysis

**Figure 6:**
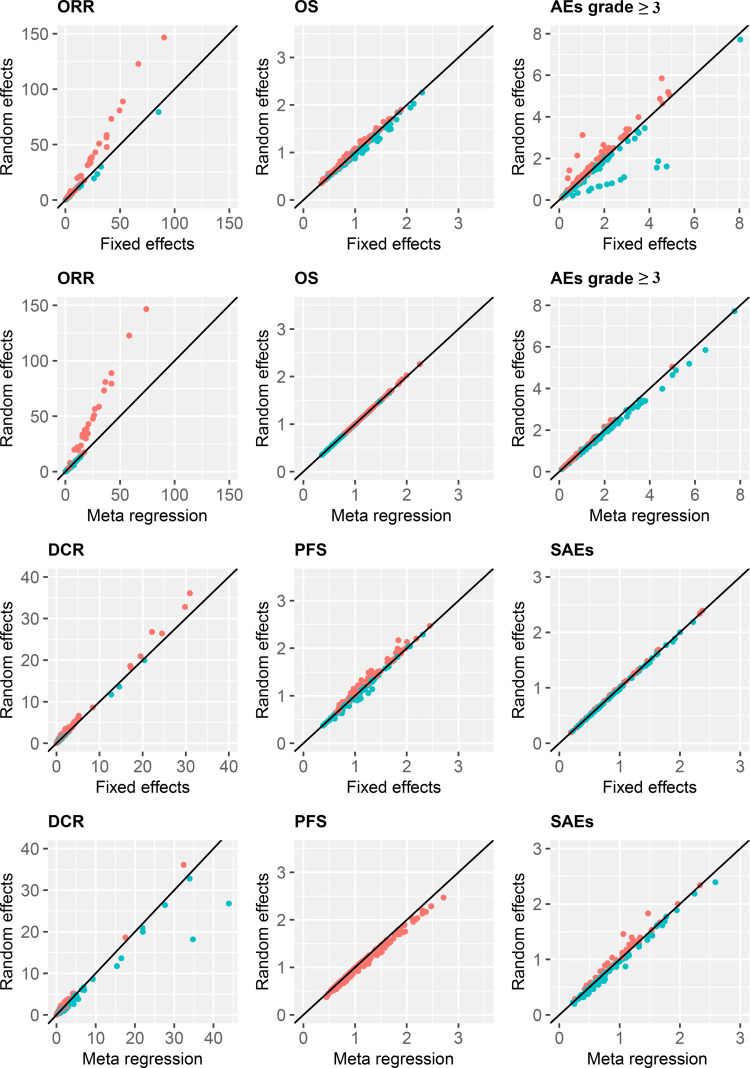
Treatment efficacy and safety compared with FOLFIRI. ORR, overall response rate; DCR, disease control rate; OS, overall survival; PFS, progression-free survival; AEs, adverse events; SAEs, serious adverse events.

The pooled ORs/HRs for all the effect sizes from the random effects model were compared with the those of the fixed effects model and the meta-regression of treatment line covariates in a scatter plot with the slope of the straight line equal to one. The results were similar among the three models, except for some estimates of ORR and DCR outcomes for the comparison of the random-effects model with both fixed-effects and meta-regression models (**Figure 6**). Thus, the estimates obtained after including the treatment line in the meta-regression model did not differ substantially from those in the random-effects model.

Parameters for the comparison of model performance of fixed-effects, random-effects, and meta-regression models are shown in **Table 1**. Overall, the deviance information criteria values in the random effects models were relatively lower than those in the fixed effects and meta-regression models, which suggested better model performance.

**Table 1:** Parameters used for model selection.

Outcome	Parameter	Fixed effects	Random effects	Meta-regression
Overall response rate	Posterior mean residual deviance, ${\bar{D}}_{res}$	253	203	202.1
	Posterior mean deviances, ${\bar{D}}_{model}$	1,179.01	1,128.59	1,128.44
	Effective number of parameters, *p_D_*	133.36	160.774	161.692
	Between-study standard deviation, σ		0.283	0.286
	Deviance information criteria, *DIC*	1,312.37	1,289.36	1,290.13
Disease control rate	Posterior mean residual deviance, ${\bar{D}}_{res}$	203.4		160.2
	Posterior mean deviances, ${\bar{D}}_{model}$	860.277	811.14	816.581
	Effective number of parameters, *p_D_*	104.06	126.235	123.259
	Between-study standard deviation, σ		0.409	0.316
	Deviance information criteria, *DIC*	964.337	937.374	939.84
Overall survival	Posterior mean residual deviance, ${\bar{D}}_{res}$	69.15	50.93	51.3
	Posterior mean deviances, ${\bar{D}}_{model}$	−21.076	−39.326	−38.918
	Effective number of parameters, *p_D_*	20.992	32.378	32.296
	Between-study standard deviation, σ		0.017	0.026
	Deviance information criteria, *DIC*	−0.084	−6.948	−6.622
Progression-free survival	Posterior mean residual deviance, ${\bar{D}}_{res}$	109.2	51.25	51.22
	Posterior mean deviances, ${\bar{D}}_{model}$	1.442	−56.466	−56.488
	Effective number of parameters, *p_D_*	21.982	40.924	40.944
	Between-study standard deviation, σ		0.193	0.193
	Deviance information criteria, *DIC*	23.424	−15.542	−15.544
Adverse events grade ≥3	Posterior mean residual deviance, ${\bar{D}}_{res}$	186.3	71.62	71.53
	Posterior mean deviances, ${\bar{D}}_{model}$	537.81	423.174	423.094
	Effective number of parameters, *p_D_*	52.067	68.344	68.582
	Between-study standard deviation, σ		0.742	0.768
	Deviance information criteria, *DIC*	589.877	491.519	491.676
Serious adverse events	Posterior mean residual deviance, ${\bar{D}}_{res}$	39.09	39.65	39.44
	Posterior mean deviances, ${\bar{D}}_{model}$	250.384	250.95	250.741
	Effective number of parameters, *p_D_*	36.036	37.597	38.364
	Between-study standard deviation, σ		0.136	0.159
	Deviance information criteria, *DIC*	286.42	288.547	289.106

### Treatment Ranking Probability and SUCRA Clustering Analysis

**Figure 7:**
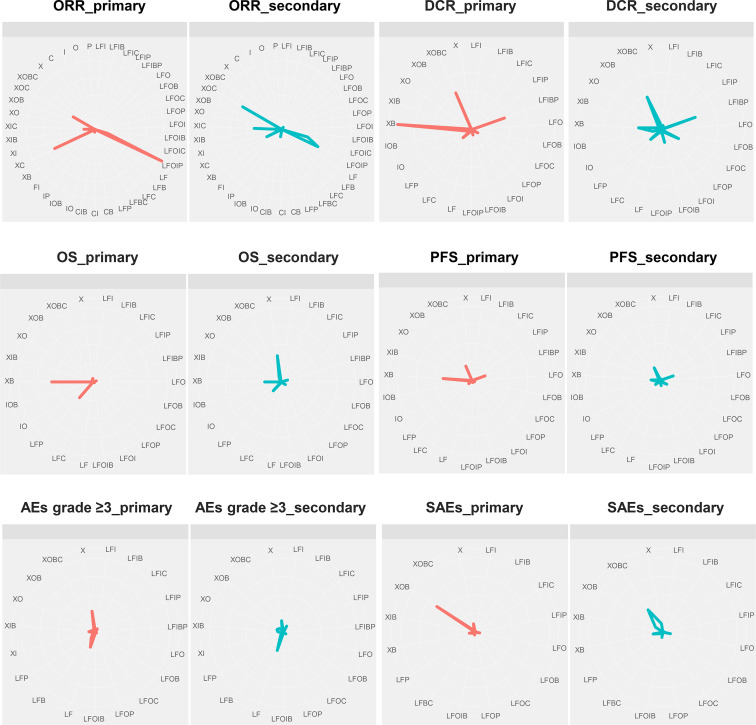
First-line and second-line treatment ranking probabilities. ORR, overall response rate; DCR, disease control rate; OS, overall survival; PFS, progression-free survival; AEs, adverse events; SAEs, serious adverse events; X, capecitabine; B, bevacizumab; C, cetuximab; F, 5-fluorouracil; I, irinotecan; L, leucovorin; O, oxaliplatin; P, panitumumab.

**Figure 7** and **Supplementary Table S9** show the probabilities of each regimen being used as primary and secondary options for the treatment of advanced or metastatic CRC. Compared to the other regimens, FOLFOXIRI + Pmab had the highest probability of becoming a first-line candidate that improved both the ORR (43%) and the DCR (86%). In addition, XELOX + Cmab and FOLFOXIRI + Bmab were most likely to be second-line therapies in terms of improving the ORR (26%) and DCR (47%), respectively. When considering OS and PFS, capecitabine + Bmab had the highest probability of becoming a first-line candidate, with ranking probabilities of 56% for OS and 39% for PFS. Furthermore, capecitabine and FOLFIRI + Bmab + Pmab were considered to be the second-line candidates for prolonging OS (36%) and PFS (19%), respectively, and had the highest probability of becoming a first-line candidate for prolonging OS (56%) and PFS (39%). A similar trend was observed for AEs grade ≥3, with a value of 29% for oxaliplatin followed by 21% for leucovorin + 5-FU as a first-line therapy and 26% for leucovorin + 5-FU and 16% for oxaliplatin as a second-line therapy. Although XELOX + Bmab had the greatest probability of decreasing the number of SAEs when used as a primary treatment (62%), XELOX + Bmab + Cmab was suggested as a secondary therapy (36%).

**Figure 8:**
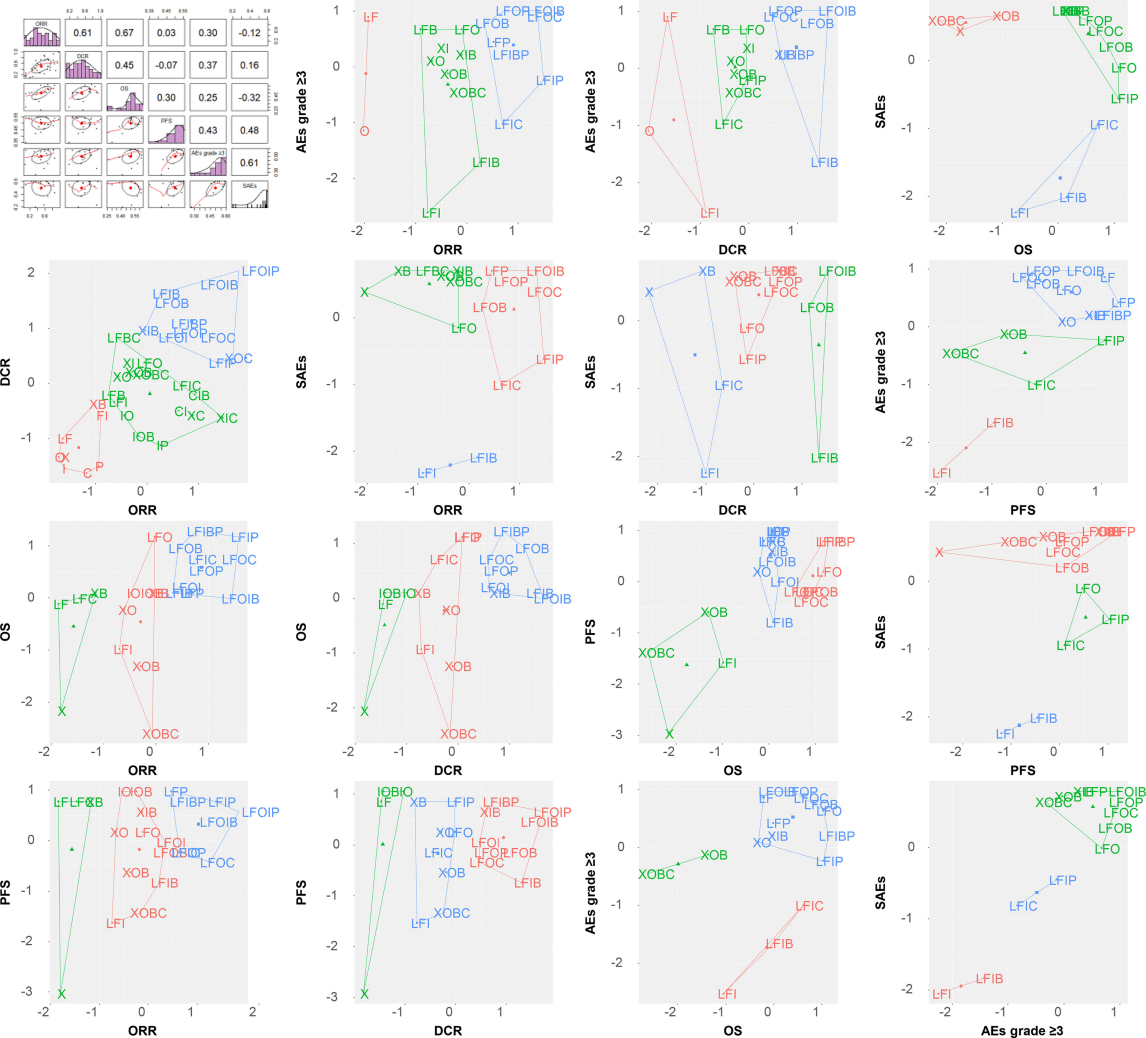
SUCRA ranking plot and cluster analysis. ORR, overall response rate; DCR, disease control rate; OS, overall survival; PFS, progression-free survival; AEs, adverse events; SAEs, serious adverse events; X, capecitabine; B, bevacizumab; C, cetuximab; F, 5-fluorouracil; I, irinotecan; L, leucovorin; O, oxaliplatin; P, panitumumab.

The SUCRA value, which summarizes the cumulative ranking of the treatment line, was calculated for each treatment (**Table 2**). The highest SUCRA values were observed for ORR (96%) and DCR (99%) in patients treated with FOLFOXI + Pmab, OS (62%) and PFS (54%) in patients treated with FOLFIRI + Bmab + Pmab, and AEs grade ≥3 (59%) and SAEs (59%) in patients treated with FOLFOXIRI + Bmab. **Figure 8** displays the association between the SUCRA value and each pair of outcomes. Substantial Spearman’s correlations were observed between the SUCRA values for ORR and DCR (0.61), ORR and OS (0.67), and AEs grade ≥3 and SAEs (0.61). The enhanced k-means cluster analysis showed that the groups of treatments exerted a similar pairwise effect on the outcomes. Overall, treatments with higher SUCRA values were located in the upper right quadrant of the plot; in contrast, those with lower SUCRA values were located in the lower left quadrant of the plot.

**Table 2:** SUCRA rankings for colorectal cancer treatments.

Treatment	ORR	DCR	OS	PFS	AEs grade ≥3	SAEs
LFI	0.34	0.42	0.41	0.42	0.27	0.20
LFIB	0.57	0.89	0.51	0.46	0.35	0.23
LFIC	0.67	0.49	0.57	0.49	0.41	0.37
LFIP	0.85	0.59	0.61	0.54	0.48	0.42
LFIBP	0.67	0.76	0.62	0.54	0.52	
LFO	0.49	0.59	0.61	0.51	0.56	0.48
LFOB	0.59	0.85	0.59	0.49	0.57	0.52
LFOC	0.82	0.70	0.57	0.48	0.58	0.55
LFOP	0.68	0.72	0.55	0.49	0.59	0.57
LFOI	0.60	0.70	0.52	0.50		
LFOIB	0.82	0.93	0.50	0.52	0.59	0.59
LFOIC	0.88					
LFOIP	0.96	0.99		0.53		
LF	0.08	0.26	0.49	0.54	0.58	
LFB	0.31	0.45			0.56	
LFC	0.17		0.50	0.54		
LFBC	0.35	0.70				0.59
LFP	0.63		0.51	0.55	0.54	0.59
CB	0.31					
CI	0.66	0.38				
CIB	0.74	0.45				
IO	0.39	0.36	0.51	0.55		
IOB	0.46	0.27	0.51	0.55		
IP	0.57	0.23				
FI	0.27	0.36				
XB	0.24	0.41	0.51	0.54		0.59
XC	0.73	0.36				
XI	0.40	0.59			0.53	
XIB	0.49	0.73	0.51	0.53	0.52	0.59
XIC	0.88	0.35				
XO	0.36	0.53	0.48	0.51	0.51	
XOB	0.43	0.55	0.38	0.47	0.49	0.58
XOC	0.93	0.61				
XOBC	0.48	0.54	0.26	0.43	0.46	0.57
X	0.09	0.18	0.30	0.35		0.55
C	0.20	0.11				
I	0.09	0.13				
O	0.06	0.18			0.40	
P	0.26	0.14				

ORR, overall response rate; DCR, disease control rate; OS, overall survival; PFS, progression-free survival; AEs, adverse events; SAEs, serious adverse events; X, capecitabine; B, bevacizumab; C, cetuximab; F, 5-fluorouracil; I, irinotecan; L, leucovorin; O, oxaliplatin; P, panitumumab.

## Discussion

This study investigated the comparative efficacy and safety of different regimens used to treat advanced or metastatic CRC. The pairwise treatment effects of 39, 35, 22, 23, 19, and 16 systemic therapies on the ORR, DCR, OS, PFS, AEs grade ≥3, and SAEs were compared using a comprehensive NMA. Compared with the FOLFIRI regimen, which was administered to the greatest number of participants, the other 9 treatments, namely, FOLFIRI + (Bmab or Cmab or Pmab), FOLFOX ± Bmab/Cmab/Pmab, and XELIRI/XELOX + Bmab, significantly improved at least one outcome. The highest probabilities of being used as a first-line treatment to improve ORR, DCR, OS, PFS, AEs grade ≥3, and SAEs were reported for FOLFOXIRI + Pmab, FOLFOXIRI + Pmab, capecitabine + Bmab, capecitabine + Bmab, oxaliplatin, and XELOX + Bmab, respectively.

In this study, the replacement of irinotecan in the FOLFIRI regimen by oxaliplatin in the FOLFOX combination resulted in a higher efficacy of prolonging OS and PFS but a lower safety profile of SAEs. Although the efficacy and safety of XELIRI vs. XELOX, XELIRI + Bmab vs. XELOX + Cmab, and irinotecan vs. oxaliplatin were not evaluated, the treatment effects of other irinotecan-based vs. oxaliplatin-based regimens, such as FOLFIRI + Bmab/Cmab/Pmab vs. FOLFOX + Bmab/Cmab/Pmab, were comparable. The ORR, DCR, and AEs grade ≥3 were not significantly different between all the irinotecan-based and oxaliplatin-based treatments (**Supplementary Tables 4–6**).

Compared with the common regimens, namely, FOLFOX, FOLFIRI, and FOLFOXIRI, the addition of Bmab and/or Cmab and/or Pmab to these regimens resulted in a significant improvement in some treatment outcomes. Compared with FOLFIRI, FOLFIRI + Bmab/Cmab/Pmab increased the ORR, FOLFIRI + Bmab increased the DCR, and FOLFIRI + Cmab and FOLFIRI + Bmab + Pmab prolonged both OS and PFS, whereas FOLFIRI + Bmab prolonged PFS only, but safety outcomes were not significantly different. In addition, FOLFOX + Pmab was associated with a greater number of SAEs than FOLFOX. The ORR and the DCR of the FOLFOXIRI + Bmab group were better than those of the FOLFIRI group, and FOLFOXIRI + Pmab exerted better effects on the ORR and the DCR than did FOLFIRI, FOLFOX, and FOLFOXIRI (**Supplementary Tables 4–6**). The greater the number of drugs in the combination regimen, the higher the potential efficacy; however, the safety profile might remain similar.

Ba-Sang et al. reported the comparative response of patients to 17 regimens using an NMA and suggested the potential effect of Bmab + chemotherapy and Pmab + chemotherapy regimens compared with other targeted therapies (6). Another NMA of 10 regimens recommended considering FOLFIRI and FOLFOX as first-line therapies for short-term and long-term advanced CRC treatments (7). According to the US Food and Drug Administration, FOLFOX is indicated for the treatment of adjuvant stage III or advanced CRC, whereas FOLFIRI is approved as a first-line treatment for metastatic CRC (119, 120). However, in the current NMA, the prolongation of OS and PFS by FOLFOX relative to FOLFIRI suggests a possible indication of FOLFOX for metastatic CRC. Furthermore, by implementing different approaches and selecting regimens using the NMA, the current study focused on the commonly used drugs in clinical practice and suggested that not only FOLFIRI and FOLFOX but also their combination with Bmab or Cmab or Pmab into these regimens might result in superior efficacy or safety. Consistent with our findings, Bmab, which is a vascular endothelial growth factor (VEGF) monoclonal antibody, is also indicated as a second-line treatment for metastatic CRC in combination with 5-FU + irinotecan-based or 5-FU + oxaliplatin-based chemotherapy (121). Other epidermal growth factor receptor (EGFR) inhibitors, namely, Cmab and Pmab, can be administered as single agents after the failure of irinotecan-based and oxaliplatin-based regimens (122, 123). Similar findings were observed upon comparing the ORR and the DCR of Cmab or Pmab vs FOLFIRI or FOLFOX, Cmab vs. FOLFIRI + Cmab or FOLFOX + Cmab, and Pmab vs. FOLFIRI + Pmab or FOLFOX + Pmab regimens. Although data for survival and safety outcomes of patients treated with Cmab or Pmab are available, they do not contribute to the connection to the main network; thus, these treatment effects were not evaluated.

Given the systemic toxicity and unpredictable resistance to chemotherapy, Xie et al. recently conducted a comprehensive review of targeted therapy for patients with CRC (124). Most currently approved agents for patients with advanced-stage metastatic disease target EGFR- and VEGF-related pathways, namely, Cmab and Pmab as EGFR inhibitors, vemurafenib as a BRAF inhibitor, dabrafenib + trametinib and encorafenib + binimetinib as MEK inhibitors, and Bmab, regorafenib, ziv-aflibercept, and ramucirumab as treatments targeting angiogenesis (124). Among them, anti-EGFR agents are recommended for patients with left-side metastatic CRC with the wild-type RAS genotype only, whereas anti-VEGF agents can be used regardless of the RAS mutation status (124). Furthermore, a 10% of BRAF (with common V600E substitution) mutation rate was reported in metastatic CRC (125). For these BRAF-mutated patients, chemotherapy + Bmab and FOLFOXIRI + Bmab showed good performance as a first-line treatment (125). However, regardless of the side and mutation status, our study determined the high efficacy of FOLFOXIRI + Pmab in terms of response and FOLFIRI + Bmab + Pmab in terms of survival outcomes. We also reported FOLFOXIRI + Bmab as a treatment with high safety.

To the knowledge of the authors, this study is the first to investigate both the efficacy and safety of common drugs used to treat advanced or metastatic CRC. The individual studies were derived from a large number of RCTs and participants. Efficacy and safety were investigated through different surrogates to yield consistent findings. The statistical analysis was conducted using the Bayesian approach, with the appropriate prior distribution of variables, which produced reliable results. Bayesian assumptions, such as consistency and heterogeneity, were checked and reported. A sensitivity analysis among three core models was also performed to obtain robust estimates.

Despite these advantages, the current NMA has some limitations. First, due to a lack of head-to-head trials, several treatments were excluded from the network of different outcomes. Second, the combination of treatments with different dosage forms and the treatment plan might have affected the final estimates. Third, the limitation of the meta-analysis in considering differences across studies due to patient characteristics must be addressed. Recent methods have been proposed to account the heterogeneity between trial results in meta-analyses (126, 127); however, the application of NMA requires further investigation. Fourth, the current study did not yield an ideal index for both efficacy and safety from six evaluated endpoints to select the ideal regimen for the treatment of advanced or metastatic CRC. Additional research data and an optimized algorithm are required to correct this deviation (128, 129).

In summary, we performed an NMA of available RCTs to investigate the efficacy and safety of various therapies for advanced or metastatic CRC. We observed good efficacy of FOLFOXIRI + Pmab in terms of response and FOLFIRI + Bmab + Pmab in terms of survival outcomes and good safety profile for FOLFOXIRI + Bmab. We propose that this overview provides the most appropriate evidence for a range of efficient therapies and may inform clinical practice and decision making and aid in the planning for future research. Further studies may develop a novel endpoint that considers both the efficacy and safety of regimens and consider the side and mutation status to confirm our findings.

## Data Availability Statement

The original contributions presented in the study are included in the article/**Supplementary Material**. Further inquiries can be directed to the corresponding author.

## Author Contributions

We greatly appreciate the efforts of those individuals who contributed to this study. The authors’ responsibilities were as follows: TH and JK designed the research. TH, DS, BK, YC, and JK conducted the research. TH analyzed the data. TH and JK wrote the paper and are primarily responsible for the final content. All authors contributed to the article and approved the submitted version.

## Funding

This work was supported by grants from the National Cancer Center (Nos. 1710882 and 2210990).

## Conflict of Interest

The authors declare that the research was conducted in the absence of any commercial or financial relationships that could be construed as a potential conflict of interest.

## Publisher’s Note

All claims expressed in this article are solely those of the authors and do not necessarily represent those of their affiliated organizations, or those of the publisher, the editors and the reviewers. Any product that may be evaluated in this article, or claim that may be made by its manufacturer, is not guaranteed or endorsed by the publisher.

## Abbreviations

CRC, colorectal cancer; NMA, network meta-analysis; OR, odds ratio; HR, hazard ratio; CrI, credible interval; ORR, overall response rate; DCR, disease control rate; OS, overall survival; PFS, progression-free survival; AEs, adverse events; SAEs, serious adverse events; FOLFIRI, 5-fluorouracil/leucovorin/irinotecan; FOLFOX, 5-fluorouracil/leucovorin/oxaliplatin; FOLFOFIRI, 5-fluorouracil/leucovorin/oxaliplatin/irinotecan; XELOX, capecitabine/oxaliplatin; XELIRI, capecitabine/irinotecan; 5-FU, 5-fluorouracil; Bmab, bevacizumab; Cmab, cetuximab; Pmab, panitumumab.
