# Supplementary material for: Efficacy and Safety of Systemic Treatments Among Colorectal Cancer Patients: A Network Meta-Analysis of Randomized Controlled Trials
Source: Front Oncol. 2022 Feb 9;11:756214. doi: 10.3389/fonc.2021.756214 (PMC8864322; doi:10.3389/fonc.2021.756214)
Supplement: Supplementary file 1 [file DataSheet_1.pdf]

eMethods.

1. Search strategy from databases

1.1 Pubmed and Cochrane library

- Search syntax: (colon cancer OR rectal cancer OR colorectal cancer) AND (capecitabine OR 5-fluorouracil OR leucovorin OR irinotecan OR bevacizumab OR cetuximab OR oxaliplatin OR panitumumab) AND rando*
- Search filter: clinical trial and human

1.2 Embase

- Search syntax: ('colon cancer' OR 'rectal cancer' OR 'colorectal cancer') AND (capecitabine OR 5-fluorouracil OR leucovorin OR irinotecan OR bevacizumab OR cetuximab OR oxaliplatin OR panitumumab) AND rando*
- Search filter: clinical trial and human

1.3 Clinicaltrials.gov

- Search syntax: colorectal cancer AND (capecitabine OR 5-fluorouracil OR leucovorin OR irinotecan OR bevacizumab OR cetuximab OR oxaliplatin OR panitumumab)
- Search filter: having result studies

1.4 Bibliography

(1) Ba-Sang DZ, Long ZW, Teng H, Zhao XP, Qiu J, Li MS. A network meta-analysis on the efficacy of sixteen targeted drugs in combination with chemotherapy for treatment of advanced/metastatic colorectal cancer. *Oncotarget* 2016;7: 84468-79.

(2) Mocellin S, Baretta Z, Roque IFM, Solà I, Martin0Richard M, Hallum S, et al. Second-line systemic therapy for metastatic colorectal cancer. *Cochrane Database Syst Rev* 2017;1:CD006875.

(3) Petrelli F, Ardito R, Ghidini A, Zaniboni A, Ghidini M, Barni S, et al. Different Toxicity of Cetuximab and Panitumumab in Metastatic Colorectal Cancer Treatment: A Systematic Review and Meta-Analysis. *Oncology* 2018;94: 191-9.

(4) Tikhonova IA, Huxley N, Snowsill T, Crathorne L, Varley-Campbell J, Napier M, et al. Economic Analysis of First-Line Treatment with Cetuximab or Panitumumab for RAS Wild-Type Metastatic Colorectal Cancer in England. *PharmacoEconomics* 2018; 36:837-51.

(5) Wu DM, Wang YJ, Fan SH, Zhuang ZF, Shan Q, Han XR, et al. Network meta-analysis of the efficacy of first-line chemotherapy regimens in patients with advanced colorectal cancer. *Oncotarget* 2017;8: 100668-77.

2. Scheme for data analysis

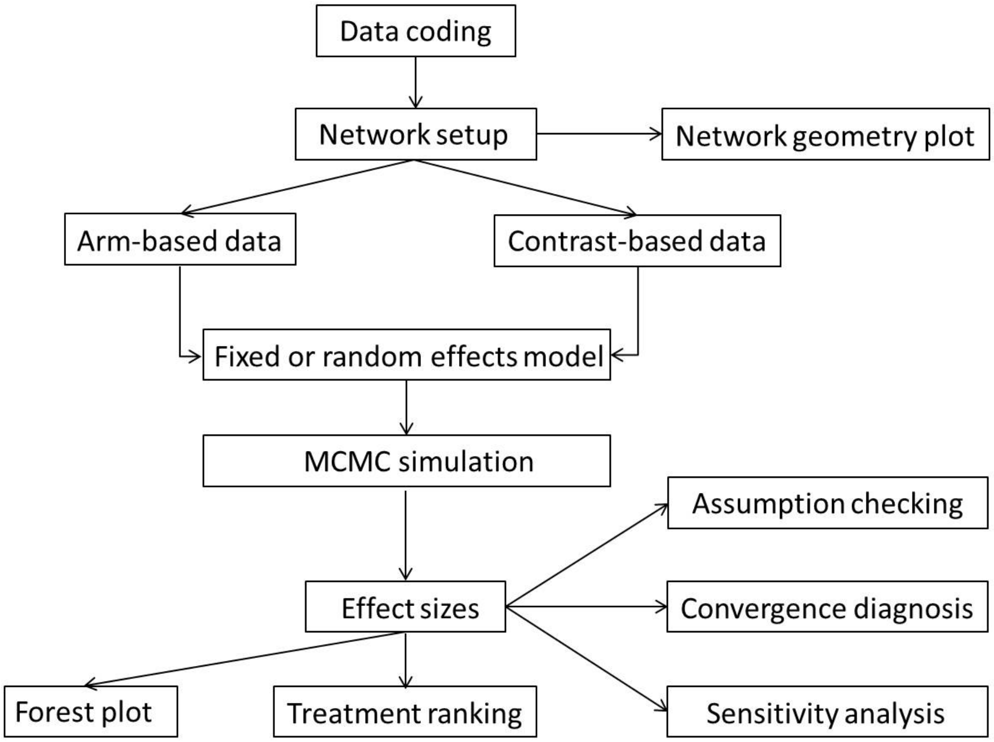

In the current study, arm-based data provides the absolute effect of each treatment arm for overall response rate (ORR), disease control rate (DCR), adverse events (AEs) grade ≥3, and serious adverse events (SAEs) outcomes, while contrast-based data performs the relative effect between treatment arms for overall survival (OS) and progression-free survival (PFS) outcomes.

3. Model identification and parameters

The current network meta-analysis takes the form as generalized linear model (GLM):[1]

$$g(\gamma) = \theta_{ik} = \mu_i + \delta_{ik}$$

where g represents the appropriate link function, θ_{ik} is the linear predictor, which is simply a regression model with S-1 treatment effect parameters for the network of S treatments, μ_i represent the trial-specific effects of treatment in arm 1 of trial i, and δ_{ik} represent the trial-specific effects of treatment in arm k compared with the treatment in arm 1 in the same trial:

$$\delta_{ik} \sim Normal\left(d_{t_{i1},t_{ik}}, \sigma^2\right)$$

with $d_{t_{i1},t_{ik}} = d_{1,t_{ik}} - d_{1,t_{i1}}$ is the mean effect of treatment in arm k in trial i, t_{ik}, compared with the treatment in arm 1, t_{1k}, and σ^2 is the between-trial variability in treatment effects.

3.1 Fixed effects model

In the fixed effects model,[1] when there is no between-trial heterogeneity ($\sigma^2 = 0$), the GLM formula can be written as:

$$\theta_{ik} = \mu_i + d_{t_{i1}t_{ik}} = \mu_i + d_{1,t_{ik}} - d_{1,t_{i1}}$$

3.1.1 Arm-based data

For binomial data (ORR, DCR, AE grade ≥3, and SAE outcomes), the probabilities of event p_{ik} are modeled on the logit scales as:

$$logit(p_{ik}) = \mu_i + d_{1,t_{ik}} - d_{1,t_{i1}}$$

where the number of events r_{ik} and the number of patients n_{ik} in arm k of trial i follow the binomial likelihood:

$$r_{ik} \sim Binomial(p_{ik}, n_{ik})$$

3.1.2 Contrast-based data

For continuous data (OS and PFS outcomes), there is no trial-specific effect on control treatment and the linear predictor becomes:

$$\theta_{ik} = \delta_{ik}$$

The log hazard ratios γ_{ik} which measure the treatment effect of arm k relative to arm 1 in trial i with variance V_{ik} are defined in the normal likelihood:

$$\gamma_{ik} \sim Normal(\delta_{ik}, V_{ik})$$

3.2 Random effect model

In the random effects model,[1] the GLM formula additionally counts for between-trial heterogeneity ($\sigma^2 > 0$) and the cumulative adjustment for multi-arm trials $sw_{i,k}$

$$sw_{i,k} = \sum_{i=1}^{k-1} \frac{1}{k-1}\left(\delta_{i,k} - \left(d_{1,t_{ik}} - d_{1,t_{i1}}\right)\right)$$

Then, the GLM formula can be written as:

$$\delta_{i,k} \mid \delta_{i,2}, \dots, \delta_{i,(k-1)} \sim Normal(d_{1,t_{ik}} - d_{1,t_{i1}} + sw_{i,k}, \frac{k}{2(k-1)}\sigma^2)$$

3.3 Meta-regression model

In the meta-regression model,[2] the interaction treatment effect per unit increases in the covariate value in comparisons of treatment 2, 3, …, S to treatment 1. Thus, the GLM becomes:

$$\theta_{ik} = \mu_i + \delta_{ik} + \beta_{t_{i1}t_{ik}} x_i = \mu_i + \delta_{ik} + (\beta_{1,t_{ik}} - \beta_{1,t_{i1}}) x_i$$

The trial-level subgroup indicator, x_i, is defined:

$$x_i = \begin{cases} 0\ if\ study\ i\ is\ a\ primary\ study \\ 1\ if\ sudy\ i\ is\ a\ secondary\ study \end{cases}$$

3.4 Model parameters

D_{res}, the residual deviance,[3] is equal to the deviance for a given model, D_{model}, minus the deviance for a saturated model, D_{sat}, which estimates how good the model fit:

$$D_{res} = D_{model} - D_{sat}$$

$\overline{D}_{model}$, posterior mean residual deviances,[3] is obtained after computing D_{res} at each iteration of the MCMC simulation.

$\overline{D}_{model}$, posterior mean deviances,[3] is defined as -2 times the log-likelihood, $Loglik_{model}$, which likelihood function measures how 'likely' observed data are given a particular model, for a given model, thus estimates how far the model predictions deviate from the observed data:

$$\overline{D}_{model} = -2Loglik_{model}$$

p_D, the effective number of parameters,[3] is calculated by the posterior mean deviance for a given model, $\overline{D}_{model}$, minus the deviance calculated at some 'plug-in' estimate for the parameters, $\hat{\theta}$:

$$p_D = \overline{D}_{model} - D(\hat{\theta})$$

DIC, deviance information criterion,[3] is equal to the posterior mean deviance, $\overline{D}_{model}$, plus the effective number of parameters, p_D. The lower DIC value suggests a more parsimonious model:

$$DIC = \overline{D}_{model} + p_D$$

4. References

1. Dias S, Sutton AJ, Ades AE, Welton NJ. Evidence synthesis for decision making 2: a generalized linear modeling framework for pairwise and network meta-analysis of randomized controlled trials. *Med Decis Making* 2013;33: 607-17.

2. Dias S, Sutton AJ, Welton NJ, Ades AE. Evidence synthesis for decision making 3: heterogeneity--subgroups, meta-regression, bias, and bias-adjustment. *Med Decis Making* 2013;33: 618-40.

3. Dias S, Welton NJ, Sutton AJ, Ades AE. NICE DSU Technical support document 2: A generalised linear modelling framework for pairwise and network meta-analysis of randomised controlled trials. *London*, 2014.

4. van Valkenhoef G, Kuiper J. gemtc: Network Meta-Analysis Using Bayesian Methods. R package version 08-2. https://cran.r-project.org/web/packages/gemtc/gemtc.pdf, 2016.
