# Supplementary material for: Efficacy and Safety of Systemic Treatments Among Colorectal Cancer Patients: A Network Meta-Analysis of Randomized Controlled Trials
Source: Front Oncol. 2022 Feb 9;11:756214. doi: 10.3389/fonc.2021.756214 (PMC8864322; doi:10.3389/fonc.2021.756214)
Supplement: Supplementary file 2 [file DataSheet_2.pdf]

Supplementary Table S1. General characteristics of individual studies included in the network meta-analysis.

Study	Specific name/ Subgroup	Time	Country (Phase)	Line	Treatment arms		Sample size		Gender (M/F)		Age (years)	
					T1	T2	T1	T2	T1	T2	T1	T2
Lonardi 2020	PANDA (NCT02904031)	07/2016-04/2019	Italy (phase II)	1-line	FOLFOX/Pmab	5FU/LV/Pmab	92	93	61/92	57/93	77	77
Lipsyc-Sharf 2020 NCT02292758	ACCRU	07/2015-01/2018	US (phase II)	2-line	Cet/Iri/Bmab	Cet/Iri	19	17	10/9	10/7	58 (41-71)	54 (39-82)
Hu 2020	FOCULM (NCT02063529	02/2014-07/2019	China (phase II)	1-line	mFOLFOXIRI/Cet	mFOLFOXIRI	67	34				
Guo 2020	NCT02935764	10/2016-10/2019	China (phase III)	2-line	FOLFIRI	Iri	88	84				
Folprecht 2020	WT RAS	2014-2018	Germany (phase II)	1-line	FOLFOXIRI/Cmab	FOLFIRI/Cmab	28	26				
	MT RAS/BRAF				FOLFOXIRI	FOLFOXIRI/Bmab	18	16				
Xu 2019		06/2013-12/2017	China (phase II)	1-line	mFOLFOX6/Bmab	mFOLFOX6	121	120				
Pietrantonio 2019	VALENTINO (NCT02476045)	07/2015-07/2018	Italy (phase II)	1-line	5-FU/LV/Pmab	Pmab	117	112	79/38	74/38	63 (55-71)	62 (64-70)
Kienle 2019	SAKK 41/10 (NCT01718808)	01/2013-12/2014	Switzerland (phase II)	1-line	Cet	Cet/Cap	11	13	8/3	8/5	79 (71-89)	80 (73-86)
Uetake 2018	ATOM (NCT01836653)	05/2013-04/2016	Japan	1-line	mFOLFOX6/Cet	mFOLFOX6/Bmab	59	57			65	64
Shapiro 2018	WT-Quadruple	11/2012-06/2016	Australia (phase II)	2-line	Cet/Iri	Cet	25	21	18/7	13/8	65 (38-76)	67 (41-75)
Segelov 2016	MT-G13D	11/2012-12/2014	4 countries (phase II)	2-line	Cet/Iri	Cet	26	25	20/6	19/6	66 (48-85)	61 (49-82)
Qin 2018 NCT01228734	TAILOR	09/2010-01/2018	China (phase III)	1-line	Cet/FOLFOX4	FOLFOX4	193	200	127/66	139/61		
Nakayama 2018	CCOG-1201	06/2012-04/2016	Japan (phase II)	1-line	CAPIRI/Bmab	CAPOX/Bmab	53	54	32/21	36/18	69 (43-82)	67 (40-79)
Meropol 2018	ECOG ACRIN 4203	07/2005-04/2012	US (phase II)	1-line	IROX/Bmab	FOLFOX/Bmab	73	75	40/21	36/30	61 (37-85)	60 (31-79)
Maiello 2018	GOIM 2802		Italy (phase II)	1-line	FOLFOX4/Bmab	CAPOX/Bmab	45	87				
Hurwitz 2018 NCT01765582	STEAM	01/2013-03/2016	US (phase II)	1-line	cFOLFOXIRI/Bmab	FOLFOX/Bmab	93	95	51/42	59/36	56.0 (11.5)	57.9 (9.9)
					sFOLFOXIRI/Bmab		92		52/40		56.0 (10.5)	
Gomez 2018	VISNU-1 TTD (NCT01640405)	07/2012-11/2018	Spain (phase III)	1-line	FOLFOX/Bmab	FOLFOXIRI/Bmab	177	173	119/58	120/53	59	60.5
Geissler 2018	AIO-KRK-0109		phase II	1-line	mFOLFOXIRI/Pmab	FOLFOXIRI	63	33	41/22	24/9	56.5	58.2
Hou 2018		10/2010-10/2012	China	1-line	FOLFOXIRI/Bmab	FOLFOXIRI	58	58	26/32	24/34	57.4 (7.5)	57.9 (7.6)
Sugimoto 2017	WJOG6510G	12/2011-09/2014	Japan (phase II)	2-line	Pmab/Iri	Cet/Iri	59	61				
Schmoll 2017	CHARTA (NCT01321957)	07/2011-12/2014	Germany (phase II)	1-line	FOLFOX/Bmab	FOLFOXIRI/Bmab	120	121	157/84		61 (21-82)	
Carrato 2017	PLANET-TTD	05/2009-11/2012	Spain (phase II)	1-line	Pmab/FOLFOX4	Pmab/FOLFIRI	38	39	31/7	28/11	65 (32-79)	63 (37-83)
Yamazaki 2016	WJOG4407G	09/2008-01/2012	Japan (phase III)	1-line	Bmab/FOLFIRI	Bmab/mFOLFOX6	197	198	104/93	12/76	63 (33-75)	62 (26-75)

Study	Specific name/ Subgroup	Time	Country (Phase)	Line	Treatment arms		Sample size		Gender (M/F)		Age (years)	
					T1	T2	T1	T2	T1	T2	T1	T2
Shitara 2016	WJOG6210G (UMIN000005216)	04/2011-02/2014	Japan (phase II)	2-line	FOLFIRI/Pmab	FOLFIRI/Bmab	59	58	34/25	39/19	62 (31-82)	64 (26-78)
Ciardiello 2016	CAPRI-GOIM	02/2010-09/2014	Italy (phase II)	2-line	FOLFOX/Cet	FOLFOX	74	79	46/23	43/36	64 (35-79)	63 (40-80)
Aparicio 2016	FFCD 2001-02 (NCT00303771)	06/2003-05/2010	France (phase III)	1-line	LV5FU2	FOLFIRI	142	140	75/67	76/64	80 (75-90)	80 (75-92)
Tournigand 2015	GERCOR DREAM		3 countries (phase III)	1-line	mFOLFOX7/Bmab	XELOX2/Bmab	156	154				
Passardi 2015	ITACa (NCT01878422)	11/2007-03/2012	Italy (phase III)	1-line	FOLFOX4/Bmab	FOLFOX4	103	118	108/68	115/79	66 (34-83)	66 (33-82)
					FOLFIRI/Bmab	FOLFIRI	73	76				
Liu 2015	WT-KRAS	06/2010-05/2014	China (phase II)	2-line	Pmab/Bmab/FOLFIRI	FOLFIRI	30	35	18/12	22/13	59 (21-82)	62 (25-80)
	MU-KRAS						27	34	17/10	20/14	61 (22-79)	60 (29-85)
Hecht 2015	SPIRITT (NCT00418938)	11/2006-12/2010	US (phase II)	2-line	Pmab/FOLFIRI	Bmab/FOLFIRI	91	91	62/29	58/33	60 (27-84)	60 (25-80)
Gruenberger 2015	OLIVIA (NCT00778102)	10/2008-12/2011	4 countries (phase II)	1-line	Bmab/FOLFOXIRI	Bmab/mFOLFOX6	41	39	29/12	71/29	63 (32-77)	57 (28-80)
Cremolini 2015	TRIBE (NCT00719797)	07/2008-05/2011	Italy (phase III)	1-line	FOLFIRI/Bmab	FOLFOXIRI/Bmab	256	252	156/100	150/102	60 (53-67)	61 (52-68)
Xie 2014		12/2009-11/2013	China	2-line	FOLFIRI	Pmab/Bmab/FOLFIRI	155	137	98/57	81/56	58 (21-86)	61 (23-85)
Schwartzberg 2014	PEAK	04/2009-12/2011	6 countries (phase II)	1-line	Pmab/mFOLFOX6	Bmab/mFOLFOX6	142	143	86/56	96/47	63 (23-82)	61 (28-82)
Price 2014 NCT01001377	ASPECT	02/2010-07/2012	27 countries (phase III)	2-line	Pmab	Cet	499	500	315/184	318/182	61	60.5
Peeters 2014 Peeters 2010 NCT00339183	WT KRAS	06/2006-03/2008	Phase III	2-line	Pmab/FOLFIRI	FOLFIRI	303	294	188/115	191/103	60 (28-94)	61 (29-86)
	MT KRAS				Pmab/FOLFIRI	FOLFIRI	238	248	133/105	148/100	61 (29-83)	64 (29-86)
Heinemann 2014	FIRE-3 (NCT00433927)	01/2007-09/2012	2 countries (phase III)	1-line	FOLFIRI/Cet	FOLFIRI/Bmab	297	295	184/297	171/295	64 (38-79)	65 (27-76)
Folprecht 2014 Folprecht 2010	CELIM (NCT00153998)	12/2004-03/2008	2 countries (phase II)	1-line	FOLFOX6/Cet	FOLFIRI/Cet	56	55	36/20	35/20	65.1	62
Douillard 2014	WT KRAS	08/2006-02/2008	19 countries (phase III)	1-line	Pmab/FOLFOX4	FOLFOX4	325	331	217/108	204/127	62 (27, 85)	61 (24, 82)
	MT KRAS						221	219	145/76	128/91	63 (33, 83)	61 (27, 82)
Cao 2014		06/2010-05/2014	China (phase II)	2-line	Bmab/FOLFIRI	FOLFIRI	65	77	40/25	48/29	62 (30-79)	61 (24-81)
Seymour 2013	PICCOLO	12/2006-08/2010	UK	2-line	Iri	Pmab/Iri	230	230	158/72	160/70	63 (56-69)	64 (57-70)
Schmiegel 2013		07/2005-10/2006	Germany (phase II)	1-line	CAPOX/Bmab	mCAPIRI/Bmab	127	120	84/43	80/40	64 (27-84)	65 (30-82)
Personeni 2013	NCT01068132	04/2009-06/2012	Italy (phase II)	1-line	FOLFIRI	FOLFIRI/Cet	35	54				
Hong 2013		05/2006-04/2008	Korea (phase II)	1-line	Cap	CAPOX	40	40	23/17	22/18	71 (66-81)	72 (65-79)
Ducreux 2013	ACCORD 13/0503 (NCT00423696)	03/2006-01/2008	France (phase II)	1-line	Bmab/XELIRI	Bmab/FOLFIRI	72	73	64/36	48/52	61 (38-74)	61 (24-75)

Study	Specific name/ Subgroup	Time	Country (Phase)	Line	Treatment arms		Sample size		Gender (M/F)		Age (years)	
					T1	T2	T1	T2	T1	T2	T1	T2
Cunningham 2013	AVEX (NCT00484939)	07/2007-12/2010	10 countries (phase III)	1-line	Bmab/Cap	Cap	140	140	84/56	84/56	76 (70-87)	77 (70-87)
Tveit 2012	NORDIC-VII (NCT00145314)	05/2005-10/2007	4 countries (phase III)	1-line	Nordic FLOX	Cet/FLOX	185	194	100/85	120/74	61 (30-75)	61 (24-74)
Souglakos 2012	NCT00469443	06/2005-06/2008	Greece (phase II)	1-line	FOLFIRI/Bmab	CAPIRI/Bmab	167	166	104/63	109/57	66 (33-80)	67 (26-80)
Saltz 2012			phase III	1-line	mFOLFOX6/Bmab	LV5FU/Bmab/Cet	124	123	70/54	73/50	61 (32-87)	63 (35-87)
Pectasides 2012	ACTRN 12610000270011	01/2006-01/2008	phase III	1-line	XELIRI/Bmab	FOLFIRI/Bmab	143	142	79/64	92/50	66 (28-84)	66 (32-80)
Dotan 2012	NCT00321100	06/2006-05/2008	US (phase II)	1-line	CAPOX/Cet/Bmab	CAPOX/Cet	12	11	8/4	10/1	59 (42-78)	58 (42-74)
Van 2011			Phase III	1-line	FOLFIRI/Cet	FOLFIRI	599	599	369/230	356/243	61 (22-82)	61 (19-84)
Moosmann 2011	KRK-0104	09.2004-12/2006	Germany (phase II)	1-line	CAPIRI/Cet	CAPOX/Cet	89	88	63/26	63/25	63 (32-75)	62 (38-77)
Masi 2011 Falcone 2007	NCT01219920	11/2001-04/2005	Italy (phase III)	1-line	FOLFIRI	FOLFOXIRI	122	122	69/53	75/47	64 (21-75)	62 (27-75)
Guan 2011	ARTIST (NCT00642577)	07/2007-08/2008	China (phae III)	1-line	Bmab/mIFL	mIFL	139	64	36/28	70/69	53 (23-77)	50 (22-72)
Fischer 2011		07/2000-10/2004	Germany (phase III)	1-line	FUFIRI	mIROX	238	241	158/80	177/64	63 (32-79)	63 (21-79)
Ducreux 2011	ML16987	05/2003-08/2004	France (Phase III)	1-line	XELOX	FOLFOX6	156	150	100/56	90/60	66 (32-83)	64 (42-84)
Cassidy 2011 Cassidy 2008	NO16966	07/2003-05/2004	Phase II	1-line	FOLFOX4	XELOX	317	317	204/113	194/123	62 (24-83)	61 (24-84)
					FOLFOX4/Placebo	XELOX/Placebo	351	350	186/165	205/145	60 (26-83)	61 (18-83)
		02/2004-02/2005			FOLFOX4/Bmab	XELOX/Bmab	349	350	59/41	213/137	60 (19-82)	61 (18-86)
Vamvakas 2010 Souglakos 2006	HORG	10/2000-12/2004	FOLFIRI	1-line	FOLFIRI	FOLFOXIRI	146	137	82/61	76/61	66 (39-84)	66 (25-82)
Ocvirk 2010	CECOG	07/2005-07/2006	13 countries (phase II)	1-line	FOLFOX6/Cet	FOLFIRI/Cet	77	74	43/34	45/29	62	62.5
Tol 2009	CAIRO2 (NCT00208546)	06/2005-12/2006	Netherlands (phase III)	1-line	CAPOX/Bmab	CAPOX/Bmab/Cet	368	368	205/163	233/135	62 (27-83)	62 (33-80)
Moehler 2009		2001-2006	Germany (phase II)	1-line	CAPIRI	CAPIRI/Bmab	17	29	9/8	22/7	66 (55-81)	60 (37-80)
Bokemeyer 2009		06/2005-03/2006	Phase II	1-line	FOLFOX4	Cet/FOLFOX4	168	169	92/76	89/80	60 (30-82)	62 (24-82)
Aranda 2009		10/2001-19/2005	Spain	1-line	FOLFIRI	FUIRI	173	173	110/63	110/63	63 (29-75)	63 (28-75)
Hochster 2008	TREE-1	12/2002-11/2003	US	1-line	mFOLFOX6	CAPOX	49	48	57/43	65/35	62 (35-79)	63 (32-84)
					bFOL		50		62/38		62 (31-84)	
	TREE-2	11/2003-04/2004			mFOLFOX6/Bmab	CAPOX/Bmab	71	72	61/39	58/42	64 (31-83)	62 (32-82)
					bFOL/Bmab		70		49/51		57 (30-85)	
Sobrero 2008	EPIC	05/2003-02/2006	Phase III	2-line	Cet/Iri	Iri	648	650	405/243	411/239	61 (23-85)	62 (21-90)

Study	Specific name/ Subgroup	Time	Country (Phase)	Line	Treatment arms		Sample size		Gender (M/F)		Age (years)	
					T1	T2	T1	T2	T1	T2	T1	T2
Rothenberg 2008		07/2003-05/2005	19 countries (phase II)	2-line	FOLFOX4	XELOX	314	313	191/123	194/119	60 (26-83)	61 (26-81)
Heinemann 2008	AIO	-	German	1-line	Cet/XELIRI	Cet/XELOX	93	92	-	-	63	62
Haller 2008		01/2001-04/2004	7 countries (phase III)	2-line	IROX	Iri	317	310	189/128	189/121	62 (25-88)	63 (28-85)
Borner 2008		06/2004-10/2005	Phase II	1-line	CAPOX	CAPOX/Cet	37	37	21/16	23/14	63 (47-80)	60 (37-81)
Saltz 2007	BOND-2	-	Phase II	2-line	Cet/Bmab/Iri	Cet/Bmab	43	40	26/17	26/14	64 (43-86)	56 (24-80)
Porschen 2007	AIO		2 countries (phase III)	1-line	CAPOX	FUFOX	241	233	150/91	146/87	66 (32-81)	64 (34-86)
Hospers 2006			Netherlands (phase III)	1-line	LV5FU	LV5FU+Oxa	151	151	88/63	100/51	62 (28-84)	62 (41-80)
Goldberg 2006		04/2001-04/2002	5 NCIs	1-line	FOLFOX4	rIFL	154	151	90/64	97/54	58 (19-83)	60 (27-83)
Polikoff 2005	EXPLORE	03/2003-10/2004	Phase III	2-line	Cet/FOLFOX4	FOLFOX4	43	47	49/41		59	
NCT00252564		09/2005-07/2007	US (phase III)	1-line	FOLFOX/Bmab	LV5FU/Cet/Bmab	124	123	70/54	73/50	61 (12)	63 (11)
Kalofonos 2005		01/1999-02/2002	Phase II	1-line	LV5FU/Iri	LV5FU+Oxa	147	148	90/57	92/56	66 (28-78)	65 (32-86)
Colucci 2005		03/1999-11/2002	Italy (phase III)	1-line	FOLFIRI	FOLFOX4	178	182	93/85	109/73	62 (32-75)	62 (31-75)
Comella 2005		01/2001-06/2003	Italy (phase III)	1-line	IRIFAFU	LV5FU+Oxa	136	140	72/64	81/59	62 (38-80)	62 (37-79)
Tournigand 2004	GERCOR	12/1997-09/1999	3 countries (phase III)	1-line	FOLFIRI	FOLFOX6	109	111	62/47	80/31	61 (29-75)	65 (40-75)
				2-line	FOLFOX6	FOLFIRI	81	69				
Hurwitz 2004		09/2000-05/2002	3 countries (phase III)	1-line	IFL/Bmab	IFL/Placebo	402	411	59/41	60/40	59.5	59.2
Goldberg 2004		05/1999-04/2001	5 NCIs	1-line	FOLFOX4	IFL	267	264	157/110	172/92	61 (27-88)	61 (28-88)
					IROX		264		161/103		61 (29-84)	
Cunningham 2004		07/2001-05/2002	11 countries	2-line	Cet/Iri	Cet	218	111	143/75	63/48	59 (26-82)	58 (39-84)
Rothenberg 2003		11/2000-09/2001	US (phase III)	2-line	LV5FU2	FOLFOX4	151	152	82/69	87/65	60 (21-80)	59 (22-88)
					Oxa		156		95/61		61 (27-79)	
Rougier 2002		06/1997-08/1998	Phase II	2-line	Iri/LV5FU2	IROX	35	33	23/12	14/19	66 (39-76)	60 (31-75)
					Oxa/LV5FU2		33		25/8		65 (39-75)	
Maiello 2000	GOIM 9706	11/1997-01/1999	Italy (phase II)	1-line	LV5FU2	LV5FU2/Iri	34	68	22/12	35/33	61 (42-75)	60 (33-75)
De Gramont 2000		08/1995-07/1997	9 countries (phase III)	1-line	LV5FU2	LV5FU2/Oxa	210	210	122/88	127/83	63 (22-76)	63 (20-76)
NCT02337946	SAPPHIRE	10/2014-03/2017	Japan (phase II)	2-line	mFOLFOX6/Pmab	5-FU/LV/Pmab	164		109/55		66.6 (10.3)	

Study	Specific name/ Subgroup	Time	Country (Phase)	Line	Treatment arms		Sample size		Gender (M/F)		Age (years)	
					T1	T2	T1	T2	T1	T2	T1	T2
NCT01374425	MAVERICC	08/2011-05/2015	7 countries (phase II)	1-line	Bmab/mFOLFOX6	Bmab/FOLFIRI	188	188	122/66	117/71	59.2 (10.9)	60.7 (10.7)
NCT01131078		06/2005-11/2012	Italy (phase II)	1-line	Bmab/CAPIRI	Bmab/Cap1250	101	102	43/58	49/53	61.6 (9.35)	61.5 (10.2)
						Bmab/Cap650		103		40/63		61.0 (10.1)
NCT00778830	APEC	02/2009-04/2014	Singapore (phase II)	1-line	Cet/FOLFIRI	Cet/FOLFOX	101	188	66/35	119/69		
NCT00349336		08/2006-11/2008	3 countries (phase III)	1-line	XELOX/Bmab	FOLFOX4/Bmab	32	32	19/13	15/17	55.9 (12.6)	57.9 (10.8)

Bmab, bevacizumab; Cap, capecitabine; Cet, cetuximab; Iri, irinotecan; Oxa, oxaliplatin; Pmab, panitumumab; CAPOX or XELOX, capecitabine/oxaliplatin; CAPIRI or XELIRI, capecitabine/irinotecan; FOL or FOLFOX or FUFOX, 5-fluorouracil/leucovorin/oxaliplatin; FOLFOFIRI, 5-fluorouracil/leucovorin/oxaliplatin/irinotecan; FUFIRI or FOLFIRI or IFL, 5-fluorouracil/leucovorin/irinotecan; FUIRI, 5-fluorouracil/irinotecan; IROX, irinotecan/oxaliplatin; LV5FU, leucovorin/5-fluorouracil; b, bolus; c, concurrently; m, modified; s, sequentially.

Supplementary Table S2. Summary findings of individual studies included in the network meta-analysis.

Study	Treatment arms		ORR		DCR		OS	PFS	AEs grade ≥3		SAEs	
	T1	T2	T1	T2	T1	T2			T1	T2	T1	T2
Lonardi 2020	LFOP	LFP	60/92	53/93								
Lipsyc-Sharf 2020	CIB	CI	7/19	2/17			0.41 (0.15-1.09)	0.64 (0.25-1.66)	9/19	6/17		
NCT02292758	CIB	CI			13/19	9/17					5/19	5/17
Hu 2020	LFOIC	LFOI	64/67	26/34								
Guo 2020	LFI	I	5/86	5/84	54/88	46/84						
Folprecht 2020	LFOIC	LFIC	21/26	24/28								
	LFOI	LFOIB	13/18	14/20								
Xu 2019	LFOB	LFO	66/121	44/120								
Pietrantonio 2019	CIB	CI	78/117	75/112	97/117	94/112	1.13 (0.71-1.81)	1.51 (1.11-2.07)				
Kienle 2019	C	XC	1/11	4/13	6/11	9/13						
Uetake 2018	LFOC	LFOB	51/59	39/57				0.80 (0.51-1.26)				
Shapiro 2018	CI	C	9/24	2/20	20/24	16/20	0.66 (0.35-1.23)	0.39 (0.20-0.78)	12/24	5/20	10/24	3/20
Segelov 2016	CI	C	2/23	0/24	18/23	14/24	0.95 (0.53-1.68)	0.74 (0.41-1.31)	16/27	11/25	10/27	5/25
Qin 2018	LFOC	LFO	128/193	81/200			0.76 (0.61-0.95)	0.63 (0.50-0.79)				
NCT01228734	LFOC	LFO									42/194	27/199
Nakayama 2018	XIB	XOB	29/53	30/54	49/53	51/54	1.19 (0.72-1.96)	1.13 (0.75-1.70)				
Meropol 2018	IOB	LFOB	20/61	25/66	45/61	61/66	1.30 (0.90-1.90)	1.10 (0.80-1.60)				
Maiello 2018	LFOB	XOB	25/45	42/87			1.21 (0.77-1.92)	0.96 (0.65-1.41)				
Hurwitz 2018	LFOIB	LFOB	134/185	59/95				0.68 (0.50-0.92)				
NCT01765582	LFOIB	LFOB									81/181	43/90
Gomez 2018	LFOB	LFOIB							110/177	132/173		
Geissler 2018	LFOIP	LFOI	54/63	18/33	61/63	26/33		1.12 (0.69-1.75)			21/63	4/33
Hou 2018	LFOIB	LFOI	41/58	29/58	51/58	45/58						
Sugimoto 2017	IP	CI	13/59	16/61	44/59	50/61	0.68 (0.46-1.02)	0.68 (0.47-0.99)				
Schmoll 2017	LFOB	LFOIB	72/120	85/121	102/120	110/121		0.80 (0.61-1.04)				
Carrato 2017	LFOP	LFIP	28/38	26/39			1.00 (0.60-1.80)	0.90 (0.60-1.50)	32/38	30/39	13/34	15/39

Study	Treatment arms		ORR		DCR		OS	PFS	AEs grade ≥3		SAEs	
	T1	T2	T1	T2	T1	T2			T1	T2	T1	T2
Yamazaki 2016	LFIB	LFOB	118/185	112/180	170/185	166/180	0.99 (0.79-1.25)	0.91 (0.72-1.13)				
Shitara 2016	LFIP	LFIB	24/52	3/53	41/52	51/53			54/61	40/60		
Ciardiello 2016	LFOC	LFO	16/74	10/79	48/74	47/79	0.57 (0.32-1.02)	0.81 (0.58-1.12)				
Aparicio 2016	LF	LFI	28/133	61/132	87/133	104/132	0.96 (0.75-1.24)	0.84 (0.66-1.07)	71/136	103/135		
Tournigand 2015	LFOB	XOB					1.24 (0.98-1.59)	0.98 (0.74-1.31)	43/156	58/154		
Passardi 2015	LFOB	LFO	58/103	59/118								
	LFIB	LFI	31/73	38/76								
Liu 2015	LFIBP	LFI	14/30	9/35	19/30	12/35	0.79 (0.47-0.89)	0.45 (0.22-0.64)	28/30	21/35		
	LFIBP	LFI	12/27	10/34	20/27	18/34	0.44 (0.19-0.61)	0.65 (0.37-0.88)	25/27	19/34		
Hecht 2015	LFIP	LFIB	28/87	16/83	63/87	66/83	1.06 (0.75-1.49)	1.01 (0.68-1.50)	77/91	65/91	42/91	30/91
Gruenberger 2015	LFOIB	LFOB	33/41	24/39			0.35 (0.15-0.80)	0.43 (0.26-0.72)	38/40	31/37	24/40	24/37
Cremolini 2015	LFIB	LFOIB	139/257	164/252			0.80 (0.65-0.98)	0.77 (0.65-0.93)				
Xie 2014	LFI	LFIBP	47/155	55/137	81/155	85/137			124/155	80/137		
Schwartzberg 2014	LFOP	LFOB	82/142	76/143	128/142	122/143	0.62 (0.44-0.89)	0.87 (0.65-1.17)	126/139	115/139	61/139	53/139
Price 2014	P	C	107/486	96/485	333/486	332/485	0.97 (0.84-1.11)	1.00 (0.88-1.14)	246/496	236/503		
NCT01001377	P	C									169/503	151/496
Peeters 2014	LFIP	LFI	107/297	28/286	221/297	185/286	0.92 (0.78-1.10)	0.82 (0.69-0.97)				
	LFIP	LFI	31/232	35/237	159/232	148/237	0.93 (0.77-1.13)	0.94 (0.78-1.14)				
Peeters 2010	LFIP	LFI							219/302	152/294		
	LFIP	LFI							151/237	123/246		
NCT00339183	LFIP	LFI									232/587	175/594
Heinemann 2014	LFIC	LFIB	184/297	171/295	237/297	256/295	0.77 (0.62-0.96)	1.06 (0.88-1.26)	211/297	188/295		
Folprecht 2014	LFOC	LFIC					1.18 (0.79-1.74)	1.03 (0.66-1.61)				
Folprecht 2010	LFOC	LFIC	36/53	30/53	51/53	46/53			38/54	41/55		
Douillard 2014	LFOP	LFO	181/317	154/324	280/317	271/324	0.88 (0.73-1.06)	0.80 (0.67-0.95)			262/585	198/584
	LFOP	LFO	86/215	86/211	166/215	176/211	1.17 (0.95-1.45)	1.27 (1.04-1.55)				
Cao 2014	LFIB	LFI	31/65	22/77	56/65	51/77			41/65	58/77		
Seymour 2013	I	IP	27/230	79/230	118/230	135/230	1.01 (0.83-1.23)	0.78 (0.64-0.95)	87/218	129/219		

Study	Treatment arms		ORR		DCR		OS	PFS	AEs grade ≥3		SAEs	
	T1	T2	T1	T2	T1	T2			T1	T2	T1	T2
Schmiegel 2013	XOB	XIB	67/127	67/120	103/127	101/120	0.90 (0.68-1.19)	0.93 (0.82-1.07)				
Personeni 2013	LFI	LFIC	18/35	28/54								
Hong 2013	X	XO	9/40	14/40	32/40	36/40	0.67 (0.41-1.10)	1.33 (0.74-2.37)				
Ducreux 2013	XIB	LFIB	45/72	46/73	60/72	63/73					37/72	29/73
Cunningham 2013	XB	X	27/140	14/140	104/140	81/140	0.79 (0.57-1.09)	0.53 (0.41-0.69)	81/134	60/136	40/134	42/136
Tveit 2012	LF	LFC	31/76	46/94			1.06 (0.83-1.35)	0.89 (0.72-1.11)				
Souglakos 2012	LFIB	XIB	76/167	62/166	126/167	114/166	1.08 (0.94-1.24)	0.99 (0.90-1.09)	51/167	62/166		
Saltz 2012	LFOB	LFBC	61/117	49/119	102/117	99/119						
Pectasides 2012	XIB	LFIB	55/143	57/142	85/143	98/142	0.84 (0.59-1.21)	0.95 (0.68-1.32)				
Dotan 2012	XOBC	XOC	4/12	8/11	11/12	11/11						
Van 2011	LFIC	LFI	281/599	232/599	505/599	512/599	0.88 (0.77-1.00)	0.85 (0.73-1.00)	476/600	367/602		
Moosmann 2011	XIC	XOC	41/89	42/88	66/89	68/88						
Masi 2011	LFI	LFOI					0.74 (0.56-0.96)	0.59 (0.45-0.76)				
Falcone 2007	LFI	LFOI	41/122	66/122	74/122	87/122						
Guan 2011	LFIB	LFI	49/139	11/64	130/130	44/64	0.62 (0.41-0.95)	0.44 (0.31-0.63)				
Fischer 2011	LFI	IO	97/238	98/241	192/238	163/241	1.08 (0.88-1.23)	1.14 (0.94-1.37)				
Ducreux 2011	XO	LFO	39/156	46/150			1.02 (0.80-1.29)	1.00 (0.79-1.27)				
Cassidy 2011	LFO	XO					0.95 (0.84-1.07)		506/648	468/655		
	LFOB	XOB					0.95 (0.80-1.13)		289/342	266/353		
Cassidy 2008	LFO	XO						0.96 (0.81-1.13)				
	LFOB	XOB						1.01 (0.85-1.12)				
Vamvakas 2010	LFI	LFOI	49/146	59/137			1.08 (0.80-1.45)	1.15 (0.86-1.48)				
Souglakos 2006	LFI	LFOI			88/146	102/137						
Ocvirk 2010	LFOC	LFIC	33/77	33/74	64/77	57/74	0.98 (0.67-1.44)	1.06 (0.74-1.52)	48/77	37/74	21/77	21/74
Tol 2009	XOB	XOBC	184/368	194/368	346/368	348/368	1.15 (0.95-1.40)	1.22 (1.05-1.42)	269/368	301/368	268/368	299/368
Moehler 2009	XI	XIB	5/17	10/29	12/17	22/29			14/17	17/29		
Bokemeyer 2009	LFO	LFOC	60/168	77/169	136/168	144/169		0.93 (0.71-1.23)	117/168	129/170		
Aranda 2009	LFI	FI	98/173	88/173	142/173	137/173						

Study	Treatment arms		ORR		DCR		OS	PFS	AEs grade ≥3		SAEs	
	T1	T2	T1	T2	T1	T2			T1	T2	T1	T2
Hochster 2008	LFO	XO	20/49	13/48	32/49	32/48			37/49	35/48		
	LF		10/50		31/50				22/50			
	LFOB	XOB	37/71	33/72	65/71	55/72			46/71	42/72		
	LFB		27/70		53/70				42/70			
Sobrero 2008	CI	I	106/648	27/650	398/648	298/650	0.98 (0.85-1.14)	0.69 (0.62-0.78)	396/638	274/629	186/638	142/629
Rothenberg 2008	LFO	XO	57/314	63/313			1.03 (0.87-1.23)	0.97 (0.83-1.14)	262/308	198/311		
Heinemann 2008	XIC	XOC	39/74	42/68	62/74	66/68						
Haller 2008	IO	I	70/317	23/310	248/317	169/310			227/313	179/301	100/313	99/301
Borner 2008	XO	XOC	5/37	15/37	28/37	28/37						
Saltz 2007	CIB	CB	16/37	8/40								
Porschen 2007	XO	LFO	116/241	126/233	183/241	179/233		1.17 (0.96-1.43)				
Hospers 2006	LF	LFO	28/151	51/151	104/151	116/151			46/151	34/151		
Goldberg 2006	LFO	LFI	74/154	48/151			0.76 (0.60-0.97)	0.55 (0.43-0.70)				
Polikoff 2005	LFOC	LFO	9/43	4/42	29/43	30/42						
NCT00252564	LFOB	LFBC	61/117	49/119							40/118	49/121
Kalofonos 2005	LFI	LFO	49/147	45/142	91/147	82/142						
Colucci 2005	LFI	LFO	56/178	62/182	124/178	128/182	1.04 (0.80-1.37)					
Comella 2005	LFI	LFO	42/135	61/139	78/135	91/139			72/135	61/139		
Tournigand 2004	LFI	LFO	61/109	59/111	86/109	89/111			58/110	81/110	15/110	5/110
	LFO	LFI	12/81	3/69	51/81	24/69			40/82	30/68	3/81	4/69
Hurwitz 2004	LFIB	LFI	180/402	143/411					294/397	334/393	197/397	220/393
Goldberg 2004	LFO	LFI	120/267	82/264			0.66 (0.54-0.82)	0.74 (0.61-0.89)				
	IO	(LFI)	92/264				0.81 (0.66-1.00)	1.02 (0.85-1.23)				
	(LFO)	(IO)					0.83 (0.67-1.03)	0.72 (0.60-0.87)				
Cunningham 2004	CI	C	50/218	12/111	121/218	36/111	0.91 (0.68-1.21)	0.54 (0.42-0.71)	138/212	50/115		
Rothenberg 2003	LF	LFO	4/151	21/152	64/151	105/152			58/142	109/150		
	O		5/156		62/156				71/153			
Rougier 2002	LFI	IO	4/35	5/33	21/35	20/33						

Study	Treatment arms		ORR		DCR		OS	PFS	AEs grade ≥3		SAEs	
	T1	T2	T1	T2	T1	T2			T1	T2	T1	T2
	LFO		7/33		23/33							
Maiello 2000	LF	LFI	6/33	27/64	21/33	53/64						
De Gramont 2000	LF	LFO	46/210	105/210	153/210	172/210						
NCT02337946	LFOP	LFP	45/56	50/57			1.41 (0.69-2.88)	0.93 (0.60-1.43)	45/56	39/54	20/56	18/54
NCT01374425	LFOB	LFIB	115/188	123/188	175/188	171/188	0.76 (0.56-1.04)	0.79 (0.61-1.01)			79/185	87/183
NCT01131078	XIB	XB	47/91	63/186	83/91	154/186					38/100	42/201
NCT00778830	LFIC	LFOC	55/101	115/188							37/101	64/188
NCT00349336	XOB	LFOB									9/32	15/32

ORR, overall response rate; DCR, disease control rate; OS, overall survival; PFS, progression-free survival; AE, adverse event; SAE, serious adverse event; X, capecitabine; B, bevacizumab; C, cetuximab; F, 5-fluorouracil; I, irinotecan; L, leucovorin; O, oxaliplatin; P, panitumumab.

Supplementary Table S3. Identification of treatment arms included in the network meta-analysis.

Treatment	No. arms	No. subjects	ORR	DCR	OS	PFS	AEs grade ≥3	SAEs
X	2	180	Included	Included	Included	Included	N/c	Included
C	6	1168	Included	Included	N/c	N/c	N/c	N/c
I	4	1274	Included	Included	N/c	N/c	N/c	N/c
O	1	156	Included	Included	N/a	N/a	Included	N/a
P	3	1110	Included	Included	N/c	N/c	N/c	N/c
CB	1	40	Included	N/a	N/a	N/a	N/a	N/a
CI	6	995	Included	Included	N/c	N/c	N/c	N/c
CIB	2	81	Included	Included	N/c	N/c	N/c	N/c
IO	4	62	Included	Included	Included	Included	N/c	N/c
IOB	1	73	Included	Included	Included	Included	N/a	N/a
IP	2	289	Included	Included	N/c	N/c	N/c	N/a
FI	1	173	Included	Included	N/a	N/a	N/a	N/a
XB	2	345	Included	Included	Included	Included	N/c	Included
XC	1	13	Included	Included	N/a	N/a	N/a	N/a
XI	1	17	Included	Included	N/a	N/a	Included	N/a
**XIB**	7	684	Included	Included	Included	Included	Included	Included
XIC	2	182	Included	Included	N/a	N/a	N/a	N/a
XO	8	2169	Included	Included	Included	Included	Included	N/a
**XOB**	9	1594	Included	Included	Included	Included	Included	Included
XOC	4	228	Included	Included	N/a	N/a	N/a	N/a
**XOBC**	2	380	Included	Included	Included	Included	Included	Included
LF	7	923	Included	Included	Included	Included	Included	N/a
LFB	1	70	Included	Included	N/a	N/a	Included	N/a
LFC	1	194	Included	N/a	Included	Included	N/a	N/a
LFBC	2	246	Included	Included	N/a	N/a	N/a	Included
LFP	3	267	Included	N/a	Included	Included	Included	Included
**LFI**	31	5444	Included	Included	Included	Included	Included	Included
**LFIB**	13	2146	Included	Included	Included	Included	Included	Included
**LFIC**	8	1261	Included	Included	Included	Included	Included	Included
**LFIP**	8	1812	Included	Included	Included	Included	Included	Included
LFIBP	3	194	Included	Included	Included	Included	Included	N/a
**LFO**	26	5193	Included	Included	Included	Included	Included	Included
**LFOB**	20	2661	Included	Included	Included	Included	Included	Included
**LFOC**	10	1108	Included	Included	Included	Included	Included	Included
**LFOP**	6	874	Included	Included	Included	Included	Included	Included
LFOI	8	661	Included	Included	Included	Included	N/a	N/c
**LFOIB**	7	1033	Included	Included	Included	Included	Included	Included
LFOIC	2	93	Included	N/a	N/a	N/a	N/a	N/a

Treatment	No. arms	No. subjects	ORR	DCR	OS	PFS	AEs grade ≥3	SAEs
LFOIP	1	63	Included	Included	N/a	Included	N/a	N/c

ORR, overall response rate; DCR, disease control rate; OS, overall survival; PFS, progression-free survival; AEs, adverse events; SAEs, serious adverse events; X, capecitabine; B, bevacizumab; C, cetuximab; F, 5-fluorouracil; I, irinotecan; L, leucovorin; O, oxaliplatin; P, panitumumab; N/a, not available; N/c. not contributed. Bold fonts indicate treatments are available for all the outcomes.

Supplementary Table S4. Odds ratios and 95% credible intervals for pairwise estimates of overall response rate and disease control rate.

T1	T2	Overall response rate			Disease control rate		
		**Fixed effects**	**Random effects**	**Meta-regression**	**Fixed effects**	**Random effects**	**Meta-regression**
LFI	LFIB	**1.51 (1.27-1.78)**	**1.49 (1.11-1.96)**	1.40 (1.00-1.89)	**2.51 (1.80-3.41)**	**3.28 (1.87-5.56)**	**2.26 (1.29-3.95)**
LFI	LFIC	**1.62 (1.35-1.93)**	**1.80 (1.24-2.56)**	**1.75 (1.20-2.50)**	1.06 (0.80-1.37)	1.21 (0.63-2.11)	1.02 (0.58-1.69)
LFI	LFIP	**2.96 (2.21-3.88)**	**2.94 (1.91-4.36)**	**2.48 (1.39-4.08)**	**1.42 (1.10-1.80)**	1.46 (0.80-2.44)	0.83 (0.40-1.58)
LFI	LFIBP	**1.76 (1.15-2.60)**	**1.89 (1.04-3.21)**	1.47 (0.63-2.94)	**1.86 (1.23-2.72)**	2.23 (1.09-4.18)	1.12 (0.45-2.47)
LFI	LFO	**1.30 (1.12-1.50)**	**1.31 (1.04-1.65)**	1.27 (0.99-1.61)	**1.28 (1.04-1.55)**	1.41 (0.98-2.00)	1.20 (0.85-1.68)
LFI	LFOB	**1.53 (1.22-1.89)**	**1.54 (1.08-2.14)**	**1.47 (1.01-2.07)**	**2.25 (1.40-3.45)**	**2.97 (1.39-5.77)**	**2.17 (1.07-4.15)**
LFI	LFOC	**2.54 (1.98-3.22)**	**2.68 (1.81-3.86)**	**2.60 (1.74-3.76)**	**1.65 (1.11-2.38)**	1.90 (0.98-3.42)	1.60 (0.88-2.73)
LFI	LFOP	**1.74 (1.35-2.22)**	**1.86 (1.19-2.83)**	**1.77 (1.10-2.70)**	**1.57 (1.08-2.20)**	2.02 (0.98-3.83)	1.61 (0.86-2.90)
LFI	LFOI	**1.64 (1.19-2.22)**	1.60 (0.98-2.46)	1.58 (0.97-2.43)	**1.81 (1.25-2.55)**	1.94 (0.96-3.56)	1.84 (1.01-3.13)
LFI	LFOIB	**2.57 (1.92-3.38)**	**2.66 (1.67-4.05)**	**2.55 (1.58-3.91)**	**4.14 (1.93-7.96)**	**5.15 (1.66-12.6)**	**4.17 (1.50-9.70)**
LFI	LFOIC	**4.61 (1.46-11.4)**	**4.73 (1.32-12.6)**	**4.63 (1.28-12.3)**	-	-	-
LFI	LFOIP	**9.82 (3.01-24.9)**	**9.97 (2.37-28.7)**	**9.81 (2.35-28.2)**	**31.0 (3.40-136)**	**36.1 (2.60-176)**	**32.4 (2.82-157)**
LFI	LF	**0.39 (0.29-0.50)**	**0.39 (0.26-0.57)**	**0.38 (0.25-0.56)**	**0.62 (0.47-0.81)**	0.69 (0.41-1.09)	**0.60 (0.38-0.92)**
LFI	LFB	0.91 (0.45-1.63)	0.95 (0.37-2.04)	0.91 (0.34-1.95)	0.97 (0.37-2.11)	1.29 (0.30-3.74)	0.92 (0.25-2.53)
LFI	LFC	0.57 (0.28-1.05)	0.60 (0.21-1.36)	0.59 (0.21-1.33)	-	-	-
LFI	LFBC	0.99 (0.63-1.48)	1.03 (0.51-1.85)	0.98 (0.48-1.79)	1.74 (0.66-3.72)	2.52 (0.55-7.63)	1.78 (0.45-5.05)
LFI	LFP	1.60 (0.87-2.72)	1.88 (0.78-3.91)	1.79 (0.73-3.75)	-	-	-
LFI	CB	0.71 (0.15-2.09)	1.08 (0.13-4.10)	1.01 (0.13-3.83)	-	-	-
LFI	CI	1.64 (0.82-2.94)	2.07 (0.68-4.93)	1.93 (0.63-4.64)	**0.64 (0.40-0.98)**	0.97 (0.29-2.51)	0.58 (0.20-1.46)
LFI	CIB	1.97 (0.79-4.09)	2.85 (0.70-8.18)	2.68 (0.65-7.70)	0.73 (0.31-1.46)	1.27 (0.24-4.13)	0.73 (0.17-2.27)
LFI	IO	1.04 (0.82-1.30)	1.11 (0.72-1.65)	1.07 (0.69-1.60)	0.78 (0.55-1.06)	0.90 (0.46-1.63)	0.67 (0.35-1.19)
LFI	IOB	1.30 (0.56-2.60)	1.38 (0.44-3.26)	1.31 (0.42-3.13)	0.56 (0.13-1.48)	0.81 (0.12-2.76)	0.57 (0.10-1.87)
LFI	IP	1.38 (0.67-2.52)	1.72 (0.57-4.12)	1.60 (0.52-3.88)	**0.45 (0.26-0.74)**	0.66 (0.19-1.73)	0.40 (0.13-1.01)
LFI	FI	0.81 (0.52-1.21)	0.85 (0.39-1.61)	0.85 (0.39-1.62)	0.86 (0.48-1.42)	0.94 (0.31-2.24)	0.91 (0.35-1.95)
LFI	XB	0.71 (0.39-1.17)	0.76 (0.32-1.55)	0.73 (0.30-1.48)	0.86 (0.35-1.73)	1.12 (0.29-3.01)	0.80 (0.24-2.02)
LFI	XC	37.5 (0.26-113)	56.5 (0.24-142)	27.0 (0.22-136)	0.80 (0.08-3.36)	1.50 (0.07-7.78)	0.82 (0.05-4.12)
LFI	XI	1.25 (0.24-3.76)	1.33 (0.21-4.47)	1.26 (0.19-4.22)	1.66 (0.29-5.48)	2.31 (0.26-9.23)	1.58 (0.20-5.95)
LFI	XIB	1.30 (0.95-1.74)	1.33 (0.81-2.05)	1.25 (0.75-1.96)	**1.66 (1.06-2.50)**	2.11 (0.96-4.15)	1.49 (0.72-2.90)
LFI	XIC	**5.48 (1.54-14.7)**	**5.62 (1.22-17.1)**	**5.45 (1.17-16.6)**	0.93 (0.21-2.69)	1.10 (0.13-4.24)	0.88 (0.13-3.09)
LFI	XO	1.08 (0.82-1.39)	1.05 (0.67-1.56)	1.02 (0.64-1.52)	1.17 (0.77-1.72)	1.34 (0.61-2.62)	1.11 (0.55-2.06)
LFI	XOB	1.19 (0.79-1.70)	1.21 (0.68-2.01)	1.15 (0.63-1.93)	1.15 (0.58-2.05)	1.48 (0.50-3.47)	1.05 (0.40-2.38)
LFI	XOC	**6.53 (2.08-16.5)**	**6.62 (1.75-18.4)**	**6.41 (1.68-17.8)**	1.55 (0.43-4.05)	2.05 (0.35-6.91)	1.59 (0.33-4.99)
LFI	XOBC	1.33 (0.80-2.07)	1.36 (0.56-2.79)	1.30 (0.53-2.67)	1.28 (0.48-2.78)	1.65 (0.33-5.09)	1.18 (0.28-3.42)
LFI	X	**0.40 (0.19-0.76)**	**0.44 (0.16-0.96)**	**0.42 (0.15-0.93)**	**0.43 (0.16-0.89)**	0.57 (0.14-1.60)	0.42 (0.11-1.10)
LFI	C	0.58 (0.21-1.26)	0.70 (0.16-2.01)	0.66 (0.15-1.88)	**0.26 (0.13-0.47)**	0.45 (0.10-1.38)	0.26 (0.07-0.77)
LFI	I	**0.35 (0.20-0.55)**	**0.42 (0.18-0.84)**	**0.39 (0.17-0.78)**	**0.34 (0.22-0.49)**	**0.47 (0.20-0.95)**	**0.29 (0.13-0.60)**
LFI	O	**0.32 (0.09-0.73)**	**0.35 (0.09-0.88)**	**0.34 (0.08-0.87)**	**0.47 (0.29-0.72)**	0.55 (0.20-1.23)	**0.47 (0.20-0.96)**

T1	T2	Overall response rate			Disease control rate		
		Fixed effects	Random effects	Meta-regression	Fixed effects	Random effects	Meta-regression
LFI	P	0.67 (0.23-1.52)	0.85 (0.16-2.71)	0.80 (0.15-2.53)	**0.27 (0.13-0.50)**	0.50 (0.08-1.80)	**0.28 (0.06-0.94)**
LFIB	LFIC	1.08 (0.87-1.32)	1.22 (0.81-1.80)	1.27 (0.84-1.89)	**0.43 (0.30-0.59)**	**0.38 (0.18-0.69)**	**0.47 (0.24-0.80)**
LFIB	LFIP	**1.97 (1.44-2.65)**	**2.00 (1.26-3.05)**	**1.78 (1.05-2.84)**	**0.58 (0.40-0.82)**	**0.47 (0.23-0.82)**	**0.38 (0.19-0.65)**
LFIB	LFIBP	1.18 (0.74-1.79)	1.30 (0.66-2.33)	1.06 (0.47-2.10)	0.76 (0.44-1.22)	0.73 (0.28-1.56)	0.51 (0.21-1.07)
LFIB	LFO	0.87 (0.70-1.05)	0.90 (0.64-1.23)	0.93 (0.66-1.28)	**0.52 (0.36-0.73)**	**0.46 (0.24-0.79)**	**0.57 (0.31-0.93)**
LFIB	LFOB	1.02 (0.83-1.23)	1.04 (0.75-1.42)	1.06 (0.76-1.45)	0.91 (0.59-1.34)	0.92 (0.47-1.63)	0.98 (0.54-1.64)
LFIB	LFOC	**1.70 (1.29-2.20)**	**1.82 (1.17-2.74)**	**1.88 (1.20-2.86)**	0.67 (0.41-1.05)	0.61 (0.27-1.20)	0.75 (0.35-1.38)
LFIB	LFOP	1.16 (0.87-1.52)	1.26 (0.78-1.99)	1.28 (0.78-2.01)	**0.64 (0.40-0.97)**	0.65 (0.28-1.29)	0.75 (0.35-1.38)
LFIB	LFOI	1.10 (0.77-1.52)	1.09 (0.63-1.76)	1.15 (0.66-1.86)	0.74 (0.45-1.15)	0.63 (0.25-1.29)	0.87 (0.37-1.68)
LFIB	LFOIB	**1.71 (1.31-2.20)**	**1.80 (1.16-2.68)**	**1.83 (1.18-2.75)**	1.68 (0.77-3.24)	1.64 (0.51-3.98)	1.92 (0.66-4.38)
LFIB	LFOIC	3.08 (0.97-7.67)	3.22 (0.88-8.66)	3.36 (0.91-9.11)	-	-	-
LFIB	LFOIP	**6.56 (1.99-16.7)**	**6.79 (1.58-19.9)**	**7.13 (1.66-20.9)**	**12.7 (1.33-56.4)**	11.7 (0.76-58.0)	**15.4 (1.20-76.6)**
LFIB	LF	**0.26 (0.19-0.35)**	**0.27 (0.16-0.41)**	**0.28 (0.17-0.44)**	**0.26 (0.17-0.37)**	**0.22 (0.10-0.42)**	**0.29 (0.14-0.50)**
LFIB	LFB	0.61 (0.31-1.08)	0.64 (0.25-1.36)	0.65 (0.25-1.39)	**0.39 (0.15-0.82)**	0.40 (0.10-1.09)	0.41 (0.12-1.05)
LFIB	LFC	**0.38 (0.18-0.71)**	**0.41 (0.14-0.96)**	0.43 (0.14-1.00)	-	-	-
LFIB	LFBC	**0.66 (0.43-0.98)**	0.69 (0.35-1.24)	0.71 (0.35-1.27)	0.70 (0.27-1.48)	0.78 (0.18-2.26)	0.80 (0.22-2.10)
LFIB	LFP	1.07 (0.57-1.83)	1.28 (0.52-2.70)	1.30 (0.52-2.77)	-	-	-
LFIB	CB	0.47 (0.10-1.40)	0.74 (0.09-2.84)	0.74 (0.09-2.83)	-	-	-
LFIB	CI	1.10 (0.53-2.00)	1.41 (0.45-3.49)	1.41 (0.44-3.46)	**0.26 (0.15-0.44)**	**0.32 (0.08-0.87)**	**0.27 (0.08-0.66)**
LFIB	CIB	1.31 (0.52-2.78)	1.95 (0.46-5.71)	1.95 (0.46-5.73)	**0.30 (0.12-0.63)**	0.41 (0.07-1.40)	0.34 (0.07-1.02)
LFIB	IO	**0.69 (0.52-0.91)**	0.76 (0.45-1.21)	0.78 (0.46-1.26)	**0.32 (0.20-0.49)**	**0.29 (0.12-0.61)**	**0.31 (0.14-0.60)**
LFIB	IOB	0.87 (0.37-1.73)	0.93 (0.30-2.20)	0.94 (0.31-2.24)	**0.23 (0.05-0.59)**	**0.25 (0.04-0.83)**	**0.26 (0.05-0.79)**
LFIB	IP	0.92 (0.44-1.72)	1.18 (0.37-2.91)	1.17 (0.36-2.89)	**0.19 (0.10-0.33)**	**0.22 (0.05-0.60)**	**0.19 (0.06-0.46)**
LFIB	FI	**0.54 (0.33-0.83)**	0.58 (0.25-1.16)	0.62 (0.26-1.26)	**0.35 (0.18-0.62)**	**0.31 (0.08-0.79)**	0.44 (0.13-1.02)
LFIB	XB	**0.47 (0.27-0.77)**	0.52 (0.22-1.03)	0.52 (0.23-1.05)	**0.35 (0.15-0.67)**	**0.35 (0.10-0.89)**	**0.36 (0.12-0.85)**
LFIB	XC	24.1 (0.17-75.7)	38.6 (0.16-96.2)	19.3 (0.16-98.1)	0.33 (0.03-1.39)	0.49 (0.02-2.56)	0.38 (0.02-1.89)
LFIB	XI	0.83 (0.16-2.49)	0.89 (0.14-2.96)	0.90 (0.14-2.98)	0.66 (0.12-2.15)	0.71 (0.09-2.71)	0.70 (0.10-2.53)
LFIB	XIB	0.86 (0.66-1.11)	0.89 (0.59-1.29)	0.89 (0.59-1.30)	**0.66 (0.48-0.89)**	0.65 (0.36-1.07)	0.66 (0.40-1.03)
LFIB	XIC	**3.66 (1.02-9.82)**	3.82 (0.82-11.7)	3.94 (0.84-12.1)	0.38 (0.08-1.12)	0.35 (0.04-1.39)	0.41 (0.06-1.46)
LFIB	XO	**0.72 (0.53-0.96)**	0.71 (0.43-1.11)	0.74 (0.44-1.16)	**0.48 (0.28-0.76)**	**0.43 (0.17-0.91)**	0.52 (0.22-1.02)
LFIB	XOB	0.79 (0.54-1.11)	0.82 (0.47-1.31)	0.82 (0.48-1.34)	**0.46 (0.25-0.78)**	**0.46 (0.17-0.97)**	**0.47 (0.20-0.94)**
LFIB	XOC	**4.36 (1.38-11.0)**	**4.50 (1.18-12.6)**	**4.64 (1.20-13.0)**	0.63 (0.17-1.69)	0.66 (0.11-2.25)	0.74 (0.14-2.34)
LFIB	XOBC	0.88 (0.54-1.36)	0.92 (0.39-1.85)	0.93 (0.39-1.89)	0.51 (0.20-1.08)	0.51 (0.11-1.49)	0.53 (0.14-1.41)
LFIB	X	**0.27 (0.13-0.50)**	**0.30 (0.11-0.65)**	**0.30 (0.11-0.67)**	**0.17 (0.07-0.35)**	**0.18 (0.04-0.49)**	**0.19 (0.05-0.48)**
LFIB	C	**0.39 (0.14-0.85)**	0.48 (0.11-1.41)	0.48 (0.11-1.39)	**0.11 (0.05-0.20)**	**0.15 (0.03-0.47)**	**0.12 (0.03-0.35)**
LFIB	I	**0.23 (0.13-0.37)**	**0.29 (0.12-0.60)**	**0.28 (0.12-0.59)**	**0.14 (0.08-0.22)**	**0.15 (0.05-0.35)**	**0.13 (0.05-0.28)**
LFIB	O	**0.21 (0.06-0.49)**	**0.24 (0.06-0.61)**	**0.24 (0.06-0.65)**	**0.19 (0.11-0.32)**	**0.18 (0.05-0.43)**	**0.22 (0.08-0.48)**
LFIB	P	0.45 (0.15-1.03)	0.58 (0.10-1.89)	0.58 (0.11-1.87)	**0.11 (0.05-0.22)**	**0.16 (0.02-0.60)**	**0.13 (0.02-0.42)**
LFIC	LFIP	**1.84 (1.31-2.51)**	1.69 (0.96-2.75)	1.45 (0.75-2.52)	1.37 (0.95-1.91)	1.32 (0.56-2.64)	0.85 (0.38-1.71)
LFIC	LFIBP	1.10 (0.69-1.67)	1.09 (0.53-2.01)	0.86 (0.35-1.80)	**1.79 (1.08-2.81)**	2.04 (0.75-4.58)	1.15 (0.42-2.69)

T1	T2	Overall response rate			Disease control rate		
		Fixed effects	Random effects	Meta-regression	Fixed effects	Random effects	Meta-regression
LFIC	LFO	**0.80 (0.65-0.99)**	0.75 (0.50-1.08)	0.75 (0.50-1.07)	1.23 (0.89-1.66)	1.27 (0.64-2.30)	1.25 (0.70-2.10)
LFIC	LFOB	0.95 (0.73-1.21)	0.88 (0.55-1.33)	0.86 (0.53-1.31)	**2.15 (1.29-3.39)**	**2.65 (1.07-5.67)**	**2.23 (1.01-4.50)**
LFIC	LFOC	**1.58 (1.22-2.01)**	**1.51 (1.01-2.18)**	**1.51 (1.01-2.17)**	**1.58 (1.02-2.35)**	1.67 (0.80-3.15)	1.63 (0.85-2.88)
LFIC	LFOP	1.08 (0.80-1.43)	1.06 (0.61-1.75)	1.04 (0.59-1.70)	1.51 (0.96-2.25)	1.82 (0.72-3.93)	1.67 (0.76-3.31)
LFIC	LFOI	1.02 (0.71-1.44)	0.92 (0.50-1.53)	0.93 (0.50-1.56)	**1.74 (1.09-2.64)**	1.76 (0.66-3.85)	1.93 (0.83-3.81)
LFIC	LFOIB	1.60 (1.16-2.16)	1.52 (0.86-2.48)	1.50 (0.84-2.44)	**3.98 (1.78-7.82)**	**4.64 (1.27-12.3)**	**4.33 (1.39-10.7)**
LFIC	LFOIC	2.86 (0.91-7.09)	2.68 (0.75-7.10)	2.69 (0.75-7.15)	-	-	-
LFIC	LFOIP	**6.11 (1.85-15.6)**	**5.71 (1.28-16.9)**	**5.77 (1.31-17.0)**	**29.8 (3.17-132)**	**32.8 (2.05-166)**	**33.9 (2.69-170)**
LFIC	LF	**0.24 (0.17-0.33)**	**0.22 (0.13-0.36)**	**0.23 (0.13-0.36)**	0.60 (0.41-0.85)	0.62 (0.28-1.22)	0.63 (0.32-1.14)
LFIC	LFB	0.57 (0.28-1.03)	0.54 (0.20-1.20)	0.53 (0.19-1.17)	**0.93 (0.35-2.03)**	1.15 (0.24-3.49)	0.94 (0.24-2.64)
LFIC	LFC	0.35 (0.17-0.66)	0.34 (0.11-0.81)	0.35 (0.11-0.82)	-	-	-
LFIC	LFBC	**0.62 (0.38-0.94)**	0.59 (0.27-1.10)	0.57 (0.26-1.09)	1.67 (0.62-3.61)	2.25 (0.44-7.11)	1.82 (0.44-5.27)
LFIC	LFP	0.99 (0.53-1.71)	1.08 (0.42-2.32)	1.05 (0.40-2.28)	-	-	-
LFIC	CB	0.44 (0.09-1.31)	0.62 (0.07-2.39)	0.59 (0.07-2.29)	-	-	-
LFIC	CI	1.02 (0.50-1.86)	1.19 (0.36-2.94)	1.14 (0.35-2.82)	0.62 (0.35-1.00)	0.88 (0.22-2.54)	0.61 (0.18-1.60)
LFIC	CIB	1.22 (0.48-2.59)	1.64 (0.38-4.82)	1.58 (0.36-4.65)	0.70 (0.28-1.46)	1.16 (0.19-4.04)	0.76 (0.16-2.43)
LFIC	IO	**0.64 (0.48-0.85)**	0.64 (0.36-1.05)	0.63 (0.35-1.04)	0.75 (0.48-1.11)	0.82 (0.31-1.79)	0.70 (0.30-1.40)
LFIC	IOB	0.81 (0.34-1.63)	0.78 (0.24-1.91)	0.77 (0.23-1.88)	0.54 (0.13-1.43)	0.72 (0.10-2.56)	0.59 (0.10-1.94)
LFIC	IP	0.86 (0.41-1.60)	0.99 (0.30-2.45)	0.94 (0.28-2.36)	**0.44 (0.23-0.75)**	0.60 (0.14-1.74)	0.42 (0.12-1.10)
LFIC	FI	**0.50 (0.31-0.78)**	**0.49 (0.20-0.99)**	0.50 (0.20-1.02)	0.83 (0.43-1.44)	0.86 (0.22-2.30)	0.96 (0.30-2.28)
LFIC	XB	**0.44 (0.24-0.74)**	**0.44 (0.17-0.92)**	**0.43 (0.17-0.90)**	0.83 (0.33-1.67)	1.00 (0.24-2.83)	0.83 (0.23-2.14)
LFIC	XC	22.7 (0.16-70.5)	33.4 (0.13-81.6)	15.6 (0.12-80.1)	0.77 (0.07-3.25)	1.36 (0.06-7.29)	0.85 (0.05-4.33)
LFIC	XI	0.77 (0.15-2.34)	0.76 (0.11-2.57)	0.74 (0.11-2.50)	1.58 (0.28-5.26)	2.05 (0.21-8.39)	1.61 (0.20-6.14)
LFIC	XIB	0.81 (0.57-1.10)	0.76 (0.42-1.24)	0.73 (0.40-1.21)	1.59 (0.99-2.42)	1.88 (0.75-4.01)	1.52 (0.69-3.05)
LFIC	XIC	3.40 (0.94-9.19)	3.21 (0.66-9.93)	3.20 (0.65-9.92)	0.89 (0.20-2.63)	0.99 (0.11-3.98)	0.91 (0.13-3.28)
LFIC	XO	**0.67 (0.49-0.90)**	**0.60 (0.34-0.97)**	**0.60 (0.34-0.96)**	1.13 (0.69-1.76)	1.20 (0.44-2.69)	1.16 (0.49-2.36)
LFIC	XOB	0.74 (0.48-1.08)	0.69 (0.35-1.21)	0.67 (0.34-1.19)	1.10 (0.55-1.99)	1.31 (0.40-3.29)	1.08 (0.38-2.50)
LFIC	XOC	4.06 (1.27-10.3)	3.79 (0.95-10.8)	3.76 (0.94-10.7)	1.49 (0.41-3.96)	1.84 (0.28-6.55)	1.65 (0.31-5.34)
LFIC	XOBC	0.82 (0.49-1.30)	0.78 (0.30-1.65)	0.76 (0.29-1.62)	1.22 (0.45-2.69)	1.47 (0.26-4.72)	1.21 (0.28-3.55)
LFIC	X	**0.25 (0.11-0.47)**	**0.25 (0.09-0.57)**	**0.25 (0.08-0.56)**	**0.41 (0.15-0.87)**	0.51 (0.11-1.52)	0.43 (0.11-1.17)
LFIC	C	**0.36 (0.13-0.79)**	0.40 (0.09-1.19)	0.39 (0.08-1.13)	**0.25 (0.12-0.47)**	0.41 (0.08-1.35)	**0.27 (0.06-0.83)**
LFIC	I	**0.22 (0.12-0.35)**	**0.24 (0.10-0.51)**	**0.23 (0.09-0.48)**	**0.32 (0.19-0.51)**	0.42 (0.14-1.01)	**0.30 (0.11-0.67)**
LFIC	O	**0.20 (0.06-0.46)**	**0.20 (0.05-0.52)**	**0.20 (0.05-0.52)**	**0.45 (0.26-0.73)**	0.50 (0.15-1.24)	0.48 (0.18-1.08)
LFIC	P	**0.42 (0.14-0.96)**	0.49 (0.08-1.59)	0.47 (0.08-1.52)	**0.26 (0.12-0.50)**	0.46 (0.06-1.75)	0.29 (0.05-1.01)
LFIP	LFIBP	**0.61 (0.36-0.97)**	0.67 (0.32-1.28)	0.61 (0.28-1.18)	1.33 (0.81-2.07)	1.66 (0.63-3.64)	1.41 (0.62-2.89)
LFIP	LFO	**0.45 (0.32-0.60)**	**0.47 (0.29-0.71)**	**0.55 (0.31-0.91)**	0.91 (0.66-1.23)	1.04 (0.52-1.90)	1.61 (0.77-2.93)
LFIP	LFOB	**0.53 (0.37-0.73)**	**0.54 (0.32-0.86)**	0.63 (0.35-1.06)	1.61 (0.95-2.55)	2.16 (0.88-4.61)	2.85 (1.21-5.77)
LFIP	LFOC	0.88 (0.60-1.24)	0.95 (0.53-1.57)	1.12 (0.58-1.98)	1.18 (0.74-1.81)	1.40 (0.58-2.93)	2.14 (0.88-4.36)
LFIP	LFOP	**0.60 (0.41-0.84)**	0.66 (0.37-1.09)	0.76 (0.40-1.32)	1.12 (0.72-1.67)	1.49 (0.59-3.23)	2.14 (0.88-4.42)
LFIP	LFOI	**0.57 (0.37-0.84)**	**0.57 (0.29-0.99)**	0.68 (0.32-1.28)	1.29 (0.82-1.94)	1.44 (0.56-3.07)	2.50 (0.93-5.30)

T1	T2	Overall response rate			Disease control rate		
		Fixed effects	Random effects	Meta-regression	Fixed effects	Random effects	Meta-regression
LFIP	LFOIB	0.89 (0.59-1.28)	0.94 (0.51-1.59)	1.10 (0.55-1.95)	**2.96 (1.32-5.82)**	**3.78 (1.07-9.95)**	**5.55 (1.66-13.9)**
LFIP	LFOIC	1.59 (0.48-4.04)	1.68 (0.43-4.64)	2.01 (0.48-5.75)	-	-	-
LFIP	LFOIP	3.39 (1.00-8.80)	3.53 (0.77-10.5)	4.25 (0.89-13.0)	**22.2 (2.37-98.4)**	**26.8 (1.73-133)**	**43.9 (3.22-219)**
LFIP	LF	**0.13 (0.09-0.19)**	**0.14 (0.08-0.23)**	**0.17 (0.08-0.30)**	**0.45 (0.30-0.63)**	**0.51 (0.23-0.99)**	0.82 (0.35-1.58)
LFIP	LFB	**0.31 (0.15-0.59)**	**0.34 (0.12-0.76)**	**0.39 (0.13-0.90)**	0.69 (0.26-1.52)	0.94 (0.20-2.84)	1.20 (0.29-3.36)
LFIP	LFC	**0.20 (0.09-0.38)**	**0.21 (0.07-0.51)**	**0.25 (0.08-0.64)**	-	-	-
LFIP	LFBC	**0.34 (0.20-0.55)**	**0.36 (0.16-0.70)**	**0.42 (0.18-0.85)**	1.24 (0.46-2.70)	1.84 (0.37-5.81)	2.33 (0.54-6.75)
LFIP	LFP	**0.55 (0.28-0.98)**	0.66 (0.25-1.44)	0.77 (0.28-1.72)	-	-	-
LFIP	CB	**0.24 (0.05-0.74)**	0.39 (0.04-1.49)	0.43 (0.05-1.69)	-	-	-
LFIP	CI	0.57 (0.26-1.07)	0.73 (0.22-1.86)	0.83 (0.24-2.12)	**0.46 (0.26-0.74)**	0.72 (0.18-2.04)	0.76 (0.23-1.89)
LFIP	CIB	0.68 (0.26-1.47)	1.01 (0.23-3.03)	1.15 (0.25-3.45)	0.52 (0.21-1.08)	0.94 (0.16-3.27)	0.95 (0.20-2.90)
LFIP	IO	**0.36 (0.24-0.50)**	**0.39 (0.21-0.67)**	**0.46 (0.23-0.83)**	**0.55 (0.36-0.82)**	0.67 (0.27-1.43)	0.88 (0.37-1.75)
LFIP	IOB	**0.45 (0.18-0.93)**	0.49 (0.15-1.21)	0.56 (0.16-1.43)	0.40 (0.09-1.07)	0.59 (0.08-2.09)	0.75 (0.12-2.48)
LFIP	IP	**0.48 (0.22-0.91)**	0.61 (0.18-1.55)	0.69 (0.20-1.76)	**0.33 (0.17-0.55)**	0.49 (0.12-1.40)	0.52 (0.15-1.31)
LFIP	FI	**0.28 (0.16-0.45)**	**0.30 (0.12-0.62)**	**0.37 (0.14-0.82)**	0.62 (0.33-1.06)	0.70 (0.19-1.84)	1.24 (0.35-3.12)
LFIP	XB	**0.24 (0.13-0.42)**	**0.27 (0.10-0.58)**	**0.31 (0.12-0.69)**	0.61 (0.25-1.25)	0.82 (0.19-2.30)	1.06 (0.28-2.76)
LFIP	XC	12.5 (0.09-39.5)	20.8 (0.08-50.1)	11.4 (0.09-57.8)	0.57 (0.05-2.41)	1.11 (0.05-5.87)	1.06 (0.06-5.32)
LFIP	XI	0.43 (0.08-1.32)	0.47 (0.07-1.61)	0.54 (0.08-1.86)	1.18 (0.21-3.93)	1.68 (0.18-6.78)	2.05 (0.25-7.81)
LFIP	XIB	**0.45 (0.29-0.65)**	**0.47 (0.25-0.80)**	**0.54 (0.27-0.95)**	1.19 (0.73-1.84)	1.53 (0.63-3.23)	1.94 (0.85-3.86)
LFIP	XIC	1.89 (0.51-5.18)	1.99 (0.40-6.20)	2.35 (0.45-7.50)	0.67 (0.15-1.95)	0.82 (0.09-3.28)	1.18 (0.15-4.30)
LFIP	XO	**0.37 (0.25-0.53)**	**0.37 (0.20-0.63)**	**0.44 (0.22-0.78)**	0.84 (0.51-1.31)	0.99 (0.37-2.21)	1.49 (0.57-3.18)
LFIP	XOB	**0.41 (0.25-0.63)**	**0.43 (0.21-0.77)**	**0.49 (0.23-0.93)**	0.82 (0.40-1.49)	1.07 (0.33-2.67)	1.37 (0.47-3.20)
LFIP	XOC	2.25 (0.68-5.82)	2.34 (0.57-6.74)	2.76 (0.64-8.09)	1.11 (0.30-2.95)	1.51 (0.23-5.39)	2.12 (0.37-6.99)
LFIP	XOBC	**0.46 (0.26-0.75)**	0.48 (0.18-1.04)	0.56 (0.20-1.25)	0.91 (0.33-2.01)	1.20 (0.22-3.86)	1.54 (0.34-4.53)
LFIP	X	**0.14 (0.06-0.27)**	**0.15 (0.05-0.36)**	**0.18 (0.06-0.43)**	**0.30 (0.11-0.65)**	0.41 (0.09-1.24)	0.55 (0.13-1.52)
LFIP	C	**0.20 (0.07-0.45)**	**0.25 (0.05-0.75)**	**0.28 (0.06-0.84)**	**0.19 (0.09-0.35)**	0.33 (0.06-1.10)	0.34 (0.08-0.98)
LFIP	I	**0.12 (0.06-0.20)**	**0.15 (0.06-0.32)**	**0.17 (0.06-0.37)**	**0.24 (0.15-0.37)**	**0.35 (0.12-0.81)**	**0.37 (0.15-0.79)**
LFIP	O	**0.11 (0.03-0.26)**	**0.12 (0.03-0.33)**	**0.15 (0.03-0.40)**	**0.34 (0.20-0.54)**	**0.41 (0.12-1.01)**	**0.63 (0.20-1.45)**
LFIP	P	**0.23 (0.08-0.54)**	**0.30 (0.05-0.99)**	0.34 (0.06-1.13)	**0.19 (0.09-0.37)**	0.37 (0.05-1.42)	0.37 (0.07-1.19)
LFIBP	LFO	0.77 (0.48-1.15)	0.75 (0.39-1.31)	1.00 (0.43-1.99)	0.71 (0.45-1.08)	0.71 (0.31-1.40)	1.27 (0.50-2.58)
LFIBP	LFOB	0.90 (0.55-1.39)	0.88 (0.43-1.61)	1.15 (0.48-2.33)	1.26 (0.66-2.19)	1.49 (0.50-3.54)	2.26 (0.77-5.14)
LFIBP	LFOC	1.51 (0.91-2.35)	1.53 (0.73-2.84)	2.04 (0.82-4.27)	0.92 (0.51-1.54)	0.96 (0.34-2.14)	1.69 (0.57-3.76)
LFIBP	LFOP	1.03 (0.62-1.61)	1.07 (0.49-2.03)	1.38 (0.55-2.91)	0.88 (0.50-1.43)	1.02 (0.35-2.36)	1.69 (0.57-3.82)
LFIBP	LFOI	0.97 (0.56-1.57)	0.92 (0.41-1.76)	1.25 (0.47-2.71)	1.01 (0.57-1.66)	0.98 (0.34-2.22)	1.97 (0.61-4.57)
LFIBP	LFOIB	1.52 (0.90-2.42)	1.53 (0.69-2.90)	2.00 (0.78-4.20)	2.32 (0.95-4.82)	2.60 (0.64-7.28)	4.39 (1.11-11.8)
LFIBP	LFOIC	2.73 (0.78-7.12)	2.71 (0.63-7.87)	3.65 (0.75-11.3)	-	-	-
LFIBP	LFOIP	**5.82 (1.62-15.5)**	**5.71 (1.14-17.7)**	**7.75 (1.39-25.5)**	**17.3 (1.77-77.4)**	**18.2 (1.09-91.3)**	**34.8 (2.28-176)**
LFIBP	LF	**0.23 (0.14-0.36)**	**0.22 (0.11-0.42)**	**0.30 (0.12-0.64)**	**0.35 (0.21-0.55)**	**0.35 (0.14-0.72)**	0.64 (0.23-1.38)
LFIBP	LFB	0.54 (0.23-1.06)	0.55 (0.17-1.32)	0.71 (0.20-1.84)	0.54 (0.19-1.26)	0.65 (0.12-2.08)	0.95 (0.20-2.85)
LFIBP	LFC	**0.34 (0.14-0.68)**	**0.34 (0.10-0.87)**	0.46 (0.12-1.26)	-	-	-

T1	T2	Overall response rate			Disease control rate		
		**Fixed effects**	**Random effects**	**Meta-regression**	**Fixed effects**	**Random effects**	**Meta-regression**
LFIBP	LFBC	0.59 (0.31-1.01)	0.59 (0.23-1.26)	0.77 (0.26-1.78)	0.97 (0.33-2.23)	1.27 (0.22-4.21)	1.85 (0.36-5.71)
LFIBP	LFP	0.95 (0.44-1.79)	1.08 (0.36-2.55)	1.40 (0.42-3.56)	-	-	-
LFIBP	CB	0.42 (0.08-1.29)	0.62 (0.07-2.48)	0.78 (0.08-3.17)	-	-	-
LFIBP	CI	0.97 (0.42-1.92)	1.19 (0.32-3.16)	1.50 (0.38-4.14)	**0.36 (0.19-0.62)**	0.49 (0.11-1.41)	0.59 (0.16-1.52)
LFIBP	CIB	1.16 (0.41-2.62)	1.64 (0.34-5.08)	2.08 (0.41-6.66)	**0.41 (0.16-0.88)**	0.64 (0.10-2.23)	0.74 (0.14-2.31)
LFIBP	IO	**0.61 (0.37-0.95)**	0.64 (0.30-1.20)	0.84 (0.33-1.76)	**0.43 (0.25-0.70)**	0.45 (0.16-1.02)	0.69 (0.25-1.48)
LFIBP	IOB	0.77 (0.29-1.67)	0.79 (0.21-2.09)	1.03 (0.25-2.88)	**0.31 (0.07-0.87)**	0.41 (0.05-1.50)	0.60 (0.08-2.06)
LFIBP	IP	0.82 (0.35-1.64)	0.99 (0.27-2.63)	1.24 (0.31-3.43)	**0.25 (0.12-0.46)**	**0.33 (0.07-0.97)**	0.41 (0.11-1.05)
LFIBP	FI	0.48 (0.25-0.82)	0.48 (0.17-1.07)	0.67 (0.20-1.66)	**0.48 (0.23-0.88)**	0.47 (0.12-1.29)	0.98 (0.23-2.63)
LFIBP	XB	0.42 (0.20-0.78)	0.44 (0.15-1.02)	0.57 (0.17-1.41)	0.48 (0.18-1.04)	0.56 (0.12-1.69)	0.84 (0.19-2.35)
LFIBP	XC	22.2 (0.15-67.0)	34.3 (0.12-80.0)	20.5 (0.15-104)	0.45 (0.04-1.90)	0.75 (0.03-4.01)	0.82 (0.04-4.13)
LFIBP	XI	0.74 (0.13-2.32)	0.76 (0.10-2.70)	0.98 (0.12-3.58)	0.93 (0.15-3.17)	1.16 (0.11-4.93)	1.63 (0.17-6.49)
LFIBP	XIB	0.77 (0.45-1.23)	0.76 (0.34-1.46)	0.98 (0.38-2.06)	0.93 (0.50-1.60)	1.07 (0.35-2.54)	1.54 (0.53-3.49)
LFIBP	XIC	3.24 (0.82-9.11)	3.22 (0.59-10.5)	4.28 (0.70-14.6)	0.52 (0.11-1.56)	0.56 (0.06-2.27)	0.93 (0.11-3.51)
LFIBP	XO	0.64 (0.38-1.00)	0.60 (0.28-1.13)	0.80 (0.31-1.68)	0.66 (0.36-1.11)	0.67 (0.22-1.60)	1.18 (0.37-2.72)
LFIBP	XOB	0.70 (0.38-1.17)	0.69 (0.29-1.40)	0.90 (0.33-1.98)	0.64 (0.28-1.26)	0.74 (0.19-2.02)	1.09 (0.30-2.81)
LFIBP	XOC	**3.87 (1.11-10.2)**	3.80 (0.84-11.43)	5.04 (0.99-15.9)	0.87 (0.22-2.37)	1.03 (0.14-3.76)	1.68 (0.26-5.75)
LFIBP	XOBC	0.78 (0.40-1.39)	0.78 (0.26-1.82)	1.02 (0.30-2.55)	0.72 (0.24-1.65)	0.83 (0.13-2.80)	1.22 (0.23-3.79)
LFIBP	X	**0.24 (0.10-0.49)**	**0.25 (0.07-0.62)**	**0.33 (0.09-0.87)**	**0.24 (0.08-0.54)**	**0.28 (0.05-0.90)**	0.43 (0.09-1.29)
LFIBP	C	**0.34 (0.11-0.80)**	0.40 (0.08-1.25)	0.51 (0.10-1.61)	**0.15 (0.07-0.29)**	**0.23 (0.04-0.75)**	**0.26 (0.06-0.78)**
LFIBP	I	**0.20 (0.10-0.37)**	**0.24 (0.08-0.56)**	**0.30 (0.10-0.73)**	**0.19 (0.10-0.32)**	**0.23 (0.07-0.57)**	**0.29 (0.10-0.65)**
LFIBP	O	**0.19 (0.05-0.46)**	**0.20 (0.04-0.55)**	**0.26 (0.05-0.80)**	0.26 (0.14-0.46)	0.28 (0.08-0.72)	0.49 (0.14-1.22)
LFIBP	P	**0.40 (0.12-0.96)**	0.49 (0.08-1.66)	0.62 (0.10-2.15)	0.15 (0.06-0.30)	0.25 (0.03-0.96)	**0.28 (0.05-0.95)**
LFO	LFOB	1.18 (0.94-1.46)	1.18 (0.83-1.62)	1.16 (0.81-1.60)	**1.77 (1.09-2.75)**	2.14 (0.98-4.18)	1.83 (0.91-3.41)
LFO	LFOC	**1.97 (1.56-2.45)**	2.05 (1.43-2.87)	2.05 (1.42-2.88)	1.30 (0.90-1.81)	1.36 (0.74-2.29)	1.34 (0.79-2.13)
LFO	LFOP	**1.35 (1.08-1.66)**	1.42 (0.94-2.08)	1.39 (0.92-2.04)	1.23 (0.89-1.65)	1.44 (0.75-2.56)	1.34 (0.78-2.24)
LFO	LFOI	1.28 (0.89-1.77)	1.24 (0.72-1.96)	1.26 (0.73-2.01)	1.43 (0.94-2.10)	1.42 (0.63-2.75)	1.57 (0.78-2.83)
LFO	LFOIB	**2.00 (1.47-2.65)**	**2.04 (1.26-3.13)**	**2.02 (1.24-3.11)**	**3.27 (1.49-6.33)**	**3.74 (1.15-9.33)**	**3.54 (1.24-8.23)**
LFO	LFOIC	**3.58 (1.13-8.92)**	3.64 (1.00-9.72)	**3.68 (1.01-9.88)**	-	-	-
LFO	LFOIP	**7.63 (2.31-19.4)**	**7.69 (1.79-22.41)**	**7.80 (1.82-22.7)**	**24.5 (2.65-108)**	**26.4 (1.81-130)**	**27.7 (2.32-136)**
LFO	LF	**0.30 (0.23-0.38)**	**0.30 (0.20-0.42)**	**0.30 (0.20-0.43)**	**0.49 (0.38-0.61)**	**0.49 (0.31-0.73)**	**0.51 (0.34-0.73)**
LFO	LFB	0.71 (0.35-1.26)	0.73 (0.28-1.56)	0.72 (0.27-1.53)	0.77 (0.29-1.67)	0.93 (0.21-2.72)	0.77 (0.21-2.10)
LFO	LFC	**0.44 (0.21-0.81)**	0.46 (0.16-1.03)	0.46 (0.17-1.04)	-	-	-
LFO	LFBC	0.77 (0.49-1.15)	0.79 (0.39-1.41)	0.77 (0.38-1.40)	1.37 (0.52-2.94)	1.82 (0.39-5.53)	1.50 (0.38-4.18)
LFO	LFP	1.24 (0.68-2.07)	1.44 (0.61-2.93)	1.41 (0.59-2.89)	-	-	-
LFO	CB	0.55 (0.11-1.63)	0.83 (0.10-3.16)	0.80 (0.10-3.03)	-	-	-
LFO	CI	1.27 (0.63-2.30)	1.59 (0.52-3.84)	1.53 (0.50-3.70)	**0.51 (0.30-0.80)**	0.71 (0.20-1.88)	0.49 (0.16-1.22)
LFO	CIB	1.52 (0.61-3.19)	2.19 (0.53-6.33)	2.12 (0.51-6.12)	0.58 (0.24-1.18)	0.92 (0.17-3.05)	0.62 (0.14-1.89)
LFO	IO	0.80 (0.62-1.02)	0.85 (0.54-1.29)	0.85 (0.53-1.29)	**0.61 (0.41-0.88)**	0.66 (0.31-1.24)	0.57 (0.29-1.01)
LFO	IOB	1.01 (0.43-2.01)	1.05 (0.34-2.50)	1.03 (0.34-2.45)	0.44 (0.10-1.17)	0.58 (0.09-1.99)	0.48 (0.08-1.55)

T1	T2	Overall response rate			Disease control rate		
		Fixed effects	Random effects	Meta-regression	Fixed effects	Random effects	Meta-regression
LFO	IP	1.07 (0.52-1.97)	1.32 (0.43-3.18)	1.27 (0.41-3.08)	**0.36 (0.20-0.60)**	0.48 (0.13-1.28)	**0.34 (0.11-0.84)**
LFO	FI	**0.63 (0.39-0.96)**	0.65 (0.28-1.28)	0.67 (0.29-1.34)	0.68 (0.37-1.16)	0.69 (0.21-1.69)	0.78 (0.27-1.74)
LFO	XB	**0.55 (0.30-0.91)**	0.59 (0.25-1.19)	0.58 (0.24-1.17)	0.68 (0.28-1.37)	0.81 (0.21-2.15)	0.68 (0.21-1.67)
LFO	XC	27.4 (0.20-87.6)	42.9 (0.18-108)	21.1 (0.17-108)	0.63 (0.06-2.67)	1.09 (0.05-5.71)	0.69 (0.04-3.45)
LFO	XI	0.97 (0.19-2.93)	1.02 (0.16-3.43)	1.00 (0.15-3.33)	1.31 (0.23-4.34)	1.68 (0.18-6.72)	1.33 (0.17-4.96)
LFO	XIB	1.01 (0.72-1.36)	1.02 (0.61-1.59)	0.99 (0.59-1.55)	1.31 (0.81-2.02)	1.53 (0.67-3.07)	1.25 (0.60-2.40)
LFO	XIC	**4.24 (1.20-11.3)**	4.29 (0.95-12.9)	4.29 (0.93-13.0)	0.73 (0.17-2.09)	0.78 (0.10-2.95)	0.74 (0.11-2.52)
LFO	XO	0.84 (0.67-1.04)	0.80 (0.54-1.12)	0.80 (0.54-1.12)	0.92 (0.63-1.30)	0.95 (0.46-1.75)	0.93 (0.50-1.60)
LFO	XOB	0.92 (0.61-1.33)	0.93 (0.52-1.54)	0.91 (0.50-1.52)	0.91 (0.45-1.64)	1.07 (0.36-2.53)	0.89 (0.34-1.97)
LFO	XOC	**5.05 (1.62-12.7)**	**5.06 (1.36-13.9)**	**5.05 (1.36-13.8)**	1.22 (0.35-3.15)	1.46 (0.26-4.79)	1.33 (0.28-4.06)
LFO	XOBC	1.03 (0.62-1.61)	1.05 (0.43-2.13)	1.03 (0.42-2.10)	1.01 (0.37-2.21)	1.19 (0.23-3.69)	0.99 (0.24-2.83)
LFO	X	**0.31 (0.15-0.58)**	**0.33 (0.12-0.73)**	**0.33 (0.12-0.72)**	**0.34 (0.13-0.71)**	**0.41 (0.10-1.14)**	**0.35 (0.10-0.91)**
LFO	C	**0.45 (0.16-0.98)**	0.54 (0.12-1.56)	0.52 (0.12-1.49)	**0.21 (0.10-0.38)**	0.33 (0.07-1.02)	**0.22 (0.06-0.64)**
LFO	I	**0.27 (0.15-0.43)**	**0.32 (0.14-0.65)**	**0.31 (0.13-0.62)**	**0.27 (0.17-0.40)**	**0.34 (0.14-0.72)**	**0.24 (0.11-0.50)**
LFO	O	**0.25 (0.07-0.56)**	**0.26 (0.07-0.66)**	**0.26 (0.07-0.67)**	**0.37 (0.24-0.54)**	**0.39 (0.15-0.83)**	**0.39 (0.17-0.75)**
LFO	P	0.52 (0.18-1.19)	0.66 (0.12-2.09)	0.63 (0.12-2.01)	**0.21 (0.10-0.40)**	0.36 (0.05-1.33)	**0.24 (0.05-0.78)**
LFOB	LFOC	**1.68 (1.25-2.23)**	**1.77 (1.13-2.69)**	**1.81 (1.14-2.75)**	0.77 (0.42-1.30)	0.72 (0.27-1.55)	0.81 (0.33-1.63)
LFOB	LFOP	1.15 (0.87-1.49)	1.23 (0.77-1.89)	1.22 (0.76-1.89)	0.73 (0.43-1.14)	0.74 (0.31-1.49)	0.80 (0.37-1.49)
LFOB	LFOI	1.09 (0.74-1.54)	1.07 (0.60-1.74)	1.10 (0.62-1.81)	0.84 (0.47-1.41)	0.73 (0.27-1.59)	0.94 (0.37-1.89)
LFOB	LFOIB	**1.70 (1.30-2.18)**	**1.74 (1.16-2.54)**	**1.75 (1.16-2.55)**	1.88 (0.92-3.48)	1.82 (0.63-4.19)	2.01 (0.76-4.32)
LFOB	LFOIC	3.06 (0.95-7.65)	3.14 (0.84-8.51)	3.23 (0.86-8.77)	-	-	-
LFOB	LFOIP	**6.51 (1.95-16.6)**	**6.63 (1.52-19.5)**	**6.84 (1.58-20.1)**	**14.6 (1.47-64.8)**	13.6 (0.84-68.3)	16.5 (1.25-82.5)
LFOB	LF	**0.26 (0.18-0.35)**	**0.26 (0.16-0.41)**	**0.27 (0.16-0.42)**	**0.29 (0.17-0.47)**	**0.26 (0.10-0.54)**	**0.31 (0.13-0.59)**
LFOB	LFB	0.60 (0.31-1.05)	0.62 (0.25-1.28)	0.62 (0.25-1.27)	**0.44 (0.18-0.90)**	0.45 (0.12-1.18)	0.43 (0.13-1.05)
LFOB	LFC	**0.38 (0.18-0.71)**	**0.40 (0.13-0.93)**	**0.41 (0.14-0.96)**	-	-	-
LFOB	LFBC	**0.65 (0.44-0.92)**	0.67 (0.37-1.10)	0.67 (0.37-1.11)	0.77 (0.34-1.49)	0.85 (0.24-2.19)	0.81 (0.27-1.93)
LFOB	LFP	1.06 (0.57-1.81)	1.24 (0.51-2.60)	1.24 (0.51-2.60)	-	-	-
LFOB	CB	0.47 (0.10-1.40)	0.72 (0.09-2.78)	0.71 (0.08-2.72)	-	-	-
LFOB	CI	1.09 (0.52-2.01)	1.38 (0.43-3.42)	1.35 (0.42-3.36)	**0.30 (0.15-0.54)**	0.37 (0.08-1.08)	**0.29 (0.08-0.77)**
LFOB	CIB	1.30 (0.51-2.77)	1.91 (0.44-5.60)	1.87 (0.43-5.53)	**0.34 (0.13-0.75)**	0.48 (0.07-1.70)	0.37 (0.07-1.16)
LFOB	IO	**0.69 (0.50-0.92)**	0.74 (0.43-1.21)	0.75 (0.43-1.23)	**0.36 (0.20-0.61)**	**0.34 (0.12-0.78)**	**0.34 (0.13-0.71)**
LFOB	IOB	0.85 (0.38-1.66)	0.89 (0.31-2.03)	0.89 (0.31-2.02)	**0.25 (0.07-0.61)**	**0.27 (0.05-0.82)**	**0.26 (0.06-0.74)**
LFOB	IP	0.92 (0.43-1.72)	1.15 (0.36-2.86)	1.12 (0.34-2.79)	**0.21 (0.10-0.40)**	**0.25 (0.06-0.74)**	**0.20 (0.05-0.53)**
LFOB	FI	**0.54 (0.32-0.84)**	0.57 (0.24-1.15)	0.59 (0.25-1.23)	**0.40 (0.19-0.76)**	**0.36 (0.09-0.99)**	0.47 (0.13-1.17)
LFOB	XB	**0.47 (0.26-0.78)**	0.50 (0.21-1.03)	0.50 (0.21-1.03)	**0.40 (0.15-0.83)**	0.41 (0.10-1.13)	0.40 (0.11-1.00)
LFOB	XC	23.4 (0.17-75.3)	37.6 (0.16-94.8)	18.7 (0.15-94.9)	0.37 (0.03-1.60)	0.57 (0.02-3.05)	0.41 (0.02-2.08)
LFOB	XI	0.82 (0.16-2.49)	0.88 (0.14-2.92)	0.87 (0.13-2.91)	0.76 (0.13-2.54)	0.84 (0.09-3.36)	0.77 (0.10-2.85)
LFOB	XIB	0.86 (0.62-1.15)	0.87 (0.54-1.34)	0.86 (0.53-1.33)	0.77 (0.46-1.21)	0.76 (0.33-1.51)	0.72 (0.34-1.34)
LFOB	XIC	**3.62 (1.01-9.72)**	3.71 (0.8-11.31)	3.77 (0.80-11.6)	0.43 (0.09-1.30)	0.41 (0.04-1.65)	0.44 (0.06-1.60)
LFOB	XO	**0.72 (0.52-0.96)**	0.70 (0.42-1.09)	0.71 (0.42-1.11)	**0.55 (0.29-0.93)**	0.50 (0.17-1.15)	0.57 (0.22-1.18)

T1	T2	Overall response rate			Disease control rate		
		Fixed effects	Random effects	Meta-regression	Fixed effects	Random effects	Meta-regression
LFOB	XOB	0.78 (0.54-1.10)	0.79 (0.47-1.25)	0.79 (0.46-1.25)	**0.52 (0.27-0.92)**	0.52 (0.19-1.14)	0.50 (0.20-1.02)
LFOB	XOC	**4.32 (1.36-10.9)**	**4.37 (1.14-12.2)**	**4.44 (1.16-12.4)**	0.72 (0.19-1.98)	0.77 (0.11-2.73)	0.80 (0.14-2.60)
LFOB	XOBC	0.87 (0.54-1.34)	0.89 (0.38-1.78)	0.89 (0.38-1.78)	0.58 (0.22-1.26)	0.58 (0.12-1.74)	0.56 (0.14-1.52)
LFOB	X	**0.27 (0.12-0.50)**	**0.29 (0.10-0.65)**	**0.29 (0.10-0.65)**	**0.20 (0.07-0.43)**	**0.21 (0.04-0.61)**	**0.21 (0.05-0.55)**
LFOB	C	**0.38 (0.14-0.85)**	0.47 (0.10-1.38)	0.46 (0.10-1.35)	**0.12 (0.05-0.25)**	**0.17 (0.03-0.57)**	**0.13 (0.03-0.39)**
LFOB	I	**0.23 (0.13-0.38)**	**0.28 (0.11-0.59)**	**0.27 (0.11-0.57)**	**0.16 (0.08-0.27)**	**0.18 (0.05-0.44)**	**0.15 (0.05-0.33)**
LFOB	O	**0.21 (0.06-0.49)**	**0.23 (0.05-0.60)**	**0.23 (0.05-0.62)**	**0.22 (0.11-0.39)**	**0.21 (0.06-0.53)**	**0.24 (0.08-0.54)**
LFOB	P	0.45 (0.15-1.02)	0.57 (0.10-1.86)	0.56 (0.10-1.81)	**0.13 (0.05-0.26)**	**0.19 (0.02-0.73)**	**0.14 (0.02-0.48)**
LFOC	LFOP	**0.69 (0.50-0.93)**	0.71 (0.42-1.15)	0.70 (0.40-1.13)	0.98 (0.60-1.51)	1.15 (0.47-2.40)	1.07 (0.50-2.07)
LFOC	LFOI	**0.66 (0.44-0.95)**	0.62 (0.33-1.05)	0.63 (0.34-1.07)	1.14 (0.65-1.85)	1.13 (0.41-2.48)	1.25 (0.52-2.52)
LFOC	LFOIB	1.03 (0.71-1.43)	1.02 (0.58-1.67)	1.01 (0.57-1.66)	**2.60 (1.09-5.32)**	2.97 (0.79-8.00)	2.81 (0.86-7.03)
LFOC	LFOIC	1.84 (0.57-4.62)	1.82 (0.48-4.93)	1.83 (0.49-4.97)	-	-	-
LFOC	LFOIP	3.92 (1.16-10.1)	3.86 (0.86-11.5)	3.91 (0.87-11.6)	**19.5 (2.00-86.9)**	**20.9 (1.29-106)**	**21.9 (1.69-110)**
LFOC	LF	**0.15 (0.11-0.21)**	**0.15 (0.09-0.24)**	**0.15 (0.09-0.24)**	**0.39 (0.25-0.58)**	**0.39 (0.18-0.74)**	**0.40 (0.21-0.71)**
LFOC	LFB	**0.36 (0.17-0.66)**	**0.37 (0.13-0.81)**	**0.36 (0.13-0.79)**	0.61 (0.21-1.38)	0.74 (0.15-2.28)	0.61 (0.15-1.75)
LFOC	LFC	**0.23 (0.11-0.43)**	**0.23 (0.08-0.54)**	**0.23 (0.08-0.55)**	-	-	-
LFOC	LFBC	**0.40 (0.24-0.61)**	**0.39 (0.18-0.74)**	**0.39 (0.18-0.73)**	1.09 (0.38-2.45)	1.44 (0.27-4.61)	1.18 (0.28-3.48)
LFOC	LFP	0.64 (0.33-1.10)	0.72 (0.28-1.54)	0.71 (0.28-1.53)	-	-	-
LFOC	CB	**0.28 (0.06-0.85)**	0.42 (0.05-1.61)	0.40 (0.05-1.55)	-	-	-
LFOC	CI	0.65 (0.31-1.22)	0.80 (0.24-1.98)	0.77 (0.23-1.92)	**0.40 (0.21-0.69)**	0.56 (0.13-1.62)	0.39 (0.11-1.03)
LFOC	CIB	0.78 (0.30-1.68)	1.10 (0.25-3.25)	1.07 (0.24-3.14)	**0.46 (0.18-0.98)**	0.74 (0.12-2.57)	0.49 (0.10-1.57)
LFOC	IO	**0.41 (0.30-0.56)**	**0.43 (0.24-0.71)**	**0.43 (0.24-0.71)**	**0.49 (0.29-0.78)**	0.52 (0.20-1.13)	**0.45 (0.19-0.91)**
LFOC	IOB	0.52 (0.22-1.05)	0.53 (0.16-1.29)	0.52 (0.16-1.26)	**0.35 (0.08-0.96)**	0.46 (0.06-1.65)	0.38 (0.06-1.28)
LFOC	IP	0.55 (0.26-1.04)	0.66 (0.20-1.66)	0.64 (0.19-1.60)	**0.29 (0.14-0.52)**	0.38 (0.09-1.11)	**0.27 (0.08-0.71)**
LFOC	FI	**0.32 (0.19-0.51)**	**0.33 (0.13-0.67)**	**0.34 (0.14-0.70)**	**0.54 (0.26-0.99)**	0.55 (0.14-1.46)	0.62 (0.19-1.49)
LFOC	XB	**0.28 (0.15-0.48)**	**0.29 (0.12-0.62)**	**0.29 (0.11-0.61)**	0.54 (0.20-1.14)	0.64 (0.15-1.84)	0.54 (0.14-1.42)
LFOC	XC	13.97 (0.1-45.2)	21.7 (0.09-54.6)	10.6 (0.08-53.9)	0.50 (0.05-2.14)	0.87 (0.04-4.63)	0.55 (0.03-2.78)
LFOC	XI	0.50 (0.09-1.52)	0.51 (0.08-1.74)	0.50 (0.07-1.69)	1.04 (0.17-3.51)	1.32 (0.13-5.47)	1.05 (0.13-4.07)
LFOC	XIB	**0.52 (0.35-0.73)**	**0.51 (0.28-0.85)**	**0.50 (0.27-0.83)**	1.04 (0.58-1.74)	1.21 (0.45-2.70)	0.99 (0.41-2.08)
LFOC	XIC	2.18 (0.60-5.91)	2.16 (0.45-6.64)	2.16 (0.45-6.68)	0.58 (0.13-1.72)	0.63 (0.07-2.47)	0.59 (0.08-2.08)
LFOC	XO	**0.43 (0.31-0.58)**	**0.40 (0.23-0.64)**	**0.40 (0.23-0.64)**	0.73 (0.43-1.17)	0.76 (0.29-1.64)	0.74 (0.32-1.47)
LFOC	XOB	**0.47 (0.30-0.71)**	**0.47 (0.24-0.81)**	**0.46 (0.23-0.80)**	0.72 (0.33-1.38)	0.85 (0.24-2.18)	0.70 (0.23-1.68)
LFOC	XOC	2.60 (0.81-6.63)	2.54 (0.64-7.19)	2.54 (0.64-7.18)	0.97 (0.26-2.61)	1.16 (0.18-4.06)	1.06 (0.20-3.38)
LFOC	XOBC	**0.53 (0.31-0.85)**	0.52 (0.20-1.11)	0.51 (0.20-1.09)	0.80 (0.28-1.83)	0.94 (0.16-3.07)	0.79 (0.17-2.35)
LFOC	X	**0.16 (0.07-0.31)**	**0.17 (0.06-0.38)**	**0.17 (0.06-0.38)**	**0.27 (0.09-0.59)**	**0.32 (0.07-0.97)**	**0.28 (0.07-0.77)**
LFOC	C	**0.23 (0.08-0.52)**	**0.27 (0.06-0.80)**	**0.26 (0.06-0.77)**	**0.17 (0.07-0.32)**	**0.26 (0.05-0.86)**	**0.18 (0.04-0.53)**
LFOC	I	**0.14 (0.08-0.23)**	**0.16 (0.07-0.34)**	**0.16 (0.06-0.33)**	**0.21 (0.12-0.35)**	**0.27 (0.09-0.64)**	**0.19 (0.07-0.43)**
LFOC	O	**0.13 (0.04-0.29)**	**0.13 (0.03-0.35)**	**0.13 (0.03-0.35)**	**0.29 (0.16-0.49)**	**0.31 (0.10-0.75)**	**0.31 (0.12-0.68)**
LFOC	P	**0.27 (0.09-0.62)**	0.33 (0.06-1.07)	0.32 (0.06-1.03)	**0.17 (0.07-0.34)**	0.29 (0.04-1.11)	**0.19 (0.03-0.65)**
LFOP	LFOI	0.96 (0.63-1.40)	0.90 (0.46-1.57)	0.94 (0.48-1.65)	1.19 (0.70-1.89)	1.08 (0.38-2.39)	1.25 (0.50-2.52)

T1	T2	Overall response rate			Disease control rate		
		Fixed effects	Random effects	Meta-regression	Fixed effects	Random effects	Meta-regression
LFOP	LFOIB	**1.50 (1.05-2.09)**	1.49 (0.81-2.48)	1.50 (0.82-2.51)	**2.71 (1.20-5.38)**	2.79 (0.77-7.28)	2.77 (0.89-6.67)
LFOP	LFOIC	2.69 (0.83-6.78)	2.66 (0.68-7.35)	2.75 (0.69-7.65)	-	-	-
LFOP	LFOIP	**5.73 (1.70-14.8)**	**5.62 (1.21-16.9)**	**5.82 (1.27-17.4)**	**20.4 (2.12-91.3)**	**20.0 (1.22-100)**	**22.0 (1.69-110)**
LFOP	LF	**0.23 (0.16-0.31)**	**0.22 (0.12-0.36)**	**0.23 (0.13-0.37)**	**0.41 (0.27-0.59)**	**0.37 (0.16-0.72)**	**0.41 (0.20-0.72)**
LFOP	LFB	**0.53 (0.26-0.97)**	0.53 (0.19-1.18)	0.53 (0.19-1.18)	0.64 (0.23-1.41)	0.69 (0.14-2.09)	0.60 (0.15-1.67)
LFOP	LFC	**0.33 (0.16-0.62)**	**0.34 (0.11-0.79)**	**0.35 (0.11-0.83)**	-	-	-
LFOP	LFBC	**0.58 (0.36-0.89)**	0.57 (0.26-1.08)	0.57 (0.26-1.09)	1.13 (0.42-2.46)	1.34 (0.27-4.12)	1.16 (0.29-3.25)
LFOP	LFP	0.92 (0.53-1.48)	1.01 (0.49-1.89)	1.01 (0.49-1.90)	-	-	-
LFOP	CB	0.41 (0.08-1.24)	0.61 (0.07-2.35)	0.60 (0.07-2.31)	-	-	-
LFOP	CI	0.96 (0.45-1.78)	1.16 (0.35-2.94)	1.14 (0.34-2.89)	**0.42 (0.23-0.72)**	0.54 (0.12-1.57)	0.39 (0.11-1.02)
LFOP	CIB	1.15 (0.44-2.45)	1.60 (0.36-4.77)	1.59 (0.35-4.73)	0.48 (0.19-1.02)	0.70 (0.11-2.47)	0.49 (0.10-1.56)
LFOP	IO	**0.60 (0.43-0.82)**	0.62 (0.34-1.06)	0.63 (0.34-1.09)	**0.51 (0.31-0.80)**	0.50 (0.18-1.10)	**0.45 (0.18-0.91)**
LFOP	IOB	0.76 (0.32-1.53)	0.77 (0.23-1.87)	0.77 (0.23-1.88)	**0.37 (0.09-0.97)**	0.43 (0.06-1.48)	0.37 (0.06-1.20)
LFOP	IP	0.81 (0.37-1.52)	0.97 (0.29-2.45)	0.95 (0.28-2.40)	**0.30 (0.15-0.53)**	0.37 (0.08-1.07)	**0.27 (0.07-0.71)**
LFOP	FI	**0.47 (0.28-0.75)**	0.48 (0.19-1.00)	0.50 (0.20-1.07)	0.57 (0.28-1.02)	0.52 (0.13-1.42)	0.62 (0.18-1.51)
LFOP	XB	**0.41 (0.22-0.70)**	**0.43 (0.16-0.91)**	**0.43 (0.16-0.92)**	0.57 (0.22-1.18)	0.61 (0.14-1.73)	0.53 (0.15-1.39)
LFOP	XC	20.3 (0.15-66.3)	31.4 (0.13-78.7)	15.6 (0.12-80.9)	0.53 (0.05-2.23)	0.83 (0.03-4.40)	0.55 (0.03-2.77)
LFOP	XI	0.73 (0.14-2.22)	0.74 (0.11-2.55)	0.74 (0.11-2.54)	1.09 (0.19-3.65)	1.26 (0.12-5.21)	1.05 (0.13-3.99)
LFOP	XIB	0.76 (0.51-1.07)	0.74 (0.39-1.26)	0.74 (0.39-1.26)	1.09 (0.63-1.78)	1.15 (0.42-2.54)	0.99 (0.41-2.03)
LFOP	XIC	3.19 (0.88-8.64)	3.14 (0.64-9.78)	3.20 (0.65-9.97)	0.61 (0.14-1.79)	0.60 (0.07-2.34)	0.59 (0.08-2.09)
LFOP	XO	**0.63 (0.46-0.85)**	**0.59 (0.33-0.95)**	**0.60 (0.33-0.97)**	0.77 (0.46-1.20)	0.73 (0.27-1.59)	0.74 (0.31-1.47)
LFOP	XOB	0.69 (0.44-1.03)	0.68 (0.34-1.20)	0.68 (0.34-1.21)	0.76 (0.36-1.41)	0.80 (0.24-2.00)	0.70 (0.24-1.61)
LFOP	XOC	**3.80 (1.18-9.66)**	3.7 (0.92-10.55)	3.77 (0.93-10.7)	1.02 (0.27-2.71)	1.11 (0.17-3.85)	1.06 (0.20-3.39)
LFOP	XOBC	0.77 (0.45-1.24)	0.76 (0.29-1.63)	0.76 (0.29-1.64)	0.84 (0.30-1.88)	0.89 (0.15-2.86)	0.78 (0.17-2.27)
LFOP	X	**0.23 (0.11-0.45)**	**0.24 (0.08-0.56)**	**0.25 (0.08-0.57)**	**0.28 (0.10-0.61)**	**0.31 (0.06-0.92)**	**0.28 (0.07-0.76)**
LFOP	C	**0.34 (0.12-0.75)**	0.40 (0.08-1.18)	0.39 (0.08-1.15)	**0.17 (0.08-0.33)**	**0.25 (0.04-0.83)**	**0.18 (0.04-0.53)**
LFOP	I	**0.20 (0.11-0.33)**	**0.24 (0.09-0.51)**	**0.23 (0.09-0.49)**	**0.22 (0.13-0.36)**	**0.26 (0.08-0.63)**	**0.19 (0.07-0.43)**
LFOP	O	**0.18 (0.05-0.43)**	**0.19 (0.05-0.51)**	**0.20 (0.05-0.53)**	**0.31 (0.18-0.50)**	**0.30 (0.09-0.73)**	**0.31 (0.11-0.68)**
LFOP	P	**0.39 (0.13-0.90)**	0.48 (0.08-1.57)	0.47 (0.08-1.55)	**0.18 (0.08-0.35)**	0.28 (0.03-1.06)	**0.19 (0.03-0.64)**
LFOI	LFOIB	**1.60 (1.07-2.29)**	1.73 (0.97-2.86)	1.68 (0.94-2.77)	2.33 (1.08-4.52)	2.80 (0.91-6.80)	2.36 (0.86-5.43)
LFOI	LFOIC	2.83 (0.91-6.95)	3.02 (0.86-7.95)	2.99 (0.85-7.86)	-	-	-
LFOI	LFOIP	**5.97 (1.95-14.6)**	**6.21 (1.67-16.9)**	**6.21 (1.68-16.9)**	**17.1 (1.99-74.7)**	**18.6 (1.59-87.4)**	**17.6 (1.77-83.4)**
LFOI	LF	**0.24 (0.16-0.36)**	**0.26 (0.14-0.45)**	**0.26 (0.13-0.44)**	**0.36 (0.22-0.54)**	**0.39 (0.16-0.82)**	**0.36 (0.17-0.68)**
LFOI	LFB	0.57 (0.26-1.07)	0.62 (0.22-1.44)	0.60 (0.20-1.39)	0.55 (0.20-1.26)	0.73 (0.14-2.30)	0.53 (0.13-1.57)
LFOI	LFC	**0.35 (0.16-0.69)**	**0.39 (0.12-0.96)**	**0.39 (0.12-0.97)**	-	-	-
LFOI	LFBC	0.62 (0.36-1.00)	0.67 (0.29-1.34)	0.65 (0.28-1.30)	0.99 (0.35-2.21)	1.43 (0.27-4.57)	1.04 (0.24-3.11)
LFOI	LFP	1.00 (0.50-1.80)	1.24 (0.45-2.81)	1.19 (0.43-2.73)	-	-	-
LFOI	CB	0.44 (0.09-1.34)	0.72 (0.08-2.80)	0.68 (0.08-2.66)	-	-	-
LFOI	CI	1.02 (0.47-1.96)	1.36 (0.40-3.54)	1.29 (0.37-3.35)	**0.37 (0.20-0.62)**	0.56 (0.13-1.62)	**0.34 (0.10-0.94)**
LFOI	CIB	1.23 (0.46-2.68)	1.89 (0.41-5.75)	1.79 (0.39-5.47)	**0.42 (0.16-0.89)**	0.73 (0.12-2.57)	0.43 (0.08-1.42)

T1	T2	Overall response rate			Disease control rate		
		Fixed effects	Random effects	Meta-regression	Fixed effects	Random effects	Meta-regression
LFOI	IO	0.65 (0.43-0.94)	0.73 (0.38-1.29)	0.72 (0.37-1.27)	**0.44 (0.26-0.70)**	0.52 (0.19-1.16)	**0.39 (0.16-0.83)**
LFOI	IOB	0.81 (0.32-1.69)	0.90 (0.26-2.27)	0.87 (0.25-2.20)	**0.32 (0.07-0.86)**	0.46 (0.06-1.65)	0.33 (0.05-1.14)
LFOI	IP	0.86 (0.39-1.67)	1.13 (0.33-2.95)	1.07 (0.31-2.79)	**0.26 (0.13-0.46)**	0.38 (0.09-1.11)	**0.24 (0.06-0.65)**
LFOI	FI	**0.51 (0.29-0.83)**	0.56 (0.22-1.19)	0.57 (0.22-1.22)	**0.49 (0.24-0.89)**	0.54 (0.14-1.48)	0.54 (0.17-1.31)
LFOI	XB	**0.44 (0.22-0.78)**	0.50 (0.19-1.11)	0.48 (0.18-1.07)	0.49 (0.19-1.05)	0.64 (0.14-1.92)	0.47 (0.12-1.29)
LFOI	XC	22.7 (0.16-70.9)	37.3 (0.15-93.1)	17.7 (0.14-90.1)	0.46 (0.04-1.94)	0.86 (0.03-4.61)	0.48 (0.03-2.49)
LFOI	XI	0.78 (0.14-2.40)	0.87 (0.13-3.03)	0.84 (0.12-2.89)	0.95 (0.16-3.21)	1.32 (0.13-5.56)	0.92 (0.11-3.61)
LFOI	XIB	0.81 (0.52-1.21)	0.87 (0.44-1.55)	0.83 (0.42-1.48)	0.95 (0.53-1.58)	1.21 (0.42-2.81)	0.87 (0.34-1.92)
LFOI	XIC	3.42 (0.91-9.38)	3.69 (0.73-11.8)	3.63 (0.71-11.5)	0.53 (0.11-1.59)	0.63 (0.07-2.59)	0.52 (0.07-1.91)
LFOI	XO	**0.67 (0.44-0.99)**	0.69 (0.36-1.21)	0.68 (0.35-1.19)	0.67 (0.38-1.10)	0.77 (0.26-1.81)	0.66 (0.26-1.42)
LFOI	XOB	0.74 (0.44-1.16)	0.79 (0.38-1.48)	0.76 (0.36-1.43)	0.66 (0.30-1.25)	0.84 (0.23-2.21)	0.62 (0.20-1.53)
LFOI	XOC	4.07 (1.22-10.6)	4.35 (1.04-12.8)	4.27 (1.01-12.4)	0.89 (0.23-2.41)	1.18 (0.17-4.28)	0.94 (0.17-3.12)
LFOI	XOBC	0.83 (0.45-1.39)	0.89 (0.33-1.98)	0.86 (0.31-1.91)	0.73 (0.25-1.66)	0.94 (0.16-3.12)	0.69 (0.15-2.12)
LFOI	X	**0.25 (0.11-0.50)**	**0.29 (0.09-0.68)**	**0.28 (0.09-0.66)**	**0.24 (0.09-0.54)**	0.32 (0.06-1.02)	**0.24 (0.06-0.70)**
LFOI	C	**0.36 (0.12-0.82)**	0.47 (0.10-1.42)	0.44 (0.09-1.33)	**0.15 (0.07-0.29)**	**0.26 (0.05-0.86)**	**0.15 (0.03-0.49)**
LFOI	I	**0.22 (0.11-0.37)**	**0.28 (0.10-0.62)**	**0.26 (0.10-0.58)**	**0.19 (0.11-0.32)**	**0.27 (0.09-0.65)**	**0.17 (0.06-0.40)**
LFOI	O	**0.20 (0.05-0.47)**	**0.23 (0.05-0.62)**	**0.22 (0.05-0.62)**	**0.27 (0.15-0.46)**	**0.32 (0.09-0.82)**	**0.27 (0.09-0.64)**
LFOI	P	**0.42 (0.14-0.99)**	0.56 (0.09-1.90)	0.53 (0.09-1.77)	**0.15 (0.07-0.30)**	0.29 (0.04-1.10)	**0.17 (0.03-0.59)**
LFOIB	LFOIC	1.82 (0.56-4.60)	1.85 (0.48-5.08)	1.89 (0.49-5.21)	-	-	-
LFOIB	LFOIP	**3.88 (1.16-9.94)**	3.88 (0.88-11.4)	3.99 (0.92-11.7)	8.41 (0.78-38.3)	8.61 (0.49-43.4)	9.27 (0.66-46.6)
LFOIB	LF	**0.15 (0.10-0.22)**	**0.15 (0.08-0.26)**	**0.16 (0.09-0.27)**	**0.17 (0.07-0.33)**	**0.17 (0.05-0.44)**	**0.18 (0.06-0.42)**
LFOIB	LFB	**0.36 (0.18-0.65)**	**0.37 (0.14-0.81)**	**0.37 (0.13-0.81)**	**0.26 (0.08-0.64)**	0.31 (0.05-1.00)	**0.26 (0.05-0.78)**
LFOIB	LFC	**0.23 (0.10-0.43)**	**0.24 (0.07-0.57)**	**0.24 (0.08-0.58)**	-	-	-
LFOIB	LFBC	**0.39 (0.24-0.60)**	**0.40 (0.19-0.73)**	**0.40 (0.19-0.73)**	0.46 (0.15-1.09)	0.59 (0.10-1.93)	0.49 (0.11-1.48)
LFOIB	LFP	0.63 (0.32-1.12)	0.74 (0.28-1.61)	0.73 (0.28-1.62)	-	-	-
LFOIB	CB	**0.28 (0.06-0.84)**	0.43 (0.05-1.67)	0.42 (0.05-1.62)	-	-	-
LFOIB	CI	0.65 (0.30-1.22)	0.82 (0.24-2.09)	0.80 (0.23-2.03)	**0.18 (0.07-0.37)**	**0.24 (0.04-0.81)**	**0.17 (0.04-0.52)**
LFOIB	CIB	0.78 (0.29-1.68)	1.13 (0.25-3.39)	1.10 (0.24-3.32)	**0.20 (0.06-0.49)**	0.32 (0.04-1.23)	**0.22 (0.03-0.76)**
LFOIB	IO	**0.41 (0.28-0.58)**	**0.44 (0.23-0.76)**	**0.44 (0.23-0.77)**	**0.21 (0.09-0.43)**	**0.23 (0.06-0.62)**	**0.20 (0.06-0.50)**
LFOIB	IOB	0.51 (0.22-1.03)	0.53 (0.17-1.29)	0.53 (0.17-1.28)	**0.15 (0.03-0.42)**	**0.19 (0.02-0.69)**	**0.16 (0.02-0.54)**
LFOIB	IP	0.55 (0.25-1.05)	0.68 (0.20-1.74)	0.66 (0.19-1.69)	**0.12 (0.05-0.27)**	**0.17 (0.03-0.55)**	**0.12 (0.02-0.36)**
LFOIB	FI	**0.32 (0.19-0.52)**	**0.33 (0.13-0.70)**	**0.35 (0.14-0.75)**	**0.24 (0.09-0.52)**	**0.24 (0.04-0.75)**	**0.27 (0.06-0.77)**
LFOIB	XB	**0.28 (0.15-0.48)**	**0.30 (0.12-0.63)**	**0.30 (0.12-0.63)**	**0.23 (0.07-0.57)**	**0.28 (0.05-0.89)**	**0.23 (0.05-0.70)**
LFOIB	XC	14.3 (0.10-44.9)	21.9 (0.09-55.9)	11.0 (0.09-55.3)	**0.22 (0.02-0.97)**	0.38 (0.01-2.10)	0.24 (0.01-1.29)
LFOIB	XI	0.49 (0.09-1.50)	0.52 (0.08-1.76)	0.51 (0.08-1.74)	0.45 (0.07-1.60)	0.57 (0.05-2.49)	0.46 (0.05-1.85)
LFOIB	XIB	**0.51 (0.35-0.72)**	**0.52 (0.29-0.86)**	**0.51 (0.28-0.85)**	**0.45 (0.19-0.90)**	0.52 (0.14-1.36)	0.43 (0.14-1.05)
LFOIB	XIC	2.17 (0.59-5.89)	2.20 (0.45-6.89)	2.23 (0.45-7.00)	**0.26 (0.05-0.83)**	0.28 (0.02-1.20)	0.26 (0.03-1.02)
LFOIB	XO	**0.43 (0.29-0.61)**	**0.41 (0.22-0.70)**	**0.42 (0.22-0.71)**	**0.32 (0.13-0.66)**	**0.34 (0.08-0.93)**	**0.33 (0.09-0.83)**
LFOIB	XOB	**0.47 (0.30-0.69)**	**0.47 (0.25-0.82)**	**0.47 (0.24-0.81)**	**0.31 (0.12-0.67)**	0.36 (0.08-1.00)	**0.30 (0.08-0.79)**
LFOIB	XOC	2.58 (0.79-6.62)	2.59 (0.64-7.43)	2.62 (0.64-7.53)	0.43 (0.09-1.27)	0.51 (0.06-2.01)	0.47 (0.07-1.69)

T1	T2	Overall response rate			Disease control rate		
		**Fixed effects**	**Random effects**	**Meta-regression**	**Fixed effects**	**Random effects**	**Meta-regression**
LFOIB	XOBC	**0.52 (0.31-0.84)**	0.53 (0.21-1.12)	0.53 (0.20-1.12)	**0.35 (0.10-0.86)**	0.40 (0.06-1.39)	0.34 (0.06-1.07)
LFOIB	X	**0.16 (0.07-0.31)**	**0.17 (0.06-0.40)**	**0.17 (0.06-0.40)**	**0.12 (0.03-0.29)**	**0.14 (0.02-0.48)**	**0.12 (0.02-0.38)**
LFOIB	C	**0.23 (0.08-0.52)**	**0.28 (0.06-0.84)**	**0.27 (0.06-0.81)**	**0.07 (0.03-0.17)**	**0.11 (0.02-0.42)**	**0.08 (0.01-0.26)**
LFOIB	I	**0.14 (0.07-0.23)**	**0.17 (0.06-0.36)**	**0.16 (0.06-0.35)**	**0.09 (0.04-0.19)**	**0.12 (0.03-0.34)**	**0.09 (0.02-0.23)**
LFOIB	O	**0.13 (0.03-0.30)**	**0.14 (0.03-0.37)**	**0.14 (0.03-0.38)**	**0.13 (0.05-0.27)**	**0.14 (0.03-0.41)**	**0.14 (0.03-0.37)**
LFOIB	P	**0.27 (0.09-0.62)**	0.34 (0.06-1.11)	0.33 (0.06-1.08)	**0.07 (0.02-0.17)**	**0.13 (0.01-0.52)**	**0.08 (0.01-0.31)**
LFOIC	LFOIP	2.76 (0.51-8.88)	2.84 (0.41-10.1)	2.86 (0.42-10.2)	-	-	-
LFOIC	LF	**0.11 (0.03-0.27)**	**0.11 (0.03-0.31)**	**0.12 (0.03-0.31)**	-	-	-
LFOIC	LFB	**0.26 (0.06-0.71)**	**0.28 (0.05-0.87)**	**0.27 (0.05-0.85)**	-	-	-
LFOIC	LFC	**0.16 (0.04-0.45)**	**0.18 (0.03-0.56)**	**0.18 (0.03-0.57)**	-	-	-
LFOIC	LFBC	**0.28 (0.08-0.72)**	**0.30 (0.07-0.86)**	**0.29 (0.06-0.84)**	-	-	-
LFOIC	LFP	0.46 (0.12-1.21)	0.55 (0.11-1.70)	0.54 (0.11-1.65)	-	-	-
LFOIC	CB	**0.20 (0.03-0.74)**	0.32 (0.02-1.41)	0.31 (0.02-1.37)	-	-	-
LFOIC	CI	0.47 (0.11-1.28)	0.61 (0.10-2.01)	0.58 (0.10-1.95)	-	-	-
LFOIC	CIB	0.56 (0.12-1.64)	0.84 (0.11-3.09)	0.81 (0.11-3.00)	-	-	-
LFOIC	IO	**0.30 (0.09-0.72)**	**0.33 (0.08-0.88)**	**0.32 (0.08-0.87)**	-	-	-
LFOIC	IOB	0.37 (0.08-1.06)	0.40 (0.07-1.31)	0.39 (0.07-1.29)	-	-	-
LFOIC	IP	0.39 (0.09-1.09)	0.50 (0.08-1.66)	0.48 (0.08-1.62)	-	-	-
LFOIC	FI	**0.23 (0.06-0.59)**	**0.25 (0.05-0.74)**	**0.25 (0.05-0.76)**	-	-	-
LFOIC	XB	**0.20 (0.05-0.53)**	**0.22 (0.05-0.68)**	**0.22 (0.04-0.66)**	-	-	-
LFOIC	XC	10.9 (0.05-32.1)	19.8 (0.05-40.8)	8.11 (0.04-40.8)	-	-	-
LFOIC	XI	0.36 (0.04-1.33)	0.39 (0.04-1.58)	0.38 (0.03-1.55)	-	-	-
LFOIC	XIB	**0.37 (0.11-0.91)**	0.39 (0.10-1.05)	0.37 (0.09-1.02)	-	-	-
LFOIC	XIC	1.56 (0.25-5.28)	1.65 (0.20-6.30)	1.63 (0.20-6.29)	-	-	-
LFOIC	XO	**0.31 (0.09-0.76)**	**0.31 (0.08-0.83)**	**0.30 (0.08-0.83)**	-	-	-
LFOIC	XOB	**0.34 (0.10-0.85)**	**0.35 (0.08-0.98)**	**0.34 (0.08-0.96)**	-	-	-
LFOIC	XOC	1.86 (0.33-6.08)	1.94 (0.27-7.02)	1.92 (0.27-6.95)	-	-	-
LFOIC	XOBC	**0.38 (0.10-0.97)**	0.40 (0.08-1.20)	0.39 (0.08-1.18)	-	-	-
LFOIC	X	**0.11 (0.03-0.32)**	**0.13 (0.02-0.41)**	**0.13 (0.02-0.40)**	-	-	-
LFOIC	C	**0.16 (0.03-0.50)**	**0.21 (0.03-0.77)**	**0.20 (0.03-0.74)**	-	-	-
LFOIC	I	**0.10 (0.03-0.26)**	**0.12 (0.03-0.37)**	**0.12 (0.02-0.36)**	-	-	-
LFOIC	O	**0.09 (0.02-0.28)**	**0.10 (0.01-0.35)**	**0.10 (0.01-0.35)**	-	-	-
LFOIC	P	**0.19 (0.04-0.59)**	**0.25 (0.03-1.00)**	**0.24 (0.03-0.96)**	-	-	-
LFOIP	LF	**0.05 (0.01-0.13)**	**0.06 (0.01-0.17)**	**0.06 (0.01-0.17)**	**0.05 (0.00-0.19)**	**0.06 (0.00-0.27)**	**0.05 (0.00-0.22)**
LFOIP	LFB	**0.12 (0.03-0.34)**	**0.14 (0.02-0.47)**	**0.14 (0.02-0.45)**	**0.08 (0.01-0.32)**	**0.12 (0.00-0.58)**	**0.08 (0.00-0.38)**
LFOIP	LFC	**0.08 (0.02-0.22)**	**0.09 (0.01-0.31)**	**0.09 (0.01-0.31)**	-	-	-
LFOIP	LFBC	**0.14 (0.04-0.35)**	**0.15 (0.03-0.47)**	**0.15 (0.03-0.46)**	**0.14 (0.01-0.58)**	0.23 (0.01-1.15)	**0.16 (0.01-0.74)**
LFOIP	LFP	**0.22 (0.05-0.59)**	**0.28 (0.05-0.94)**	**0.27 (0.05-0.90)**	-	-	-
LFOIP	CB	**0.10 (0.01-0.36)**	**0.16 (0.01-0.77)**	**0.15 (0.01-0.72)**	-	-	-
LFOIP	CI	**0.22 (0.05-0.62)**	0.31 (0.05-1.11)	0.29 (0.04-1.04)	**0.05 (0.00-0.20)**	**0.09 (0.00-0.43)**	**0.05 (0.00-0.23)**

T1	T2	Overall response rate			Disease control rate		
		Fixed effects	Random effects	Meta-regression	Fixed effects	Random effects	Meta-regression
LFOIP	CIB	**0.27 (0.05-0.80)**	0.43 (0.05-1.69)	0.41 (0.05-1.61)	**0.06 (0.00-0.24)**	**0.12 (0.00-0.60)**	**0.07 (0.00-0.32)**
LFOIP	IO	**0.14 (0.04-0.35)**	**0.17 (0.04-0.49)**	**0.16 (0.03-0.48)**	**0.06 (0.01-0.23)**	**0.08 (0.00-0.37)**	**0.06 (0.00-0.25)**
LFOIP	IOB	**0.18 (0.04-0.51)**	**0.21 (0.03-0.72)**	**0.20 (0.03-0.69)**	**0.05 (0.00-0.20)**	**0.07 (0.00-0.39)**	**0.05 (0.00-0.25)**
LFOIP	IP	**0.19 (0.04-0.52)**	**0.26 (0.04-0.92)**	**0.24 (0.04-0.86)**	**0.04 (0.00-0.14)**	**0.06 (0.00-0.29)**	**0.04 (0.00-0.16)**
LFOIP	FI	**0.11 (0.03-0.29)**	**0.13 (0.02-0.40)**	**0.13 (0.02-0.41)**	**0.07 (0.01-0.27)**	**0.09 (0.00-0.41)**	**0.08 (0.00-0.35)**
LFOIP	XB	**0.10 (0.02-0.26)**	**0.11 (0.02-0.37)**	**0.11 (0.02-0.36)**	**0.07 (0.01-0.28)**	**0.10 (0.00-0.50)**	**0.07 (0.00-0.32)**
LFOIP	XC	4.62 (0.02-15.3)	8.00 (0.02-20.3)	4.13 (0.02-20.5)	**0.07 (0.00-0.36)**	**0.14 (0.00-0.87)**	**0.07 (0.00-0.45)**
LFOIP	XI	**0.17 (0.02-0.63)**	**0.20 (0.02-0.85)**	**0.19 (0.02-0.81)**	**0.14 (0.01-0.66)**	0.21 (0.00-1.19)	**0.14 (0.00-0.73)**
LFOIP	XIB	**0.18 (0.05-0.44)**	**0.20 (0.04-0.58)**	**0.19 (0.04-0.55)**	**0.14 (0.01-0.50)**	**0.19 (0.01-0.86)**	**0.13 (0.01-0.56)**
LFOIP	XIC	0.74 (0.12-2.55)	0.84 (0.09-3.38)	0.82 (0.09-3.27)	**0.08 (0.00-0.35)**	**0.10 (0.00-0.56)**	**0.08 (0.00-0.41)**
LFOIP	XO	**0.15 (0.04-0.37)**	**0.16 (0.03-0.46)**	**0.15 (0.03-0.45)**	**0.10 (0.01-0.36)**	**0.12 (0.01-0.55)**	**0.10 (0.01-0.42)**
LFOIP	XOB	**0.16 (0.04-0.41)**	**0.18 (0.04-0.54)**	**0.17 (0.04-0.52)**	**0.09 (0.01-0.36)**	**0.13 (0.01-0.63)**	**0.09 (0.01-0.41)**
LFOIP	XOC	0.89 (0.15-2.94)	0.99 (0.12-3.77)	0.97 (0.12-3.64)	**0.13 (0.01-0.56)**	0.19 (0.01-1.00)	**0.14 (0.01-0.71)**
LFOIP	XOBC	**0.18 (0.05-0.47)**	**0.20 (0.04-0.66)**	**0.20 (0.03-0.64)**	**0.11 (0.01-0.43)**	**0.15 (0.01-0.77)**	**0.10 (0.00-0.49)**
LFOIP	X	**0.05 (0.01-0.15)**	**0.07 (0.01-0.22)**	**0.06 (0.01-0.21)**	**0.04 (0.00-0.14)**	**0.05 (0.00-0.26)**	**0.04 (0.00-0.17)**
LFOIP	C	**0.08 (0.02-0.24)**	**0.11 (0.01-0.42)**	**0.10 (0.01-0.39)**	**0.02 (0.00-0.08)**	**0.04 (0.00-0.21)**	**0.02 (0.00-0.11)**
LFOIP	I	**0.05 (0.01-0.12)**	**0.06 (0.01-0.21)**	**0.06 (0.01-0.19)**	**0.03 (0.00-0.10)**	**0.04 (0.00-0.19)**	**0.03 (0.00-0.11)**
LFOIP	O	**0.04 (0.01-0.14)**	**0.05 (0.01-0.19)**	**0.05 (0.01-0.19)**	**0.04 (0.00-0.14)**	**0.05 (0.00-0.23)**	**0.04 (0.00-0.18)**
LFOIP	P	**0.09 (0.02-0.29)**	**0.13 (0.01-0.54)**	**0.12 (0.01-0.50)**	**0.02 (0.00-0.09)**	**0.05 (0.00-0.25)**	**0.03 (0.00-0.13)**
LF	LFB	**2.39 (1.13-4.45)**	2.52 (0.90-5.67)	2.45 (0.86-5.53)	1.59 (0.58-3.52)	1.98 (0.42-5.98)	1.57 (0.40-4.43)
LF	LFC	1.47 (0.76-2.58)	1.53 (0.61-3.25)	1.54 (0.60-3.26)	-	-	-
LF	LFBC	**2.61 (1.55-4.12)**	**2.72 (1.23-5.25)**	**2.65 (1.19-5.14)**	**2.84 (1.04-6.21)**	3.87 (0.77-12.1)	3.04 (0.74-8.79)
LF	LFP	**4.20 (2.17-7.35)**	**4.97 (1.93-10.8)**	**4.83 (1.85-10.5)**	-	-	-
LF	CB	1.86 (0.38-5.57)	2.88 (0.34-11.1)	2.74 (0.32-10.6)	-	-	-
LF	CI	**4.30 (2.03-8.06)**	**5.49 (1.67-13.8)**	**5.24 (1.58-13.2)**	1.05 (0.60-1.71)	1.50 (0.39-4.14)	1.00 (0.31-2.57)
LF	CIB	**5.16 (1.98-11.1)**	**7.58 (1.74-22.5)**	**7.27 (1.63-21.6)**	1.19 (0.48-2.48)	1.96 (0.34-6.65)	1.26 (0.27-3.96)
LF	IO	**2.72 (1.90-3.79)**	**2.95 (1.64-4.94)**	**2.90 (1.61-4.88)**	1.27 (0.81-1.89)	1.39 (0.59-2.83)	1.15 (0.54-2.20)
LF	IOB	**3.42 (1.40-7.02)**	**3.64 (1.10-8.99)**	**3.54 (1.07-8.78)**	0.91 (0.21-2.46)	1.24 (0.17-4.36)	0.98 (0.16-3.23)
LF	IP	**3.63 (1.67-6.92)**	**4.57 (1.39-11.5)**	**4.35 (1.30-11.0)**	0.74 (0.39-1.28)	1.02 (0.26-2.83)	0.69 (0.21-1.77)
LF	FI	**2.13 (1.25-3.41)**	2.25 (0.91-4.66)	2.30 (0.93-4.82)	1.41 (0.73-2.46)	1.46 (0.41-3.74)	1.58 (0.53-3.64)
LF	XB	1.86 (0.97-3.23)	2.03 (0.79-4.34)	1.97 (0.76-4.22)	1.41 (0.56-2.89)	1.71 (0.42-4.73)	1.38 (0.39-3.52)
LF	XC	90.4 (0.67-297)	147 (0.60-369)	74.0 (0.56-370)	1.30 (0.12-5.51)	2.32 (0.1-12.23)	1.41 (0.08-7.09)
LF	XI	3.28 (0.61-10.1)	3.53 (0.52-12.2)	3.40 (0.5-11.69)	2.70 (0.46-9.05)	3.56 (0.37-14.5)	2.70 (0.33-10.4)
LF	XIB	**3.41 (2.25-4.96)**	**3.52 (1.88-6.04)**	**3.39 (1.79-5.83)**	2.72 (1.60-4.33)	3.25 (1.29-6.94)	2.55 (1.13-5.19)
LF	XIC	**14.4 (3.91-39.0)**	**14.8 (3.08-45.8)**	**14.7 (3.03-45.6)**	1.51 (0.34-4.38)	1.66 (0.20-6.35)	1.49 (0.23-5.21)
LF	XO	**2.83 (2.01-3.90)**	**2.76 (1.62-4.39)**	**2.73 (1.60-4.34)**	**1.90 (1.23-2.81)**	2.01 (0.90-3.95)	1.88 (0.94-3.44)
LF	XOB	**3.11 (1.92-4.78)**	**3.21 (1.60-5.79)**	**3.11 (1.54-5.64)**	1.88 (0.90-3.48)	2.27 (0.70-5.65)	1.80 (0.64-4.20)
LF	XOC	**17.1 (5.29-43.8)**	**17.5 (4.40-49.3)**	**17.3 (4.34-48.8)**	2.52 (0.70-6.59)	3.08 (0.53-10.4)	2.69 (0.55-8.36)
LF	XOBC	**3.48 (1.96-5.73)**	**3.61 (1.37-7.79)**	**3.51 (1.32-7.59)**	2.09 (0.75-4.66)	2.53 (0.46-8.10)	2.02 (0.46-5.95)
LF	X	1.06 (0.47-2.04)	1.15 (0.40-2.65)	1.13 (0.39-2.59)	0.69 (0.26-1.49)	0.86 (0.20-2.47)	0.71 (0.19-1.90)

T1	T2	Overall response rate			Disease control rate		
		Fixed effects	Random effects	Meta-regression	Fixed effects	Random effects	Meta-regression
LF	C	1.52 (0.53-3.40)	1.87 (0.40-5.53)	1.79 (0.38-5.25)	**0.43 (0.21-0.80)**	0.70 (0.14-2.22)	0.45 (0.11-1.34)
LF	I	0.91 (0.49-1.52)	1.12 (0.44-2.38)	1.06 (0.41-2.26)	**0.55 (0.33-0.86)**	0.72 (0.27-1.62)	0.49 (0.20-1.07)
LF	O	0.83 (0.24-1.91)	0.90 (0.22-2.31)	0.90 (0.22-2.33)	0.76 (0.49-1.12)	0.81 (0.32-1.74)	0.78 (0.35-1.51)
LF	P	1.76 (0.58-4.10)	2.27 (0.39-7.41)	2.17 (0.38-7.04)	**0.44 (0.20-0.85)**	0.77 (0.11-2.89)	0.48 (0.09-1.63)
LFB	LFC	0.69 (0.25-1.57)	0.76 (0.18-2.17)	0.79 (0.18-2.27)	-	-	-
LFB	LFBC	1.20 (0.55-2.30)	1.27 (0.43-2.97)	1.28 (0.42-3.03)	2.09 (0.60-5.34)	2.69 (0.39-9.53)	2.50 (0.46-8.07)
LFB	LFP	1.95 (0.77-4.12)	2.36 (0.65-6.28)	2.37 (0.64-6.38)	-	-	-
LFB	CB	0.87 (0.15-2.81)	1.38 (0.13-5.80)	1.35 (0.13-5.70)	-	-	-
LFB	CI	2.00 (0.73-4.43)	2.63 (0.58-7.82)	2.59 (0.56-7.80)	0.80 (0.27-1.88)	1.14 (0.15-4.21)	0.88 (0.15-2.92)
LFB	CIB	2.40 (0.73-5.91)	3.63 (0.62-12.3)	3.59 (0.6-12.25)	0.91 (0.24-2.45)	1.50 (0.14-6.26)	1.11 (0.14-4.17)
LFB	IO	1.26 (0.61-2.34)	1.41 (0.49-3.23)	1.43 (0.50-3.32)	0.97 (0.34-2.19)	1.06 (0.20-3.34)	1.02 (0.23-2.91)
LFB	IOB	1.58 (0.54-3.66)	1.70 (0.41-4.77)	1.71 (0.40-4.87)	0.68 (0.13-2.02)	0.86 (0.09-3.37)	0.81 (0.10-2.95)
LFB	IP	1.69 (0.60-3.79)	2.18 (0.48-6.52)	2.14 (0.46-6.45)	0.57 (0.18-1.37)	0.78 (0.10-2.89)	0.61 (0.10-2.02)
LFB	FI	0.99 (0.43-1.98)	1.07 (0.30-2.78)	1.14 (0.31-2.99)	1.08 (0.34-2.61)	1.11 (0.16-3.94)	1.41 (0.25-4.48)
LFB	XB	0.86 (0.36-1.75)	0.95 (0.28-2.42)	0.95 (0.28-2.45)	1.05 (0.30-2.66)	1.23 (0.19-4.23)	1.17 (0.22-3.64)
LFB	XC	42.0 (0.27-138)	73.2 (0.24-174)	35.4 (0.23-181)	1.00 (0.07-4.56)	1.76 (0.05-10.1)	1.24 (0.05-6.70)
LFB	XI	1.51 (0.25-4.93)	1.64 (0.21-6.09)	1.64 (0.20-6.10)	2.01 (0.28-7.34)	2.50 (0.19-11.2)	2.24 (0.22-9.20)
LFB	XIB	1.57 (0.78-2.85)	1.64 (0.63-3.54)	1.63 (0.62-3.57)	2.02 (0.80-4.23)	2.28 (0.56-6.31)	2.12 (0.61-5.40)
LFB	XIC	**6.63 (1.54-19.4)**	**6.99 (1.15-24.0)**	**7.15 (1.16-24.9)**	1.15 (0.19-3.87)	1.25 (0.09-5.67)	1.32 (0.12-5.42)
LFB	XO	1.32 (0.64-2.44)	1.32 (0.47-2.96)	1.35 (0.48-3.06)	1.46 (0.51-3.33)	1.55 (0.29-4.87)	1.68 (0.39-4.79)
LFB	XOB	1.41 (0.73-2.50)	1.46 (0.60-3.01)	1.46 (0.60-3.04)	1.34 (0.58-2.64)	1.46 (0.42-3.72)	1.40 (0.47-3.27)
LFB	XOC	**7.90 (2.05-22.0)**	**8.24 (1.60-26.4)**	**8.40 (1.64-27.1)**	1.92 (0.37-6.01)	2.32 (0.23-9.69)	2.37 (0.29-9.01)
LFB	XOBC	1.58 (0.76-2.94)	1.65 (0.53-3.99)	1.66 (0.52-4.02)	1.49 (0.49-3.49)	1.64 (0.28-5.40)	1.57 (0.33-4.64)
LFB	X	0.49 (0.18-1.09)	0.54 (0.14-1.49)	0.55 (0.14-1.50)	0.52 (0.14-1.37)	0.63 (0.09-2.28)	0.61 (0.10-2.01)
LFB	C	0.71 (0.20-1.79)	0.89 (0.15-3.02)	0.88 (0.14-2.99)	**0.33 (0.10-0.83)**	0.53 (0.06-2.14)	0.40 (0.06-1.43)
LFB	I	0.42 (0.17-0.87)	0.53 (0.15-1.42)	0.52 (0.14-1.39)	**0.42 (0.14-0.97)**	0.55 (0.09-1.81)	0.44 (0.09-1.31)
LFB	O	0.39 (0.09-1.02)	0.44 (0.08-1.36)	0.45 (0.08-1.40)	0.59 (0.20-1.37)	0.64 (0.10-2.18)	0.71 (0.14-2.15)
LFB	P	0.82 (0.22-2.14)	1.09 (0.15-3.98)	1.07 (0.14-3.92)	**0.33 (0.10-0.86)**	0.59 (0.05-2.65)	0.43 (0.05-1.68)
LFC	LFBC	1.96 (0.83-3.97)	2.13 (0.60-5.49)	2.07 (0.58-5.39)	-	-	-
LFC	LFP	**3.16 (1.21-6.81)**	3.89 (0.99-10.8)	3.78 (0.94-10.6)	-	-	-
LFC	CB	1.40 (0.24-4.56)	2.27 (0.20-9.73)	2.15 (0.19-9.26)	-	-	-
LFC	CI	**3.24 (1.15-7.27)**	4.32 (0.89-13.3)	4.11 (0.84-12.7)	-	-	-
LFC	CIB	**3.88 (1.17-9.71)**	5.96 (0.96-20.9)	5.71 (0.91-19.9)	-	-	-
LFC	IO	2.05 (0.95-3.86)	2.31 (0.74-5.55)	2.27 (0.73-5.50)	-	-	-
LFC	IOB	2.57 (0.82-6.19)	2.85 (0.59-8.64)	2.77 (0.57-8.41)	-	-	-
LFC	IP	2.73 (0.95-6.19)	3.58 (0.74-11.0)	3.41 (0.69-10.5)	-	-	-
LFC	FI	1.60 (0.67-3.25)	1.77 (0.46-4.76)	1.81 (0.46-4.92)	-	-	-
LFC	XB	1.40 (0.54-3.00)	1.59 (0.40-4.36)	1.54 (0.38-4.26)	-	-	-
LFC	XC	66.7 (0.44-224)	123 (0.37-291)	58.4 (0.35-289)	-	-	-
LFC	XI	2.47 (0.39-8.26)	2.77 (0.30-10.8)	2.67 (0.29-10.4)	-	-	-

T1	T2	Overall response rate			Disease control rate		
		**Fixed effects**	**Random effects**	**Meta-regression**	**Fixed effects**	**Random effects**	**Meta-regression**
LFC	XIB	**2.57 (1.15-4.97)**	2.76 (0.86-6.69)	2.65 (0.82-6.44)	-	-	-
LFC	XIC	**10.8 (2.39-32.0)**	**11.6 (1.73-41.5)**	**11.5 (1.70-41.1)**	-	-	-
LFC	XO	2.13 (0.99-4.00)	2.16 (0.72-5.04)	2.14 (0.70-5.01)	-	-	-
LFC	XOB	**2.34 (1.01-4.66)**	2.51 (0.75-6.26)	2.43 (0.72-6.08)	-	-	-
LFC	XOC	**12.9 (3.19-36.6)**	**13.7 (2.41-45.8)**	**13.5 (2.37-44.8)**	-	-	-
LFC	XOBC	**2.62 (1.06-5.42)**	2.83 (0.70-7.80)	2.75 (0.67-7.59)	-	-	-
LFC	X	0.79 (0.27-1.84)	0.90 (0.21-2.60)	0.88 (0.20-2.55)	-	-	-
LFC	C	1.14 (0.32-2.94)	1.47 (0.23-5.08)	1.40 (0.22-4.86)	-	-	-
LFC	I	0.68 (0.27-1.43)	0.88 (0.22-2.41)	0.83 (0.21-2.29)	-	-	-
LFC	O	0.62 (0.15-1.65)	0.71 (0.12-2.21)	0.70 (0.12-2.21)	-	-	-
LFC	P	1.33 (0.35-3.50)	1.78 (0.23-6.64)	1.70 (0.22-6.35)	-	-	-
LFBC	LFP	1.68 (0.80-3.14)	2.02 (0.67-4.79)	2.01 (0.67-4.79)	-	-	-
LFBC	CB	0.75 (0.14-2.30)	1.18 (0.12-4.73)	1.15 (0.12-4.65)	-	-	-
LFBC	CI	1.73 (0.74-3.44)	2.24 (0.58-6.14)	2.20 (0.57-6.05)	0.45 (0.15-1.05)	0.60 (0.08-2.27)	0.47 (0.08-1.56)
LFBC	CIB	2.07 (0.73-4.67)	3.09 (0.62-9.83)	3.05 (0.60-9.76)	0.51 (0.14-1.36)	0.79 (0.07-3.35)	0.59 (0.07-2.22)
LFBC	IO	1.09 (0.66-1.71)	1.20 (0.53-2.39)	1.22 (0.53-2.43)	0.54 (0.19-1.23)	0.56 (0.10-1.80)	0.54 (0.12-1.57)
LFBC	IOB	1.36 (0.54-2.82)	1.45 (0.42-3.66)	1.44 (0.42-3.65)	0.37 (0.07-1.08)	0.44 (0.05-1.71)	0.42 (0.06-1.47)
LFBC	IP	1.46 (0.61-2.94)	1.86 (0.49-5.11)	1.82 (0.47-5.03)	**0.32 (0.10-0.76)**	0.41 (0.05-1.54)	0.32 (0.05-1.08)
LFBC	FI	0.85 (0.45-1.48)	0.92 (0.31-2.12)	0.97 (0.32-2.26)	0.60 (0.19-1.45)	0.59 (0.08-2.11)	0.74 (0.13-2.38)
LFBC	XB	0.74 (0.36-1.36)	0.82 (0.28-1.90)	0.82 (0.28-1.90)	0.59 (0.16-1.54)	0.67 (0.09-2.42)	0.63 (0.11-2.06)
LFBC	XC	37.7 (0.26-120)	58.5 (0.23-153)	30.6 (0.22-153)	0.56 (0.04-2.54)	0.93 (0.02-5.29)	0.66 (0.02-3.55)
LFBC	XI	1.31 (0.24-4.08)	1.42 (0.20-5.03)	1.41 (0.19-4.97)	1.13 (0.15-4.19)	1.37 (0.09-6.33)	1.21 (0.10-5.20)
LFBC	XIB	1.36 (0.82-2.13)	1.42 (0.65-2.71)	1.40 (0.64-2.68)	1.14 (0.43-2.50)	1.24 (0.26-3.77)	1.15 (0.28-3.15)
LFBC	XIC	**5.75 (1.48-16.1)**	**6.02 (1.11-19.7)**	**6.12 (1.12-20.1)**	0.65 (0.11-2.19)	0.67 (0.04-3.11)	0.70 (0.06-2.93)
LFBC	XO	1.14 (0.69-1.78)	1.13 (0.51-2.18)	1.15 (0.51-2.22)	0.82 (0.29-1.86)	0.82 (0.14-2.66)	0.89 (0.19-2.60)
LFBC	XOB	1.24 (0.72-1.99)	1.28 (0.58-2.50)	1.28 (0.57-2.49)	0.78 (0.27-1.81)	0.85 (0.16-2.71)	0.80 (0.18-2.30)
LFBC	XOC	**6.86 (1.98-18.1)**	**7.10 (1.57-21.4**	**7.20 (1.59-21.8)**	1.08 (0.21-3.39)	1.25 (0.11-5.35)	1.27 (0.15-4.92)
LFBC	XOBC	1.39 (0.74-2.37)	1.45 (0.50-3.32)	1.45 (0.50-3.31)	0.87 (0.24-2.30)	0.95 (0.11-3.59)	0.89 (0.14-3.03)
LFBC	X	0.42 (0.18-0.86)	0.47 (0.14-1.17)	0.47 (0.14-1.18)	**0.29 (0.08-0.79)**	0.34 (0.04-1.30)	0.33 (0.05-1.11)
LFBC	C	0.61 (0.20-1.43)	0.76 (0.15-2.42)	0.75 (0.14-2.37)	**0.18 (0.06-0.46)**	0.28 (0.03-1.15)	**0.21 (0.03-0.77)**
LFBC	I	**0.36 (0.18-0.66)**	0.46 (0.15-1.09)	0.44 (0.15-1.06)	**0.23 (0.08-0.54)**	**0.29 (0.05-0.98)**	**0.23 (0.05-0.71)**
LFBC	O	**0.33 (0.09-0.82)**	0.37 (0.08-1.07)	0.38 (0.08-1.10)	**0.33 (0.11-0.76)**	0.34 (0.05-1.16)	0.38 (0.07-1.15)
LFBC	P	0.71 (0.22-1.71)	0.92 (0.14-3.19)	0.91 (0.14-3.14)	**0.19 (0.05-0.48)**	0.31 (0.02-1.40)	**0.23 (0.02-0.90)**
LFP	CB	0.48 (0.09-1.53)	0.68 (0.07-2.80)	0.66 (0.06-2.74)	-	-	-
LFP	CI	1.11 (0.43-2.38)	1.29 (0.30-3.73)	1.27 (0.29-3.65)	-	-	-
LFP	CIB	1.34 (0.43-3.17)	1.78 (0.32-5.89)	1.76 (0.31-5.80)	-	-	-
LFP	IO	0.70 (0.37-1.23)	0.69 (0.26-1.51)	0.71 (0.26-1.55)	-	-	-
LFP	IOB	0.88 (0.31-2.01)	0.85 (0.20-2.39)	0.85 (0.20-2.39)	-	-	-
LFP	IP	0.94 (0.36-2.02)	1.08 (0.25-3.11)	1.06 (0.24-3.05)	-	-	-
LFP	FI	0.55 (0.25-1.05)	0.53 (0.15-1.32)	0.56 (0.16-1.42)	-	-	-

T1	T2	Overall response rate			Disease control rate		
		Fixed effects	Random effects	Meta-regression	Fixed effects	Random effects	Meta-regression
LFP	XB	**0.48 (0.20-0.96)**	0.48 (0.14-1.21)	0.48 (0.14-1.21)	-	-	-
LFP	XC	23.0 (0.16-77.3)	34.2 (0.12-87.3)	17.0 (0.12-89.3)	-	-	-
LFP	XI	0.85 (0.14-2.75)	0.83 (0.10-3.08)	0.83 (0.10-3.05)	-	-	-
LFP	XIB	0.88 (0.45-1.57)	0.83 (0.30-1.80)	0.82 (0.30-1.80)	-	-	-
LFP	XIC	3.71 (0.88-10.7)	3.50 (0.57-12.0)	3.57 (0.57-12.1)	-	-	-
LFP	XO	0.73 (0.38-1.27)	0.65 (0.25-1.38)	0.67 (0.25-1.42)	-	-	-
LFP	XOB	0.80 (0.39-1.48)	0.75 (0.26-1.68)	0.75 (0.26-1.70)	-	-	-
LFP	XOC	**4.42 (1.18-12.2)**	4.12 (0.80-13.1)	4.20 (0.81-13.3)	-	-	-
LFP	XOBC	0.90 (0.41-1.72)	0.85 (0.24-2.14)	0.85 (0.24-2.18)	-	-	-
LFP	X	**0.27 (0.10-0.59)**	**0.27 (0.07-0.73)**	**0.27 (0.07-0.73)**	-	-	-
LFP	C	**0.39 (0.12-0.97)**	0.44 (0.07-1.46)	0.43 (0.07-1.42)	-	-	-
LFP	I	**0.24 (0.10-0.46)**	**0.26 (0.08-0.67)**	**0.26 (0.07-0.65)**	-	-	-
LFP	O	**0.21 (0.05-0.55)**	**0.22 (0.04-0.65)**	**0.22 (0.04-0.67)**	-	-	-
LFP	P	0.46 (0.13-1.16)	0.53 (0.07-1.92)	0.53 (0.07-1.86)	-	-	-
CB	CI	3.28 (0.88-8.89)	3.16 (0.62-9.94)	3.17 (0.62-9.96)	-	-	-
CB	CIB	**3.66 (1.16-9.18)**	3.80 (0.98-10.6)	3.82 (0.99-10.6)	-	-	-
CB	IO	2.28 (0.50-6.82)	2.11 (0.27-7.81)	2.19 (0.29-8.06)	-	-	-
CB	IOB	2.90 (0.47-9.95)	2.71 (0.23-11.7)	2.75 (0.23-11.8)	-	-	-
CB	IP	2.85 (0.66-8.32)	2.82 (0.43-9.86)	2.81 (0.43-9.84)	-	-	-
CB	FI	1.80 (0.35-5.73)	1.67 (0.17-6.74)	1.80 (0.18-7.31)	-	-	-
CB	XB	1.57 (0.29-5.09)	1.50 (0.15-6.20)	1.54 (0.15-6.29)	-	-	-
CB	XC	85.3 (0.38-230)	79.4 (0.26-197)	42.1 (0.27-227)	-	-	-
CB	XI	2.78 (0.26-11.6)	2.61 (0.13-13.0)	2.66 (0.13-13.1)	-	-	-
CB	XIB	2.89 (0.59-8.94)	2.62 (0.3-10.19)	2.64 (0.30-10.2)	-	-	-
CB	XIC	**12.2 (1.47-47.2)**	11.1 (0.70-52.3)	11.5 (0.72-54.0)	-	-	-
CB	XO	2.40 (0.50-7.41)	2.06 (0.24-7.91)	2.14 (0.25-8.24)	-	-	-
CB	XOB	2.64 (0.53-8.29)	2.39 (0.26-9.33)	2.43 (0.27-9.51)	-	-	-
CB	XOC	**14.5 (1.91-54.5)**	13.1 (0.95-59.2)	13.5 (0.97-61.4)	-	-	-
CB	XOBC	2.95 (0.57-9.42)	2.68 (0.26-11.0)	2.75 (0.26-11.4)	-	-	-
CB	X	0.89 (0.15-3.00)	0.86 (0.08-3.59)	0.88 (0.08-3.70)	-	-	-
CB	C	1.16 (0.25-3.47)	1.08 (0.16-3.79)	1.09 (0.16-3.82)	-	-	-
CB	I	0.72 (0.18-2.05)	0.71 (0.12-2.44)	0.71 (0.11-2.41)	-	-	-
CB	O	0.71 (0.09-2.59)	0.68 (0.05-3.02)	0.71 (0.05-3.15)	-	-	-
CB	P	1.34 (0.28-4.09)	1.30 (0.16-4.89)	1.31 (0.16-4.96)	-	-	-
CI	CIB	1.20 (0.69-1.95)	1.37 (0.63-2.67)	1.38 (0.63-2.68)	1.14 (0.58-2.03)	1.30 (0.48-2.90)	1.25 (0.51-2.62)
CI	IO	0.70 (0.36-1.22)	0.67 (0.23-1.51)	0.69 (0.24-1.57)	1.25 (0.84-1.79)	1.18 (0.35-2.90)	1.38 (0.49-3.00)
CI	IOB	0.88 (0.29-2.10)	0.85 (0.16-2.66)	0.87 (0.16-2.70)	0.92 (0.20-2.58)	1.12 (0.11-4.58)	1.25 (0.15-4.65)
CI	IP	0.87 (0.50-1.41)	0.89 (0.40-1.72)	0.89 (0.40-1.72)	0.72 (0.48-1.04)	0.75 (0.28-1.61)	0.73 (0.32-1.45)
CI	FI	0.55 (0.24-1.10)	0.53 (0.13-1.45)	0.57 (0.14-1.59)	1.42 (0.66-2.69)	1.31 (0.22-4.22)	2.02 (0.42-5.82)
CI	XB	0.48 (0.19-1.01)	0.48 (0.11-1.35)	0.49 (0.11-1.38)	1.42 (0.51-3.13)	1.55 (0.23-5.39)	1.75 (0.33-5.41)

T1	T2	Overall response rate			Disease control rate		
		Fixed effects	Random effects	Meta-regression	Fixed effects	Random effects	Meta-regression
CI	XC	29.3 (0.18-69.3)	23.5 (0.15-64.4)	14.2 (0.16-72.1)	1.24 (0.13-5.06)	1.53 (0.11-7.12)	1.40 (0.12-6.32)
CI	XI	0.85 (0.13-2.82)	0.83 (0.09-3.27)	0.84 (0.09-3.28)	2.73 (0.44-9.40)	3.20 (0.23-14.5)	3.42 (0.31-14.4)
CI	XIB	0.88 (0.41-1.68)	0.82 (0.24-2.09)	0.83 (0.24-2.09)	**2.74 (1.40-4.86)**	2.94 (0.64-8.60)	3.22 (0.88-8.46)
CI	XIC	3.72 (0.83-11.0)	3.51 (0.47-12.9)	3.64 (0.49-13.4)	1.53 (0.32-4.64)	1.54 (0.12-6.85)	1.93 (0.19-7.66)
CI	XO	0.73 (0.35-1.37)	0.65 (0.19-1.61)	0.68 (0.20-1.68)	**1.93 (1.01-3.37)**	1.86 (0.41-5.39)	2.44 (0.64-6.36)
CI	XOB	0.80 (0.36-1.58)	0.75 (0.21-1.95)	0.77 (0.21-1.99)	1.90 (0.81-3.79)	2.06 (0.37-6.65)	2.28 (0.52-6.56)
CI	XOC	**4.43 (1.11-12.5)**	4.14 (0.66-14.4)	4.28 (0.69-14.8)	2.56 (0.64-7.06)	2.85 (0.30-11.6)	3.49 (0.47-12.7)
CI	XOBC	0.90 (0.38-1.84)	0.85 (0.19-2.41)	0.87 (0.19-2.47)	2.10 (0.69-4.98)	2.28 (0.26-8.66)	2.56 (0.40-8.75)
CI	X	**0.27 (0.09-0.62)**	0.27 (0.06-0.81)	0.28 (0.06-0.83)	0.70 (0.24-1.61)	0.79 (0.11-2.82)	0.90 (0.16-2.90)
CI	C	**0.35 (0.18-0.62)**	0.34 (0.14-0.66)	0.34 (0.14-0.67)	**0.41 (0.26-0.62)**	**0.46 (0.21-0.90)**	**0.45 (0.22-0.82)**
CI	I	**0.22 (0.14-0.32)**	0.23 (0.11-0.40)	0.22 (0.11-0.40)	**0.53 (0.43-0.65)**	0.56 (0.24-1.09)	0.55 (0.28-0.97)
CI	O	**0.22 (0.05-0.57)**	0.21 (0.03-0.71)	0.22 (0.03-0.74)	0.77 (0.39-1.39)	0.76 (0.14-2.38)	1.02 (0.24-2.80)
CI	P	**0.41 (0.19-0.76)**	0.41 (0.13-0.97)	0.41 (0.13-0.99)	**0.42 (0.24-0.67)**	0.51 (0.14-1.38)	0.48 (0.16-1.15)
CIB	IO	0.62 (0.26-1.28)	0.56 (0.14-1.49)	0.57 (0.14-1.55)	1.21 (0.54-2.35)	1.12 (0.21-3.44)	1.31 (0.31-3.58)
CIB	IOB	0.79 (0.22-2.07)	0.71 (0.10-2.44)	0.72 (0.11-2.51)	0.90 (0.16-2.79)	1.07 (0.07-4.79)	1.19 (0.11-4.92)
CIB	IP	0.78 (0.35-1.52)	0.74 (0.23-1.78)	0.74 (0.23-1.79)	0.70 (0.31-1.35)	0.71 (0.16-2.00)	0.70 (0.19-1.79)
CIB	FI	0.49 (0.17-1.12)	0.44 (0.08-1.36)	0.47 (0.08-1.49)	1.38 (0.48-3.15)	1.24 (0.15-4.62)	1.92 (0.28-6.43)
CIB	XB	0.43 (0.14-1.01)	0.40 (0.07-1.26)	0.40 (0.07-1.30)	1.38 (0.39-3.51)	1.47 (0.15-5.80)	1.66 (0.23-5.91)
CIB	XC	26.2 (0.15-62.5)	19.5 (0.11-52.8)	11.5 (0.11-60.7)	1.21 (0.11-5.18)	1.44 (0.08-7.19)	1.32 (0.09-6.36)
CIB	XI	0.76 (0.11-2.66)	0.69 (0.06-2.91)	0.70 (0.06-2.95)	2.65 (0.36-9.81)	3.03 (0.16-14.8)	3.25 (0.23-14.7)
CIB	XIB	0.79 (0.30-1.72)	0.69 (0.15-1.99)	0.69 (0.15-2.03)	2.66 (1.00-5.80)	2.79 (0.41-9.72)	3.06 (0.58-9.58)
CIB	XIC	3.32 (0.65-10.5)	2.91 (0.31-11.5)	3.02 (0.32-12.0)	1.48 (0.26-4.92)	1.46 (0.08-7.03)	1.84 (0.14-7.85)
CIB	XO	0.66 (0.25-1.41)	0.54 (0.12-1.54)	0.56 (0.12-1.62)	1.87 (0.71-4.05)	1.76 (0.26-6.06)	2.31 (0.42-7.14)
CIB	XOB	0.72 (0.26-1.61)	0.63 (0.13-1.86)	0.64 (0.13-1.90)	1.84 (0.60-4.37)	1.95 (0.24-7.32)	2.17 (0.35-7.26)
CIB	XOC	3.96 (0.86-12.0)	3.43 (0.43-12.9)	3.55 (0.44-13.3)	2.48 (0.51-7.61)	2.71 (0.2-12.17)	3.32 (0.33-13.2)
CIB	XOBC	0.80 (0.28-1.86)	0.73 (0.12-2.25)	0.72 (0.12-2.33)	2.04 (0.53-5.52)	2.16 (0.18-9.15)	2.43 (0.28-9.31)
CIB	X	**0.24 (0.07-0.62)**	**0.23 (0.04-0.74)**	**0.23 (0.04-0.77)**	0.68 (0.18-1.79)	0.74 (0.07-3.00)	0.86 (0.11-3.14)
CIB	C	**0.32 (0.13-0.65)**	**0.28 (0.08-0.69)**	**0.28 (0.08-0.69)**	0.40 (0.17-0.80)	0.44 (0.11-1.18)	0.42 (0.13-1.05)
CIB	I	**0.20 (0.10-0.36)**	**0.19 (0.06-0.43)**	**0.19 (0.06-0.43)**	0.52 (0.25-0.95)	0.53 (0.13-1.41)	0.52 (0.16-1.26)
CIB	O	**0.19 (0.04-0.55)**	**0.18 (0.02-0.65)**	**0.19 (0.02-0.68)**	0.75 (0.28-1.65)	0.72 (0.09-2.62)	0.97 (0.16-3.11)
CIB	P	**0.37 (0.14-0.78)**	**0.34 (0.08-0.94)**	**0.34 (0.08-0.96)**	**0.41 (0.17-0.84)**	0.48 (0.09-1.59)	0.45 (0.10-1.32)
IO	IOB	1.27 (0.53-2.61)	1.29 (0.38-3.23)	1.27 (0.37-3.19)	0.74 (0.17-2.02)	1.00 (0.13-3.62)	0.93 (0.14-3.16)
IO	IP	1.34 (0.68-2.38)	1.56 (0.55-3.59)	1.51 (0.52-3.48)	**0.59 (0.36-0.91)**	0.77 (0.23-1.94)	0.61 (0.22-1.40)
IO	FI	0.79 (0.47-1.25)	0.80 (0.32-1.65)	0.83 (0.33-1.73)	1.14 (0.58-2.04)	1.16 (0.29-3.12)	1.50 (0.45-3.66)
IO	XB	0.69 (0.37-1.19)	0.72 (0.27-1.56)	0.71 (0.27-1.54)	1.14 (0.44-2.41)	1.38 (0.29-4.09)	1.30 (0.34-3.54)
IO	XC	37.8 (0.26-109)	47.7 (0.22-128)	25.1 (0.21-129)	1.03 (0.10-4.30)	1.74 (0.08-8.97)	1.25 (0.08-6.13)
IO	XI	1.22 (0.23-3.74)	1.25 (0.18-4.32)	1.23 (0.18-4.23)	2.20 (0.37-7.44)	2.84 (0.27-11.9)	2.55 (0.29-9.93)
IO	XIB	1.27 (0.86-1.82)	1.25 (0.64-2.18)	1.22 (0.62-2.13)	**2.21 (1.25-3.66)**	2.60 (0.89-6.07)	2.41 (0.95-5.22)
IO	XIC	**5.36 (1.46-14.6)**	**5.28 (1.06-16.6)**	**5.30 (1.05-16.7)**	1.24 (0.27-3.66)	1.36 (0.14-5.48)	1.43 (0.19-5.25)
IO	XO	1.06 (0.75-1.45)	0.98 (0.53-1.65)	0.99 (0.53-1.66)	1.56 (0.90-2.52)	1.64 (0.56-3.78)	1.81 (0.71-3.85)

T1	T2	Overall response rate			Disease control rate		
		Fixed effects	Random effects	Meta-regression	Fixed effects	Random effects	Meta-regression
IO	XOB	1.16 (0.73-1.75)	1.14 (0.55-2.09)	1.12 (0.53-2.07)	1.53 (0.70-2.90)	1.82 (0.49-4.83)	1.71 (0.54-4.21)
IO	XOC	**6.39 (1.97-16.4)**	**6.23 (1.51-17.9)**	**6.24 (1.51-18.0)**	2.06 (0.54-5.54)	2.51 (0.36-9.07)	2.59 (0.46-8.53)
IO	XOBC	1.30 (0.74-2.11)	1.28 (0.47-2.79)	1.26 (0.46-2.77)	1.70 (0.59-3.85)	2.03 (0.33-6.81)	1.91 (0.40-5.88)
IO	X	**0.39 (0.18-0.76)**	**0.41 (0.14-0.96)**	**0.41 (0.13-0.95)**	0.57 (0.20-1.25)	0.70 (0.14-2.15)	0.67 (0.16-1.92)
IO	C	0.56 (0.21-1.20)	0.64 (0.16-1.78)	0.62 (0.15-1.70)	**0.34 (0.19-0.58)**	0.52 (0.12-1.55)	0.40 (0.11-1.09)
IO	I	**0.33 (0.20-0.51)**	**0.38 (0.18-0.72)**	**0.37 (0.17-0.69)**	**0.44 (0.32-0.59)**	0.54 (0.24-1.06)	**0.44 (0.22-0.81)**
IO	O	**0.31 (0.09-0.72)**	**0.32 (0.07-0.87)**	**0.33 (0.07-0.88)**	0.62 (0.35-1.04)	0.67 (0.19-1.72)	0.76 (0.26-1.75)
IO	P	0.65 (0.23-1.44)	0.77 (0.15-2.40)	0.75 (0.15-2.30)	**0.35 (0.17-0.62)**	0.58 (0.09-2.05)	0.43 (0.09-1.35)
IOB	IP	1.24 (0.39-2.98)	1.62 (0.31-5.11)	1.58 (0.30-5.02)	1.18 (0.26-3.63)	1.53 (0.15-6.46)	1.19 (0.14-4.66)
IOB	FI	0.73 (0.27-1.59)	0.80 (0.19-2.24)	0.84 (0.20-2.36)	2.24 (0.49-6.91)	2.20 (0.22-9.06)	2.78 (0.35-10.5)
IOB	XB	0.63 (0.22-1.42)	0.71 (0.17-1.99)	0.71 (0.17-1.99)	2.21 (0.44-7.12)	2.49 (0.25-10.2)	2.34 (0.30-8.98)
IOB	XC	30.8 (0.19-102)	50.9 (0.16-128)	26.2 (0.16-133)	2.08 (0.12-10.3)	3.45 (0.07-20.6)	2.45 (0.07-13.9)
IOB	XI	1.11 (0.17-3.80)	1.23 (0.13-4.81)	1.22 (0.13-4.75)	4.23 (0.43-17.6)	5.10 (0.27-25.2)	4.52 (0.30-21.0)
IOB	XIB	1.16 (0.48-2.37)	1.23 (0.38-3.03)	1.22 (0.37-3.01)	**4.25 (1.08-12.3)**	4.65 (0.70-16.9)	4.27 (0.77-14.4)
IOB	XIC	**4.89 (1.01-15.1)**	5.23 (0.74-19.0)	5.32 (0.75-19.5)	2.41 (0.29-9.34)	2.51 (0.13-12.5)	2.62 (0.18-12.0)
IOB	XO	0.97 (0.40-1.99)	0.98 (0.29-2.43)	1.00 (0.30-2.48)	3.04 (0.74-9.06)	3.07 (0.40-11.6)	3.33 (0.52-11.7)
IOB	XOB	1.05 (0.42-2.19)	1.11 (0.34-2.78)	1.11 (0.33-2.78)	2.91 (0.69-8.82)	3.18 (0.44-11.9)	2.97 (0.49-10.4)
IOB	XOC	**5.83 (1.34-17.2)**	**6.16 (1.03-21.0)**	**6.27 (1.04-21.4)**	4.02 (0.57-14.8)	4.66 (0.32-21.8)	4.72 (0.42-20.5)
IOB	XOBC	1.18 (0.45-2.54)	1.26 (0.31-3.48)	1.26 (0.30-3.51)	3.24 (0.63-10.6)	3.56 (0.32-15.3)	3.33 (0.38-13.1)
IOB	X	0.36 (0.11-0.87)	0.41 (0.09-1.19)	0.41 (0.09-1.21)	1.09 (0.20-3.60)	1.27 (0.12-5.43)	1.22 (0.14-4.76)
IOB	C	0.52 (0.13-1.40)	0.66 (0.10-2.35)	0.65 (0.10-2.31)	0.69 (0.15-2.18)	1.04 (0.08-4.71)	0.78 (0.08-3.26)
IOB	I	**0.31 (0.11-0.69)**	0.40 (0.09-1.13)	0.38 (0.09-1.10)	0.87 (0.21-2.62)	1.08 (0.13-4.21)	0.86 (0.13-3.11)
IOB	O	**0.28 (0.06-0.79)**	0.32 (0.05-1.07)	0.33 (0.05-1.09)	1.22 (0.29-3.66)	1.26 (0.14-5.03)	1.40 (0.20-5.12)
IOB	P	0.60 (0.15-1.66)	0.80 (0.10-3.07)	0.79 (0.10-3.01)	0.70 (0.14-2.24)	1.16 (0.07-5.68)	0.84 (0.07-3.71)
IP	FI	0.66 (0.28-1.33)	0.63 (0.15-1.75)	0.69 (0.16-1.93)	2.04 (0.90-4.02)	1.95 (0.33-6.36)	2.97 (0.60-8.72)
IP	XB	0.57 (0.22-1.22)	0.57 (0.13-1.63)	0.59 (0.13-1.68)	2.04 (0.70-4.62)	2.31 (0.34-8.14)	2.57 (0.48-8.04)
IP	XC	32.6 (0.21-85.6)	30.0 (0.17-82.8)	18.8 (0.17-93.5)	1.80 (0.18-7.48)	2.49 (0.13-12.4)	2.20 (0.15-10.5)
IP	XI	1.01 (0.16-3.36)	1.00 (0.10-3.97)	1.01 (0.10-4.01)	3.92 (0.61-13.7)	4.77 (0.33-21.8)	5.03 (0.45-21.2)
IP	XIB	1.05 (0.48-2.03)	0.99 (0.28-2.51)	1.01 (0.29-2.56)	**3.93 (1.89-7.31)**	4.37 (0.94-13.0)	**4.73 (1.27-12.6)**
IP	XIC	4.44 (0.98-13.3)	4.22 (0.57-15.5)	4.4 (0.59-16.19)	2.20 (0.44-6.79)	2.29 (0.17-10.2)	2.84 (0.28-11.3)
IP	XO	0.88 (0.41-1.66)	0.78 (0.23-1.94)	0.82 (0.24-2.05)	**2.77 (1.36-5.07)**	2.76 (0.60-8.14)	3.59 (0.92-9.53)
IP	XOB	0.96 (0.42-1.91)	0.91 (0.25-2.34)	0.93 (0.25-2.42)	**2.72 (1.10-5.64)**	3.06 (0.54-9.98)	3.36 (0.75-9.80)
IP	XOC	**5.29 (1.31-15.1)**	4.97 (0.79-17.2)	5.18 (0.82-17.9)	3.67 (0.88-10.4)	4.24 (0.43-17.4)	5.13 (0.68-18.8)
IP	XOBC	1.07 (0.44-2.21)	1.02 (0.23-2.89)	1.05 (0.23-3.02)	3.02 (0.95-7.32)	3.4 (0.39-13.05)	3.76 (0.57-13.0)
IP	X	**0.33 (0.11-0.75)**	**0.33 (0.07-0.97)**	0.34 (0.07-1.01)	1.01 (0.32-2.38)	1.17 (0.16-4.23)	1.33 (0.23-4.35)
IP	C	**0.44 (0.18-0.89)**	0.44 (0.13-1.09)	0.44 (0.13-1.09)	0.60 (0.32-1.03)	0.75 (0.20-2.01)	0.70 (0.23-1.71)
IP	I	**0.26 (0.17-0.39)**	**0.27 (0.14-0.49)**	**0.27 (0.13-0.49)**	0.76 (0.53-1.06)	0.83 (0.35-1.67)	0.80 (0.39-1.48)
IP	O	**0.26 (0.06-0.69)**	**0.26 (0.04-0.85)**	**0.27 (0.04-0.90)**	1.11 (0.53-2.08)	1.13 (0.21-3.58)	1.50 (0.35-4.18)
IP	P	0.50 (0.19-1.08)	0.53 (0.12-1.50)	0.54 (0.12-1.51)	0.61 (0.30-1.09)	0.84 (0.15-2.73)	0.76 (0.18-2.19)
FI	XB	0.92 (0.43-1.72)	1.03 (0.31-2.59)	0.98 (0.29-2.48)	1.08 (0.36-2.47)	1.54 (0.25-5.32)	1.07 (0.22-3.35)

T1	T2	Overall response rate			Disease control rate		
		Fixed effects	Random effects	Meta-regression	Fixed effects	Random effects	Meta-regression
FI	XC	49.5 (0.32-147)	80.8 (0.27-190)	36.6 (0.25-183)	1.00 (0.09-4.34)	2.07 (0.07-11.5)	1.09 (0.05-5.82)
FI	XI	1.62 (0.29-5.11)	1.79 (0.23-6.66)	1.70 (0.21-6.24)	2.07 (0.32-7.29)	3.19 (0.24-14.5)	2.10 (0.20-8.87)
FI	XIB	1.68 (0.96-2.73)	1.79 (0.69-3.82)	1.69 (0.64-3.63)	2.08 (0.99-3.90)	2.91 (0.70-8.35)	1.98 (0.58-5.27)
FI	XIC	**7.09 (1.79-20.1)**	**7.58 (1.27-25.6)**	**7.36 (1.22-25.1)**	1.16 (0.23-3.61)	1.52 (0.12-6.70)	1.17 (0.13-4.65)
FI	XO	1.40 (0.82-2.23)	1.41 (0.56-2.96)	1.37 (0.54-2.88)	1.47 (0.71-2.72)	1.84 (0.44-5.25)	1.48 (0.44-3.81)
FI	XOB	1.53 (0.83-2.61)	1.63 (0.60-3.61)	1.55 (0.56-3.45)	1.44 (0.57-3.02)	2.03 (0.40-6.40)	1.40 (0.34-4.07)
FI	XOC	**8.46 (2.40-22.5)**	**8.93 (1.79-28.1)**	**8.66 (1.73-27.3)**	1.94 (0.46-5.50)	2.82 (0.31-11.3)	2.12 (0.31-7.70)
FI	XOBC	1.72 (0.86-3.08)	1.84 (0.54-4.62)	1.75 (0.51-4.45)	1.60 (0.50-3.91)	2.26 (0.29-8.48)	1.57 (0.26-5.35)
FI	X	0.52 (0.21-1.08)	0.59 (0.16-1.56)	0.56 (0.15-1.51)	0.53 (0.17-1.27)	0.78 (0.12-2.78)	0.55 (0.10-1.80)
FI	C	0.75 (0.24-1.76)	0.95 (0.17-3.09)	0.89 (0.16-2.87)	**0.33 (0.13-0.69)**	0.62 (0.09-2.33)	0.35 (0.06-1.20)
FI	I	**0.45 (0.22-0.81)**	0.57 (0.18-1.42)	0.53 (0.16-1.32)	**0.42 (0.20-0.77)**	0.64 (0.15-1.86)	0.38 (0.10-1.05)
FI	O	0.41 (0.11-1.01)	0.47 (0.09-1.38)	0.45 (0.08-1.36)	0.59 (0.28-1.11)	0.75 (0.16-2.30)	0.62 (0.16-1.68)
FI	P	0.87 (0.27-2.11)	1.16 (0.17-4.09)	1.08 (0.16-3.80)	0.34 (0.13-0.72)	0.69 (0.07-2.91)	0.38 (0.05-1.43)
XB	XC	52.6 (0.36-173)	88.9 (0.30-216)	42.1 (0.29-214)	1.09 (0.08-4.95)	1.89 (0.06-10.6)	1.33 (0.06-7.08)
XB	XI	1.87 (0.34-5.85)	1.95 (0.27-6.86)	1.92 (0.27-6.70)	2.18 (0.33-7.72)	2.64 (0.24-11.3)	2.38 (0.26-9.50)
XB	XIB	1.94 (1.17-3.05)	1.94 (0.91-3.64)	1.92 (0.90-3.60)	**2.19 (1.02-4.31)**	2.42 (0.76-6.00)	2.25 (0.81-5.19)
XB	XIC	**8.30 (2.03-23.7)**	**8.44 (1.45-28.1)**	**8.60 (1.47-29.0)**	1.25 (0.23-4.12)	1.32 (0.11-5.71)	1.40 (0.15-5.52)
XB	XO	1.65 (0.89-2.81)	1.58 (0.64-3.27)	1.61 (0.65-3.34)	1.58 (0.64-3.36)	1.60 (0.40-4.46)	1.77 (0.51-4.53)
XB	XOB	1.79 (0.96-3.05)	1.80 (0.72-3.75)	1.80 (0.72-3.75)	1.53 (0.57-3.39)	1.72 (0.40-5.01)	1.62 (0.44-4.34)
XB	XOC	**9.90 (2.72-26.8)**	**9.94 (2.05-30.6)**	**10.1 (2.08-31.4)**	2.09 (0.45-6.34)	2.44 (0.29-9.59)	2.53 (0.36-9.14)
XB	XOBC	2.00 (0.99-3.60)	2.03 (0.64-4.88)	2.03 (0.64-4.88)	1.70 (0.50-4.33)	1.92 (0.28-6.84)	1.81 (0.33-5.84)
XB	X	0.58 (0.30-1.00)	0.60 (0.26-1.20)	0.61 (0.26-1.22)	**0.50 (0.30-0.79)**	0.55 (0.20-1.21)	0.55 (0.23-1.11)
XB	C	0.88 (0.27-2.17)	1.08 (0.19-3.53)	1.06 (0.18-3.49)	**0.36 (0.12-0.87)**	0.57 (0.07-2.22)	0.43 (0.07-1.49)
XB	I	0.53 (0.23-1.03)	0.64 (0.19-1.63)	0.63 (0.18-1.59)	0.46 (0.17-1.01)	0.59 (0.12-1.85)	0.47 (0.11-1.35)
XB	O	0.48 (0.12-1.23)	0.53 (0.10-1.60)	0.54 (0.10-1.65)	0.64 (0.24-1.43)	0.69 (0.13-2.20)	0.76 (0.17-2.19)
XB	P	1.03 (0.29-2.60)	1.31 (0.18-4.66)	1.28 (0.18-4.59)	**0.37 (0.11-0.91)**	0.63 (0.05-2.74)	0.46 (0.06-1.76)
XC	XI	0.88 (0.01-5.33)	0.97 (0.01-6.25)	0.97 (0.01-6.24)	5.19 (0.23-26.7)	6.63 (0.11-39.9)	6.96 (0.16-39.5)
XC	XIB	0.92 (0.01-5.04)	0.97 (0.01-5.64)	0.97 (0.01-5.76)	5.23 (0.46-21.8)	6.09 (0.23-32.0)	6.46 (0.33-31.4)
XC	XIC	3.92 (0.03-23.1)	4.13 (0.02-25.8)	4.24 (0.03-26.6)	2.93 (0.16-14.4)	3.20 (0.06-19.2)	3.96 (0.10-22.3)
XC	XO	0.77 (0.01-4.18)	0.77 (0.01-4.41)	0.79 (0.01-4.64)	3.69 (0.33-15.4)	3.85 (0.15-20.1)	4.90 (0.24-23.8)
XC	XOB	0.84 (0.01-4.58)	0.89 (0.01-5.13)	0.89 (0.01-5.33)	3.62 (0.30-15.5)	4.26 (0.14-23.1)	4.57 (0.21-22.9)
XC	XOC	4.67 (0.04-27.2)	4.87 (0.03-29.9)	4.97 (0.03-30.5)	4.89 (0.30-23.1)	5.94 (0.13-34.6)	7.08 (0.22-38.8)
XC	XOBC	0.94 (0.01-5.19)	1.00 (0.01-5.90)	1.01 (0.01-6.13)	4.02 (0.29-18.0)	4.7 (0.12-26.89)	5.12 (0.18-27.3)
XC	X	0.29 (0.00-1.60)	0.32 (0.00-1.91)	0.33 (0.00-2.00)	1.34 (0.10-5.97)	1.64 (0.05-9.15)	1.82 (0.07-9.47)
XC	C	0.33 (0.01-1.67)	0.35 (0.01-1.82)	0.34 (0.00-1.77)	0.75 (0.08-2.88)	0.82 (0.07-3.45)	0.80 (0.08-3.18)
XC	I	0.23 (0.00-1.22)	0.27 (0.00-1.50)	0.26 (0.00-1.48)	1.01 (0.10-4.02)	1.14 (0.06-5.48)	1.09 (0.08-4.85)
XC	O	0.22 (0.00-1.31)	0.25 (0.00-1.56)	0.26 (0.00-1.63)	1.48 (0.13-6.16)	1.58 (0.05-8.44)	2.06 (0.10-10.1)
XC	P	0.39 (0.01-1.96)	0.42 (0.01-2.27)	0.41 (0.01-2.25)	0.76 (0.08-2.94)	0.91 (0.06-4.19)	0.86 (0.07-3.68)
XI	XIB	1.67 (0.35-5.19)	1.74 (0.31-5.79)	1.73 (0.31-5.81)	1.67 (0.32-5.20)	1.83 (0.25-6.57)	1.76 (0.27-5.97)
XI	XIC	7.13 (0.83-28.4)	7.69 (0.63-34.1)	7.95 (0.64-35.2)	0.98 (0.09-4.14)	1.07 (0.04-5.57)	1.14 (0.06-5.52)

T1	T2	Overall response rate			Disease control rate		
		Fixed effects	Random effects	Meta-regression	Fixed effects	Random effects	Meta-regression
XI	XO	1.42 (0.28-4.55)	1.44 (0.22-5.10)	1.48 (0.23-5.29)	1.23 (0.20-4.09)	1.30 (0.12-5.37)	1.45 (0.17-5.60)
XI	XOB	1.53 (0.31-4.91)	1.62 (0.26-5.64)	1.63 (0.26-5.68)	1.17 (0.19-3.85)	1.32 (0.14-5.21)	1.27 (0.16-4.72)
XI	XOC	**8.50 (1.08-32.7)**	9.05 (0.86-38.4)	9.34 (0.88-39.8)	1.63 (0.17-6.55)	1.98 (0.11-9.81)	2.06 (0.14-9.46)
XI	XOBC	1.71 (0.33-5.58)	1.83 (0.25-6.77)	1.84 (0.25-6.90)	1.30 (0.18-4.62)	1.47 (0.11-6.59)	1.43 (0.13-5.94)
XI	X	0.52 (0.09-1.78)	0.59 (0.07-2.23)	0.59 (0.08-2.27)	0.44 (0.06-1.54)	0.52 (0.04-2.28)	0.51 (0.05-2.10)
XI	C	0.76 (0.10-2.81)	0.98 (0.08-4.30)	0.96 (0.08-4.26)	**0.28 (0.04-0.97)**	0.45 (0.03-2.18)	0.34 (0.03-1.52)
XI	I	0.45 (0.08-1.52)	0.58 (0.07-2.25)	0.57 (0.07-2.20)	0.35 (0.06-1.19)	0.46 (0.04-1.98)	0.38 (0.04-1.50)
XI	O	0.42 (0.05-1.58)	0.48 (0.04-2.00)	0.50 (0.04-2.12)	0.50 (0.08-1.67)	0.54 (0.04-2.33)	0.61 (0.06-2.45)
XI	P	0.88 (0.12-3.30)	1.19 (0.08-5.49)	1.17 (0.08-5.40)	0.28 (0.04-1.00)	0.50 (0.02-2.57)	0.37 (0.02-1.74)
XIB	XIC	**4.29 (1.18-11.6)**	4.41 (0.91-13.7)	4.54 (0.93-14.2)	0.58 (0.13-1.74)	0.58 (0.06-2.33)	0.65 (0.09-2.34)
XIB	XO	0.85 (0.57-1.22)	0.83 (0.45-1.41)	0.85 (0.46-1.46)	0.74 (0.40-1.24)	0.71 (0.24-1.63)	0.83 (0.32-1.73)
XIB	XOB	0.92 (0.64-1.28)	0.93 (0.56-1.46)	0.94 (0.56-1.48)	0.70 (0.39-1.14)	0.72 (0.30-1.44)	0.72 (0.34-1.37)
XIB	XOC	**5.12 (1.59-13.1)**	**5.20 (1.31-14.8)**	**5.34 (1.33-15.2)**	0.97 (0.25-2.64)	1.08 (0.16-3.84)	1.17 (0.21-3.81)
XIB	XOBC	1.03 (0.64-1.56)	1.05 (0.45-2.09)	1.06 (0.45-2.13)	0.78 (0.31-1.61)	0.80 (0.18-2.30)	0.81 (0.22-2.11)
XIB	X	**0.31 (0.15-0.58)**	**0.34 (0.13-0.74)**	**0.34 (0.13-0.75)**	**0.26 (0.10-0.53)**	**0.28 (0.07-0.77)**	**0.29 (0.09-0.73)**
XIB	C	0.46 (0.16-1.03)	0.56 (0.12-1.69)	0.56 (0.12-1.66)	**0.17 (0.07-0.33)**	**0.24 (0.04-0.82)**	**0.19 (0.04-0.59)**
XIB	I	**0.27 (0.14-0.46)**	0.34 (0.13-0.74)	**0.33 (0.12-0.72)**	**0.21 (0.11-0.36)**	**0.25 (0.08-0.63)**	**0.21 (0.07-0.49)**
XIB	O	**0.25 (0.07-0.59)**	0.27 (0.06-0.75)	**0.28 (0.06-0.78)**	**0.30 (0.15-0.52)**	**0.29 (0.08-0.77)**	**0.35 (0.11-0.82)**
XIB	P	0.53 (0.17-1.24)	0.68 (0.11-2.26)	0.67 (0.12-2.23)	**0.17 (0.07-0.35)**	0.27 (0.03-1.05)	**0.21 (0.03-0.71)**
XIC	XO	**0.27 (0.07-0.69)**	0.28 (0.06-0.81)	**0.28 (0.06-0.82)**	1.84 (0.45-5.08)	2.31 (0.34-8.12)	2.17 (0.39-7.10)
XIC	XOB	**0.30 (0.08-0.77)**	0.33 (0.07-0.98)	**0.32 (0.07-0.97)**	1.86 (0.36-5.80)	2.78 (0.27-11.6)	2.19 (0.27-8.41)
XIC	XOC	1.26 (0.79-1.91)	1.29 (0.67-2.26)	1.30 (0.68-2.27)	1.83 (0.96-3.21)	2.27 (0.86-5.07)	2.12 (0.90-4.43)
XIC	XOBC	**0.33 (0.09-0.87)**	0.36 (0.07-1.08)	0.35 (0.07-1.08)	2.05 (0.34-6.84)	3.02 (0.21-13.8)	2.40 (0.23-10.0)
XIC	X	**0.10 (0.02-0.29)**	**0.12 (0.02-0.40)**	**0.12 (0.02-0.40)**	0.68 (0.12-2.23)	1.03 (0.09-4.40)	0.85 (0.09-3.40)
XIC	C	**0.15 (0.03-0.47)**	**0.20 (0.02-0.79)**	**0.19 (0.02-0.75)**	0.43 (0.08-1.36)	0.88 (0.05-4.18)	0.56 (0.05-2.47)
XIC	I	**0.09 (0.02-0.24)**	**0.12 (0.02-0.40)**	**0.11 (0.02-0.38)**	0.55 (0.12-1.64)	0.92 (0.09-3.80)	0.62 (0.08-2.37)
XIC	O	**0.08 (0.01-0.26)**	**0.10 (0.01-0.36)**	**0.10 (0.01-0.36)**	0.76 (0.16-2.26)	1.05 (0.10-4.35)	0.98 (0.12-3.71)
XIC	P	**0.17 (0.03-0.55)**	0.24 (0.02-1.02)	**0.23 (0.02-0.97)**	0.44 (0.08-1.40)	0.99 (0.05-4.98)	0.61 (0.04-2.82)
XO	XOB	1.11 (0.70-1.68)	1.20 (0.61-2.15)	1.18 (0.59-2.12)	1.02 (0.46-1.96)	1.24 (0.34-3.27)	1.03 (0.33-2.51)
XO	XOC	**6.07 (1.97-15.2)**	**6.39 (1.78-17.2)**	**6.39 (1.78-17.2)**	1.33 (0.41-3.29)	1.54 (0.33-4.61)	1.44 (0.35-4.05)
XO	XOBC	1.24 (0.72-2.02)	1.35 (0.52-2.86)	1.32 (0.51-2.82)	1.13 (0.39-2.58)	1.37 (0.23-4.57)	1.15 (0.24-3.45)
XO	X	**0.38 (0.17-0.70)**	**0.42 (0.16-0.92)**	**0.42 (0.16-0.91)**	**0.37 (0.14-0.79)**	0.45 (0.11-1.22)	0.39 (0.11-1.01)
XO	C	0.54 (0.19-1.21)	0.70 (0.15-2.10)	0.68 (0.14-2.01)	**0.24 (0.10-0.46)**	0.38 (0.07-1.30)	**0.26 (0.06-0.80)**
XO	I	**0.33 (0.18-0.54)**	**0.42 (0.16-0.91)**	**0.40 (0.16-0.87)**	**0.30 (0.16-0.50)**	**0.40 (0.12-0.99)**	**0.28 (0.10-0.67)**
XO	O	**0.30 (0.08-0.69)**	**0.34 (0.08-0.90)**	**0.34 (0.08-0.92)**	**0.41 (0.23-0.69)**	0.46 (0.14-1.14)	0.45 (0.16-1.02)
XO	P	0.63 (0.21-1.46)	0.85 (0.15-2.80)	0.82 (0.14-2.70)	**0.24 (0.10-0.48)**	0.43 (0.05-1.66)	**0.28 (0.05-0.97)**
XOB	XOC	**5.65 (1.74-14.4)**	**5.73 (1.46-16.2)**	**5.85 (1.49-16.5)**	1.48 (0.35-4.21)	1.68 (0.22-6.31)	1.78 (0.29-6.09)
XOB	XOBC	1.12 (0.83-1.48)	1.13 (0.58-2.00)	1.14 (0.57-2.01)	1.11 (0.57-1.98)	1.13 (0.35-2.70)	1.13 (0.41-2.49)
XOB	X	**0.35 (0.15-0.68)**	**0.38 (0.13-0.88)**	**0.38 (0.13-0.89)**	**0.40 (0.14-0.91)**	0.45 (0.09-1.40)	0.45 (0.11-1.28)
XOB	C	0.51 (0.17-1.17)	0.63 (0.13-1.91)	0.62 (0.12-1.90)	**0.25 (0.10-0.56)**	0.39 (0.05-1.41)	**0.30 (0.06-0.97)**

T1	T2	Overall response rate			Disease control rate		
		Fixed effects	Random effects	Meta-regression	Fixed effects	Random effects	Meta-regression
XOB	I	**0.30 (0.15-0.54)**	**0.37 (0.13-0.85)**	**0.37 (0.13-0.84)**	**0.32 (0.14-0.63)**	0.40 (0.10-1.13)	**0.33 (0.09-0.84)**
XOB	O	**0.28 (0.07-0.67)**	**0.31 (0.07-0.85)**	**0.31 (0.07-0.88)**	**0.45 (0.20-0.90)**	0.47 (0.10-1.36)	0.54 (0.14-1.39)
XOB	P	0.59 (0.18-1.40)	0.76 (0.12-2.56)	0.75 (0.12-2.53)	**0.26 (0.09-0.58)**	0.43 (0.04-1.77)	0.32 (0.05-1.15)
XOC	XOBC	**0.26 (0.08-0.64)**	**0.28 (0.06-0.77)**	**0.27 (0.06-0.75)**	1.12 (0.22-3.44)	1.33 (0.13-5.47)	1.13 (0.14-4.24)
XOC	X	**0.08 (0.02-0.22)**	**0.09 (0.02-0.29)**	**0.09 (0.02-0.28)**	0.37 (0.07-1.12)	0.45 (0.05-1.75)	0.40 (0.05-1.43)
XOC	C	**0.12 (0.02-0.35)**	**0.15 (0.02-0.58)**	**0.15 (0.02-0.55)**	**0.24 (0.05-0.68)**	0.39 (0.03-1.68)	0.26 (0.03-1.04)
XOC	I	**0.07 (0.02-0.18)**	**0.09 (0.02-0.28)**	**0.09 (0.02-0.27)**	**0.30 (0.08-0.81)**	0.40 (0.05-1.48)	**0.29 (0.05-0.98)**
XOC	O	**0.06 (0.01-0.20)**	**0.07 (0.01-0.26)**	**0.07 (0.01-0.26)**	0.42 (0.11-1.12)	0.46 (0.06-1.70)	0.46 (0.08-1.54)
XOC	P	**0.14 (0.03-0.42)**	**0.19 (0.02-0.75)**	**0.18 (0.02-0.71)**	**0.24 (0.05-0.70)**	0.43 (0.03-2.02)	0.28 (0.03-1.20)
XOBC	X	**0.32 (0.13-0.65)**	**0.37 (0.10-0.96)**	**0.37 (0.10-0.98)**	0.40 (0.11-1.03)	0.53 (0.07-1.97)	0.49 (0.08-1.68)
XOBC	C	0.46 (0.15-1.10)	0.61 (0.10-2.00)	0.60 (0.10-2.00)	**0.25 (0.07-0.63)**	0.45 (0.04-1.91)	0.33 (0.04-1.23)
XOBC	I	**0.28 (0.13-0.51)**	**0.36 (0.11-0.94)**	**0.35 (0.10-0.92)**	**0.32 (0.11-0.75)**	0.46 (0.07-1.63)	0.36 (0.07-1.14)
XOBC	O	**0.25 (0.07-0.63)**	**0.30 (0.06-0.91)**	**0.31 (0.06-0.94)**	0.45 (0.15-1.05)	0.54 (0.08-1.94)	0.58 (0.11-1.85)
XOBC	P	0.54 (0.16-1.32)	0.74 (0.10-2.65)	0.73 (0.10-2.63)	**0.26 (0.07-0.66)**	0.50 (0.04-2.33)	0.35 (0.04-1.44)
X	C	1.63 (0.44-4.26)	1.99 (0.31-6.79)	1.93 (0.31-6.57)	0.75 (0.23-1.88)	1.17 (0.13-4.71)	0.86 (0.13-3.11)
X	I	0.97 (0.38-2.08)	1.19 (0.31-3.21)	1.14 (0.30-3.08)	0.95 (0.34-2.19)	1.22 (0.22-3.98)	0.95 (0.21-2.84)
X	O	0.89 (0.20-2.41)	0.97 (0.17-3.05)	0.98 (0.17-3.12)	1.33 (0.46-3.09)	1.42 (0.24-4.70)	1.53 (0.32-4.55)
X	P	1.89 (0.49-5.09)	2.41 (0.31-8.88)	2.34 (0.31-8.66)	0.76 (0.22-1.95)	1.31 (0.11-5.80)	0.92 (0.11-3.63)
C	I	0.69 (0.31-1.36)	0.77 (0.25-1.87)	0.76 (0.25-1.84)	1.35 (0.81-2.13)	1.39 (0.41-3.43)	1.37 (0.49-3.02)
C	O	0.67 (0.13-2.00)	0.74 (0.09-2.73)	0.76 (0.09-2.82)	1.97 (0.85-3.92)	1.91 (0.27-6.58)	2.55 (0.47-7.83)
C	P	1.16 (0.84-1.56)	1.21 (0.59-2.21)	1.21 (0.59-2.22)	1.01 (0.76-1.32)	1.11 (0.41-2.43)	1.07 (0.48-2.08)
I	O	0.98 (0.25-2.47)	0.95 (0.18-2.85)	1.00 (0.18-3.01)	1.46 (0.77-2.52)	1.37 (0.35-3.71)	1.86 (0.55-4.55)
I	P	1.94 (0.79-3.94)	2.02 (0.50-5.48)	2.05 (0.51-5.55)	0.80 (0.44-1.33)	1.07 (0.21-3.33)	0.97 (0.26-2.67)
O	P	2.79 (0.55-9.02)	3.50 (0.37-14.4)	3.41 (0.36-14.0)	0.60 (0.24-1.24)	1.13 (0.12-4.61)	0.70 (0.11-2.56)

X, capecitabine; B, bevacizumab; C, cetuximab; F, 5-fluorouracil; I, irinotecan; L, leucovorin; O, oxaliplatin; P, panitumumab. Bold fonts indicate significant difference.

Supplementary Table S5. Hazard ratios and 95% credible intervals for pairwise estimates of overall survival and progression-free survival.

T1	T2	Overall survival			Progression-free survival		
		Fixed effects	Random effects	Meta-regression	Fixed effects	Random effects	Meta-regression
LFI	LFIB	0.98 (0.83-1.16)	0.92 (0.70-1.17)	0.92 (0.70-1.17)	**0.69 (0.59-0.80)**	**0.64 (0.47-0.87)**	**0.64 (0.47-0.87)**
LFI	LFIC	**0.81 (0.72-0.91)**	**0.75 (0.57-0.93)**	**0.75 (0.57-0.93)**	**0.75 (0.66-0.86)**	**0.69 (0.50-0.93)**	**0.69 (0.50-0.93)**
LFI	LFIP	0.93 (0.82-1.05)	0.92 (0.74-1.13)	0.92 (0.74-1.13)	**0.85 (0.75-0.95)**	0.81 (0.61-1.06)	0.81 (0.61-1.06)
LFI	LFIBP	**0.69 (0.52-0.91)**	**0.67 (0.46-0.94)**	**0.67 (0.46-0.95)**	**0.56 (0.40-0.79)**	**0.55 (0.35-0.87)**	**0.55 (0.35-0.87)**
LFI	LFO	**0.80 (0.71-0.89)**	**0.80 (0.67-0.96)**	**0.80 (0.67-0.97)**	**0.69 (0.61-0.78)**	**0.68 (0.53-0.89)**	**0.68 (0.53-0.89)**
LFI	LFOB	0.97 (0.78-1.19)	0.94 (0.69-1.27)	0.94 (0.69-1.27)	**0.72 (0.60-0.85)**	**0.70 (0.50-0.98)**	**0.70 (0.50-0.98)**
LFI	LFOC	**0.67 (0.56-0.81)**	**0.66 (0.49-0.87)**	**0.66 (0.50-0.87)**	**0.60 (0.50-0.70)**	**0.60 (0.44-0.82)**	**0.60 (0.44-0.82)**
LFI	LFOP	**0.77 (0.65-0.91)**	**0.77 (0.59-0.99)**	**0.77 (0.59-0.99)**	**0.67 (0.58-0.79)**	**0.67 (0.48-0.93)**	**0.67 (0.48-0.94)**
LFI	LFOI	1.14 (0.93-1.39)	1.13 (0.85-1.51)	1.13 (0.85-1.50)	1.23 (1.02-1.48)	1.22 (0.86-1.74)	1.22 (0.86-1.73)
LFI	LFOIB	1.14 (0.88-1.48)	0.99 (0.60-1.45)	0.99 (0.61-1.46)	**0.75 (0.61-0.91)**	**0.63 (0.41-0.94)**	**0.63 (0.41-0.94)**
LFI	LFOIP	-	-	-	1.38 (0.83-2.27)	1.37 (0.66-2.82)	1.37 (0.66-2.79)
LFI	LF	0.96 (0.75-1.24)	0.96 (0.66-1.41)	0.96 (0.66-1.40)	0.84 (0.66-1.07)	0.84 (0.51-1.36)	0.84 (0.52-1.36)
LFI	LFC	0.91 (0.64-1.29)	0.90 (0.53-1.55)	0.90 (0.53-1.55)	0.94 (0.68-1.30)	0.94 (0.47-1.85)	0.94 (0.48-1.87)
LFI	LFP	0.55 (0.26-1.14)	0.54 (0.24-1.21)	0.54 (0.24-1.21)	0.72 (0.46-1.15)	0.72 (0.36-1.44)	0.72 (0.36-1.44)
LFI	IO	0.90 (0.79-1.02)	0.90 (0.71-1.13)	0.90 (0.71-1.14)	0.94 (0.83-1.07)	0.94 (0.69-1.28)	0.94 (0.69-1.28)
LFI	IOB	1.26 (0.82-1.92)	1.22 (0.70-2.14)	1.22 (0.70-2.14)	0.79 (0.54-1.16)	0.77 (0.40-1.46)	0.77 (0.40-1.46)
LFI	XB	**0.43 (0.24-0.79)**	**0.43 (0.20-0.92)**	**0.44 (0.20-0.92)**	0.52 (0.27-1.00)	0.51 (0.20-1.31)	0.51 (0.20-1.30)
LFI	XIB	0.92 (0.75-1.13)	0.87 (0.61-1.20)	0.87 (0.61-1.20)	**0.71 (0.60-0.84)**	0.68 (0.45-1.01)	0.68 (0.45-1.01)
LFI	XO	**0.81 (0.70-0.95)**	0.81 (0.62-1.07)	0.81 (0.62-1.07)	**0.73 (0.63-0.86)**	0.72 (0.51-1.03)	0.72 (0.51-1.03)
LFI	XOB	0.89 (0.71-1.12)	0.84 (0.58-1.17)	0.84 (0.58-1.16)	**0.69 (0.57-0.83)**	0.67 (0.45-1.00)	0.67 (0.45-1.00)
LFI	XOBC	0.78 (0.57-1.05)	0.73 (0.44-1.17)	0.73 (0.44-1.17)	**0.56 (0.44-0.72)**	0.55 (0.30-1.00)	0.55 (0.30-1.00)
LFI	X	**0.55 (0.33-0.91)**	0.55 (0.29-1.02)	0.55 (0.29-1.02)	0.98 (0.54-1.79)	0.96 (0.44-2.15)	0.96 (0.43-2.13)
LFIB	LFIC	**0.83 (0.70-0.98)**	0.81 (0.62-1.06)	0.81 (0.62-1.06)	1.10 (0.95-1.27)	1.07 (0.77-1.47)	1.07 (0.77-1.47)
LFIB	LFIP	0.95 (0.78-1.15)	1.00 (0.76-1.34)	1.00 (0.76-1.34)	**1.23 (1.03-1.48)**	1.26 (0.89-1.77)	1.26 (0.89-1.77)
LFIB	LFIBP	0.70 (0.51-0.98)	0.72 (0.47-1.13)	0.73 (0.47-1.13)	0.82 (0.57-1.19)	0.86 (0.50-1.49)	0.86 (0.50-1.49)
LFIB	LFO	**0.81 (0.67-0.98)**	0.87 (0.67-1.18)	0.87 (0.67-1.18)	1.01 (0.85-1.20)	1.06 (0.77-1.49)	1.06 (0.77-1.49)
LFIB	LFOB	0.99 (0.85-1.14)	1.02 (0.83-1.30)	1.03 (0.84-1.30)	1.05 (0.94-1.17)	1.09 (0.86-1.38)	1.09 (0.86-1.38)
LFIB	LFOC	**0.69 (0.54-0.87)**	0.72 (0.52-1.02)	0.72 (0.52-1.02)	0.87 (0.72-1.05)	0.93 (0.66-1.32)	0.93 (0.66-1.32)
LFIB	LFOP	**0.79 (0.64-0.97)**	0.84 (0.62-1.15)	0.84 (0.62-1.15)	0.98 (0.82-1.19)	1.05 (0.73-1.50)	1.05 (0.73-1.50)
LFIB	LFOI	1.16 (0.89-1.51)	1.23 (0.85-1.84)	1.23 (0.85-1.83)	**1.79 (1.41-2.29)**	**1.90 (1.20-3.05)**	**1.90 (1.20-3.04)**
LFIB	LFOIB	1.16 (0.95-1.42)	1.08 (0.73-1.46)	1.08 (0.74-1.46)	1.09 (0.95-1.25)	0.98 (0.72-1.32)	0.98 (0.71-1.32)
LFIB	LFOIP	-	-	-	**2.00 (1.19-3.40)**	2.13 (0.97-4.70)	2.13 (0.98-4.65)
LFIB	LF	0.98 (0.72-1.33)	1.04 (0.67-1.69)	1.04 (0.67-1.69)	1.22 (0.92-1.63)	1.31 (0.73-2.33)	1.31 (0.74-2.34)
LFIB	LFC	0.93 (0.63-1.37)	0.98 (0.55-1.83)	0.98 (0.56-1.82)	1.38 (0.96-1.97)	1.47 (0.69-3.10)	1.47 (0.70-3.12)
LFIB	LFP	0.56 (0.27-1.18)	0.59 (0.26-1.35)	0.59 (0.26-1.36)	1.06 (0.66-1.70)	1.13 (0.56-2.29)	1.12 (0.56-2.28)
LFIB	IO	0.92 (0.74-1.13)	0.98 (0.71-1.40)	0.98 (0.71-1.40)	**1.38 (1.14-1.67)**	1.46 (0.97-2.23)	1.46 (0.96-2.23)
LFIB	IOB	1.28 (0.86-1.91)	1.33 (0.80-2.28)	1.33 (0.80-2.26)	1.15 (0.80-1.66)	1.20 (0.66-2.17)	1.20 (0.66-2.18)
LFIB	XB	**0.44 (0.24-0.82)**	0.47 (0.21-1.04)	0.47 (0.21-1.05)	0.76 (0.39-1.47)	0.80 (0.30-2.10)	0.79 (0.30-2.08)
LFIB	XIB	0.94 (0.83-1.06)	0.95 (0.76-1.19)	0.95 (0.76-1.19)	1.03 (0.95-1.12)	1.06 (0.80-1.41)	1.06 (0.80-1.41)
LFIB	XO	0.83 (0.67-1.03)	0.88 (0.64-1.28)	0.89 (0.64-1.27)	1.07 (0.88-1.30)	1.13 (0.75-1.69)	1.13 (0.76-1.69)
LFIB	XOB	0.91 (0.77-1.08)	0.91 (0.70-1.19)	0.91 (0.70-1.18)	1.00 (0.89-1.13)	1.05 (0.77-1.42)	1.05 (0.77-1.42)
LFIB	XOBC	0.79 (0.61-1.03)	0.79 (0.51-1.23)	0.79 (0.51-1.22)	0.82 (0.68-1.00)	0.86 (0.50-1.48)	0.86 (0.50-1.48)

T1	T2	Overall survival			Progression-free survival		
		Fixed effects	Random effects	Meta-regression	Fixed effects	Random effects	Meta-regression
LFIB	X	**0.56 (0.33-0.96)**	0.59 (0.31-1.16)	0.60 (0.31-1.16)	1.43 (0.77-2.64)	1.50 (0.66-3.43)	1.50 (0.66-3.41)
LFIC	LFIP	1.15 (0.98-1.35)	1.23 (0.93-1.69)	1.23 (0.93-1.69)	1.12 (0.95-1.33)	1.18 (0.81-1.72)	1.17 (0.80-1.72)
LFIC	LFIBP	0.85 (0.63-1.15)	0.89 (0.59-1.38)	0.90 (0.59-1.39)	0.74 (0.52-1.07)	0.81 (0.47-1.41)	0.81 (0.47-1.40)
LFIC	LFO	0.98 (0.84-1.14)	1.07 (0.84-1.44)	1.07 (0.84-1.44)	0.92 (0.79-1.08)	1.00 (0.72-1.40)	1.00 (0.72-1.39)
LFIC	LFOB	1.19 (0.97-1.47)	1.26 (0.93-1.79)	1.26 (0.93-1.79)	0.95 (0.80-1.13)	1.02 (0.71-1.47)	1.02 (0.71-1.47)
LFIC	LFOC	0.83 (0.69-1.01)	0.89 (0.69-1.18)	0.89 (0.68-1.18)	**0.79 (0.66-0.94)**	0.87 (0.64-1.20)	0.87 (0.64-1.20)
LFIC	LFOP	0.95 (0.79-1.15)	1.03 (0.76-1.45)	1.03 (0.77-1.45)	0.89 (0.75-1.07)	0.98 (0.67-1.45)	0.98 (0.67-1.45)
LFIC	LFOI	**1.41 (1.12-1.77)**	**1.52 (1.07-2.26)**	**1.51 (1.07-2.25)**	**1.63 (1.30-2.05)**	**1.78 (1.12-2.88)**	**1.78 (1.12-2.86)**
LFIC	LFOIB	**1.41 (1.09-1.82)**	1.33 (0.83-1.97)	1.33 (0.83-1.97)	0.99 (0.82-1.21)	0.92 (0.60-1.40)	0.92 (0.60-1.39)
LFIC	LFOIP	-	-	-	**1.82 (1.08-3.06)**	1.99 (0.91-4.42)	1.99 (0.91-4.36)
LFIC	LF	1.19 (0.90-1.57)	1.28 (0.84-2.08)	1.28 (0.84-2.07)	1.11 (0.85-1.46)	1.22 (0.69-2.19)	1.22 (0.69-2.20)
LFIC	LFC	1.12 (0.77-1.62)	1.21 (0.69-2.25)	1.21 (0.69-2.24)	1.25 (0.88-1.77)	1.37 (0.65-2.92)	1.37 (0.66-2.93)
LFIC	LFP	0.67 (0.32-1.42)	0.73 (0.32-1.67)	0.73 (0.32-1.68)	0.96 (0.60-1.54)	1.05 (0.52-2.18)	1.05 (0.51-2.17)
LFIC	IO	1.11 (0.94-1.31)	1.20 (0.89-1.72)	1.20 (0.89-1.72)	**1.25 (1.05-1.49)**	1.37 (0.90-2.11)	1.37 (0.90-2.10)
LFIC	IOB	**1.55 (1.01-2.38)**	1.64 (0.94-2.97)	1.64 (0.94-2.96)	1.05 (0.71-1.54)	1.12 (0.59-2.18)	1.12 (0.58-2.16)
LFIC	XB	0.53 (0.29-0.98)	0.58 (0.26-1.28)	0.58 (0.27-1.29)	0.69 (0.35-1.33)	0.75 (0.29-1.96)	0.74 (0.28-1.96)
LFIC	XIB	1.14 (0.93-1.39)	1.17 (0.83-1.67)	1.16 (0.83-1.66)	0.94 (0.80-1.11)	0.99 (0.65-1.51)	0.99 (0.66-1.51)
LFIC	XO	1.01 (0.84-1.20)	1.09 (0.80-1.56)	1.09 (0.81-1.56)	0.97 (0.81-1.17)	1.05 (0.71-1.60)	1.05 (0.71-1.59)
LFIC	XOB	1.10 (0.88-1.38)	1.12 (0.79-1.62)	1.12 (0.79-1.62)	0.91 (0.76-1.09)	0.98 (0.65-1.50)	0.98 (0.65-1.50)
LFIC	XOBC	0.96 (0.71-1.29)	0.98 (0.60-1.63)	0.97 (0.60-1.62)	**0.75 (0.59-0.94)**	0.80 (0.44-1.49)	0.80 (0.44-1.49)
LFIC	X	0.67 (0.40-1.14)	0.73 (0.38-1.43)	0.73 (0.38-1.43)	1.30 (0.71-2.38)	1.41 (0.62-3.23)	1.40 (0.62-3.21)
LFIP	LFIBP	0.74 (0.55-1.01)	0.73 (0.48-1.10)	0.73 (0.48-1.10)	**0.66 (0.46-0.95)**	0.69 (0.41-1.17)	0.69 (0.41-1.17)
LFIP	LFO	0.86 (0.73-1.01)	0.87 (0.68-1.15)	0.87 (0.68-1.15)	**0.82 (0.70-0.97)**	0.85 (0.60-1.20)	0.85 (0.61-1.20)
LFIP	LFOB	1.04 (0.83-1.30)	1.03 (0.74-1.43)	1.03 (0.74-1.43)	0.85 (0.70-1.03)	0.86 (0.59-1.27)	0.87 (0.60-1.27)
LFIP	LFOC	**0.72 (0.58-0.90)**	0.72 (0.52-1.01)	0.72 (0.52-1.01)	**0.70 (0.58-0.86)**	0.74 (0.51-1.09)	0.74 (0.51-1.09)
LFIP	LFOP	0.83 (0.68-1.01)	0.84 (0.62-1.13)	0.84 (0.62-1.13)	**0.80 (0.66-0.96)**	0.83 (0.58-1.20)	0.83 (0.58-1.20)
LFIP	LFOI	1.23 (0.97-1.55)	1.23 (0.87-1.77)	1.23 (0.87-1.76)	**1.45 (1.16-1.81)**	1.51 (0.97-2.38)	1.51 (0.97-2.38)
LFIP	LFOIB	1.23 (0.93-1.61)	1.08 (0.66-1.61)	1.08 (0.66-1.61)	0.88 (0.71-1.10)	0.78 (0.50-1.20)	0.78 (0.50-1.20)
LFIP	LFOIP	-	-	-	1.63 (0.97-2.72)	1.70 (0.78-3.69)	1.70 (0.78-3.66)
LFIP	LF	1.03 (0.78-1.37)	1.04 (0.68-1.63)	1.05 (0.68-1.62)	0.99 (0.76-1.30)	1.04 (0.59-1.83)	1.04 (0.60-1.84)
LFIP	LFC	0.98 (0.67-1.41)	0.98 (0.56-1.78)	0.99 (0.56-1.76)	1.12 (0.79-1.58)	1.16 (0.56-2.44)	1.17 (0.57-2.46)
LFIP	LFP	0.59 (0.28-1.23)	0.59 (0.26-1.34)	0.59 (0.26-1.34)	0.86 (0.53-1.38)	0.89 (0.44-1.82)	0.89 (0.44-1.82)
LFIP	IO	0.97 (0.81-1.15)	0.98 (0.72-1.35)	0.98 (0.72-1.35)	1.12 (0.94-1.33)	1.16 (0.78-1.76)	1.17 (0.78-1.76)
LFIP	IOB	1.35 (0.87-2.09)	1.34 (0.75-2.38)	1.33 (0.75-2.37)	0.93 (0.63-1.39)	0.95 (0.49-1.86)	0.95 (0.49-1.86)
LFIP	XB	**0.46 (0.25-0.86)**	0.47 (0.21-1.02)	0.47 (0.21-1.04)	0.61 (0.32-1.19)	0.63 (0.24-1.68)	0.63 (0.24-1.66)
LFIP	XIB	0.99 (0.79-1.24)	0.95 (0.66-1.34)	0.95 (0.66-1.34)	0.84 (0.69-1.02)	0.84 (0.55-1.30)	0.84 (0.55-1.30)
LFIP	XO	0.88 (0.73-1.06)	0.89 (0.64-1.24)	0.89 (0.65-1.24)	0.87 (0.72-1.05)	0.90 (0.60-1.36)	0.90 (0.60-1.36)
LFIP	XOB	0.96 (0.75-1.23)	0.91 (0.63-1.30)	0.91 (0.63-1.30)	0.81 (0.66-1.00)	0.83 (0.54-1.29)	0.83 (0.54-1.29)
LFIP	XOBC	0.83 (0.61-1.14)	0.80 (0.47-1.30)	0.79 (0.47-1.30)	**0.67 (0.52-0.86)**	0.68 (0.37-1.28)	0.68 (0.37-1.28)
LFIP	X	0.59 (0.35-1.00)	0.59 (0.31-1.15)	0.60 (0.31-1.15)	1.16 (0.63-2.14)	1.19 (0.52-2.75)	1.19 (0.52-2.74)
LFIBP	LFO	1.15 (0.85-1.56)	1.20 (0.81-1.82)	1.20 (0.81-1.82)	1.24 (0.86-1.77)	1.23 (0.73-2.08)	1.23 (0.74-2.09)
LFIBP	LFOB	1.40 (0.99-1.99)	1.42 (0.89-2.27)	1.41 (0.89-2.28)	1.28 (0.87-1.86)	1.26 (0.72-2.22)	1.26 (0.72-2.23)
LFIBP	LFOC	0.98 (0.70-1.37)	0.99 (0.63-1.58)	0.99 (0.63-1.57)	1.06 (0.73-1.54)	1.08 (0.62-1.88)	1.08 (0.62-1.88)
LFIBP	LFOP	1.12 (0.81-1.55)	1.15 (0.74-1.81)	1.15 (0.75-1.81)	1.20 (0.83-1.74)	1.21 (0.69-2.13)	1.21 (0.69-2.13)
LFIBP	LFOI	**1.65 (1.17-2.33)**	**1.70 (1.09-2.72)**	**1.69 (1.08-2.70)**	**2.19 (1.49-3.21)**	**2.20 (1.24-3.92)**	**2.20 (1.24-3.92)**

T1	T2	Overall survival			Progression-free survival		
		Fixed effects	Random effects	Meta-regression	Fixed effects	Random effects	Meta-regression
LFIBP	LFOIB	**1.65 (1.13-2.42)**	1.48 (0.82-2.51)	1.48 (0.82-2.49)	1.33 (0.90-1.97)	1.14 (0.62-2.08)	1.14 (0.62-2.08)
LFIBP	LFOIP	-	-	-	**2.45 (1.34-4.48)**	**2.47 (1.06-5.83)**	**2.47 (1.06-5.76)**
LFIBP	LF	1.39 (0.96-2.03)	1.44 (0.87-2.45)	1.43 (0.86-2.46)	1.49 (0.99-2.26)	1.51 (0.78-2.94)	1.52 (0.78-2.98)
LFIBP	LFC	1.31 (0.84-2.06)	1.36 (0.72-2.63)	1.35 (0.72-2.61)	**1.68 (1.05-2.68)**	1.70 (0.75-3.86)	1.70 (0.75-3.88)
LFIBP	LFP	0.79 (0.36-1.74)	0.82 (0.34-1.98)	0.81 (0.33-1.97)	1.29 (0.73-2.29)	1.30 (0.57-3.00)	1.31 (0.57-3.00)
LFIBP	IO	1.30 (0.96-1.77)	1.35 (0.89-2.08)	1.34 (0.89-2.09)	1.68 (1.18-2.41)	1.70 (0.98-2.95)	1.70 (0.98-2.96)
LFIBP	IOB	**1.82 (1.09-3.03)**	1.84 (0.95-3.61)	1.83 (0.95-3.59)	1.41 (0.84-2.35)	1.39 (0.63-3.05)	1.39 (0.64-3.05)
LFIBP	XB	0.63 (0.32-1.22)	0.64 (0.28-1.51)	0.65 (0.28-1.50)	0.93 (0.44-1.94)	0.92 (0.33-2.62)	0.92 (0.32-2.63)
LFIBP	XIB	1.33 (0.94-1.89)	1.31 (0.80-2.13)	1.30 (0.80-2.12)	1.26 (0.86-1.84)	1.23 (0.67-2.24)	1.23 (0.67-2.26)
LFIBP	XO	1.18 (0.86-1.62)	1.22 (0.79-1.93)	1.22 (0.79-1.93)	1.31 (0.90-1.89)	1.31 (0.74-2.32)	1.31 (0.74-2.32)
LFIBP	XOB	1.29 (0.90-1.86)	1.26 (0.76-2.05)	1.25 (0.76-2.05)	1.22 (0.83-1.80)	1.21 (0.66-2.22)	1.21 (0.66-2.23)
LFIBP	XOBC	1.12 (0.75-1.69)	1.10 (0.59-1.99)	1.09 (0.59-1.99)	1.00 (0.67-1.51)	0.99 (0.47-2.11)	1.00 (0.47-2.11)
LFIBP	X	0.79 (0.44-1.42)	0.82 (0.40-1.70)	0.82 (0.40-1.69)	1.75 (0.87-3.47)	1.74 (0.70-4.35)	1.73 (0.69-4.38)
LFO	LFOB	1.21 (0.98-1.51)	1.18 (0.85-1.61)	1.18 (0.85-1.60)	1.03 (0.86-1.24)	1.02 (0.72-1.44)	1.02 (0.73-1.44)
LFO	LFOC	0.85 (0.71-1.01)	0.83 (0.63-1.07)	0.83 (0.63-1.07)	**0.86 (0.75-0.98)**	0.87 (0.68-1.13)	0.87 (0.68-1.13)
LFO	LFOP	0.97 (0.85-1.11)	0.96 (0.77-1.19)	0.96 (0.77-1.19)	0.97 (0.86-1.10)	0.98 (0.74-1.30)	0.98 (0.74-1.30)
LFO	LFOI	**1.43 (1.14-1.81)**	1.42 (1.00-1.98)	**1.41 (1.01-1.98)**	**1.77 (1.41-2.22)**	**1.78 (1.15-2.78)**	**1.78 (1.15-2.77)**
LFO	LFOIB	**1.43 (1.09-1.89)**	1.24 (0.74-1.85)	1.24 (0.74-1.85)	1.08 (0.87-1.33)	0.92 (0.60-1.38)	0.92 (0.60-1.38)
LFO	LFOIP	-	-	-	**1.98 (1.19-3.33)**	2.00 (0.92-4.34)	2.00 (0.93-4.30)
LFO	LF	1.21 (0.92-1.60)	1.20 (0.78-1.83)	1.20 (0.78-1.83)	1.21 (0.92-1.59)	1.23 (0.70-2.13)	1.23 (0.71-2.14)
LFO	LFC	1.14 (0.79-1.65)	1.13 (0.64-2.00)	1.13 (0.64-1.99)	1.36 (0.96-1.92)	1.38 (0.66-2.85)	1.38 (0.67-2.86)
LFO	LFP	0.69 (0.33-1.42)	0.68 (0.31-1.49)	0.68 (0.30-1.49)	1.04 (0.67-1.64)	1.06 (0.55-2.06)	1.06 (0.54-2.06)
LFO	IO	1.13 (0.97-1.32)	1.12 (0.86-1.46)	1.12 (0.86-1.46)	**1.36 (1.17-1.58)**	1.38 (0.96-1.96)	1.37 (0.96-1.96)
LFO	IOB	**1.58 (1.02-2.43)**	1.53 (0.86-2.70)	1.53 (0.86-2.67)	1.14 (0.77-1.68)	1.13 (0.59-2.15)	1.12 (0.59-2.13)
LFO	XB	**0.54 (0.30-0.99)**	0.54 (0.26-1.12)	0.54 (0.26-1.13)	0.75 (0.39-1.42)	0.75 (0.30-1.85)	0.74 (0.30-1.84)
LFO	XIB	1.16 (0.93-1.45)	1.09 (0.75-1.53)	1.09 (0.75-1.52)	1.02 (0.85-1.23)	0.99 (0.65-1.51)	0.99 (0.65-1.50)
LFO	XO	1.02 (0.93-1.12)	1.02 (0.83-1.24)	1.02 (0.83-1.24)	1.06 (0.97-1.16)	1.06 (0.84-1.33)	1.06 (0.84-1.34)
LFO	XOB	1.12 (0.88-1.43)	1.05 (0.72-1.48)	1.04 (0.72-1.47)	0.99 (0.82-1.20)	0.98 (0.65-1.48)	0.98 (0.65-1.47)
LFO	XOBC	0.98 (0.72-1.33)	0.91 (0.54-1.47)	0.91 (0.54-1.47)	0.81 (0.64-1.04)	0.80 (0.44-1.48)	0.81 (0.44-1.47)
LFO	X	0.69 (0.42-1.14)	0.68 (0.37-1.24)	0.68 (0.37-1.24)	1.41 (0.78-2.54)	1.41 (0.66-3.00)	1.40 (0.66-2.99)
LFOB	LFOC	**0.70 (0.54-0.90)**	0.70 (0.48-1.02)	0.70 (0.48-1.02)	0.83 (0.68-1.01)	0.86 (0.60-1.22)	0.86 (0.60-1.22)
LFOB	LFOP	0.80 (0.64-1.00)	0.82 (0.59-1.12)	0.82 (0.60-1.12)	0.94 (0.78-1.14)	0.96 (0.68-1.37)	0.96 (0.68-1.37)
LFOB	LFOI	1.18 (0.89-1.58)	1.20 (0.79-1.84)	1.20 (0.79-1.83)	**1.71 (1.33-2.21)**	**1.75 (1.07-2.86)**	**1.75 (1.07-2.85)**
LFOB	LFOIB	1.18 (0.93-1.50)	1.05 (0.67-1.50)	1.05 (0.67-1.49)	1.04 (0.91-1.20)	0.90 (0.68-1.18)	0.91 (0.68-1.18)
LFOB	LFOIP	-	-	-	**1.92 (1.13-3.26)**	1.96 (0.88-4.38)	1.96 (0.88-4.33)
LFOB	LF	0.99 (0.72-1.38)	1.02 (0.63-1.67)	1.02 (0.63-1.67)	1.17 (0.87-1.57)	1.20 (0.66-2.18)	1.20 (0.66-2.19)
LFOB	LFC	0.94 (0.62-1.41)	0.96 (0.52-1.80)	0.96 (0.52-1.79)	1.31 (0.91-1.90)	1.35 (0.63-2.89)	1.35 (0.64-2.90)
LFOB	LFP	0.57 (0.27-1.20)	0.58 (0.25-1.31)	0.58 (0.25-1.32)	1.01 (0.63-1.62)	1.04 (0.51-2.08)	1.03 (0.51-2.09)
LFOB	IO	0.93 (0.73-1.18)	0.95 (0.66-1.39)	0.95 (0.66-1.40)	**1.32 (1.07-1.62)**	1.35 (0.87-2.09)	1.35 (0.87-2.09)
LFOB	IOB	1.30 (0.89-1.89)	1.30 (0.81-2.08)	1.30 (0.81-2.07)	1.10 (0.78-1.56)	1.10 (0.64-1.90)	1.10 (0.64-1.91)
LFOB	XB	**0.45 (0.24-0.84)**	0.46 (0.20-1.02)	0.46 (0.20-1.02)	0.72 (0.37-1.41)	0.73 (0.28-1.93)	0.73 (0.28-1.92)
LFOB	XIB	0.95 (0.81-1.13)	0.92 (0.70-1.19)	0.92 (0.70-1.19)	0.99 (0.88-1.11)	0.97 (0.71-1.33)	0.97 (0.71-1.33)
LFOB	XO	0.84 (0.67-1.07)	0.86 (0.60-1.26)	0.86 (0.60-1.26)	1.02 (0.83-1.25)	1.04 (0.69-1.57)	1.04 (0.69-1.57)
LFOB	XOB	0.92 (0.82-1.05)	0.89 (0.71-1.08)	0.89 (0.71-1.08)	0.96 (0.87-1.06)	0.96 (0.74-1.24)	0.96 (0.74-1.24)
LFOB	XOBC	0.80 (0.64-1.01)	0.77 (0.51-1.14)	0.77 (0.51-1.14)	0.79 (0.66-0.94)	0.79 (0.47-1.32)	0.79 (0.47-1.32)

T1	T2	Overall survival			Progression-free survival		
		Fixed effects	Random effects	Meta-regression	Fixed effects	Random effects	Meta-regression
LFOB	X	**0.56 (0.33-0.98)**	0.58 (0.29-1.14)	0.58 (0.29-1.14)	1.37 (0.74-2.53)	1.38 (0.60-3.18)	1.37 (0.61-3.15)
LFOC	LFOP	1.15 (0.93-1.42)	1.16 (0.84-1.62)	1.16 (0.84-1.61)	1.13 (0.95-1.35)	1.12 (0.79-1.59)	1.12 (0.79-1.59)
LFOC	LFOI	**1.69 (1.29-2.23)**	**1.71 (1.15-2.57)**	**1.71 (1.15-2.56)**	**2.06 (1.61-2.65)**	**2.04 (1.27-3.28)**	**2.04 (1.27-3.28)**
LFOC	LFOIB	**1.69 (1.24-2.30)**	1.49 (0.88-2.31)	1.49 (0.88-2.31)	1.26 (1.00-1.58)	1.06 (0.68-1.60)	1.06 (0.68-1.59)
LFOC	LFOIP	-	-	-	**2.31 (1.36-3.91)**	**2.29 (1.04-5.08)**	**2.28 (1.04-5.02)**
LFOC	LF	**1.43 (1.04-1.95)**	1.45 (0.91-2.35)	1.45 (0.91-2.34)	**1.41 (1.05-1.89)**	1.40 (0.78-2.51)	1.41 (0.79-2.52)
LFOC	LFC	1.35 (0.90-2.01)	1.37 (0.75-2.54)	1.36 (0.75-2.52)	**1.58 (1.10-2.28)**	1.57 (0.74-3.34)	1.58 (0.75-3.34)
LFOC	LFP	0.81 (0.39-1.71)	0.82 (0.36-1.87)	0.82 (0.35-1.89)	1.22 (0.76-1.95)	1.21 (0.60-2.43)	1.21 (0.60-2.43)
LFOC	IO	**1.34 (1.08-1.66)**	1.35 (0.96-1.94)	1.35 (0.96-1.94)	**1.59 (1.31-1.92)**	**1.57 (1.05-2.36)**	**1.57 (1.04-2.37)**
LFOC	IOB	**1.86 (1.18-2.94)**	**1.85 (1.02-3.38)**	**1.84 (1.02-3.36)**	1.33 (0.89-1.98)	1.29 (0.67-2.46)	1.28 (0.67-2.45)
LFOC	XB	0.64 (0.34-1.19)	0.65 (0.30-1.41)	0.66 (0.30-1.43)	0.87 (0.45-1.68)	0.86 (0.34-2.19)	0.85 (0.33-2.19)
LFOC	XIB	**1.37 (1.05-1.77)**	1.32 (0.87-1.94)	1.31 (0.88-1.94)	1.19 (0.97-1.46)	1.14 (0.74-1.74)	1.14 (0.74-1.74)
LFOC	XO	1.21 (0.99-1.47)	1.23 (0.89-1.72)	1.23 (0.90-1.72)	**1.23 (1.05-1.45)**	1.21 (0.86-1.71)	1.21 (0.86-1.71)
LFOC	XOB	1.33 (1.00-1.75)	1.26 (0.83-1.88)	1.26 (0.83-1.88)	1.16 (0.93-1.43)	1.12 (0.74-1.70)	1.12 (0.74-1.70)
LFOC	XOBC	1.15 (0.82-1.62)	1.10 (0.64-1.86)	1.09 (0.64-1.86)	0.95 (0.73-1.23)	0.92 (0.50-1.70)	0.92 (0.50-1.70)
LFOC	X	0.81 (0.48-1.38)	0.82 (0.43-1.59)	0.83 (0.43-1.60)	1.65 (0.90-3.01)	1.61 (0.73-3.59)	1.60 (0.73-3.57)
LFOP	LFOI	**1.48 (1.14-1.92)**	1.47 (1.00-2.17)	1.47 (1.00-2.16)	**1.82 (1.42-2.33)**	**1.82 (1.12-2.96)**	**1.82 (1.11-2.97)**
LFOP	LFOIB	**1.48 (1.11-1.97)**	1.29 (0.77-1.95)	1.29 (0.77-1.95)	1.11 (0.89-1.39)	0.94 (0.60-1.43)	0.94 (0.61-1.42)
LFOP	LFOIP	-	-	-	**2.04 (1.21-3.45)**	2.04 (0.91-4.55)	2.04 (0.92-4.51)
LFOP	LF	1.25 (0.92-1.68)	1.25 (0.79-1.98)	1.25 (0.79-1.99)	1.24 (0.93-1.66)	1.25 (0.69-2.25)	1.25 (0.69-2.26)
LFOP	LFC	1.18 (0.80-1.73)	1.18 (0.65-2.15)	1.18 (0.65-2.14)	1.40 (0.97-2.01)	1.40 (0.65-3.00)	1.41 (0.66-3.00)
LFOP	LFP	0.71 (0.35-1.45)	0.71 (0.33-1.50)	0.71 (0.32-1.51)	1.07 (0.70-1.66)	1.08 (0.59-1.97)	1.08 (0.59-1.97)
LFOP	IO	1.17 (0.96-1.42)	1.17 (0.84-1.63)	1.17 (0.84-1.62)	1.40 (1.16-1.68)	1.40 (0.92-2.14)	1.40 (0.92-2.13)
LFOP	IOB	**1.63 (1.05-2.52)**	1.60 (0.90-2.82)	1.59 (0.90-2.80)	1.17 (0.79-1.74)	1.15 (0.60-2.20)	1.14 (0.60-2.20)
LFOP	XB	0.56 (0.30-1.03)	0.56 (0.26-1.21)	0.57 (0.26-1.22)	0.77 (0.40-1.48)	0.76 (0.30-1.96)	0.75 (0.29-1.96)
LFOP	XIB	1.19 (0.94-1.51)	1.13 (0.77-1.62)	1.13 (0.78-1.61)	1.05 (0.86-1.28)	1.01 (0.65-1.56)	1.01 (0.65-1.55)
LFOP	XO	1.06 (0.90-1.24)	1.06 (0.79-1.43)	1.06 (0.79-1.43)	1.09 (0.93-1.27)	1.08 (0.75-1.55)	1.08 (0.75-1.55)
LFOP	XOB	1.16 (0.90-1.48)	1.09 (0.75-1.55)	1.09 (0.75-1.54)	1.02 (0.83-1.25)	1.00 (0.66-1.52)	1.00 (0.66-1.52)
LFOP	XOBC	1.01 (0.73-1.38)	0.95 (0.56-1.55)	0.95 (0.56-1.54)	0.84 (0.65-1.08)	0.82 (0.44-1.51)	0.82 (0.44-1.51)
LFOP	X	0.71 (0.42-1.19)	0.71 (0.37-1.35)	0.71 (0.37-1.34)	1.45 (0.80-2.65)	1.43 (0.65-3.22)	1.43 (0.64-3.20)
LFOI	LFOIB	1.00 (0.72-1.39)	0.87 (0.49-1.40)	0.87 (0.50-1.39)	**0.61 (0.46-0.80)**	**0.52 (0.30-0.88)**	**0.52 (0.30-0.88)**
LFOI	LFOIP	-	-	-	1.12 (0.70-1.78)	1.12 (0.60-2.11)	1.12 (0.60-2.10)
LFOI	LF	0.84 (0.61-1.16)	0.85 (0.53-1.38)	0.85 (0.53-1.37)	**0.68 (0.50-0.93)**	0.69 (0.37-1.25)	0.69 (0.38-1.26)
LFOI	LFC	0.80 (0.53-1.19)	0.80 (0.44-1.48)	0.80 (0.44-1.47)	0.77 (0.53-1.12)	0.77 (0.36-1.65)	0.77 (0.36-1.67)
LFOI	LFP	0.48 (0.22-1.03)	0.48 (0.20-1.13)	0.48 (0.20-1.13)	**0.59 (0.36-0.97)**	0.59 (0.27-1.28)	0.59 (0.27-1.29)
LFOI	IO	0.79 (0.62-1.00)	0.79 (0.55-1.15)	0.79 (0.55-1.15)	**0.77 (0.61-0.96)**	0.77 (0.48-1.23)	0.77 (0.48-1.24)
LFOI	IOB	1.10 (0.69-1.76)	1.08 (0.58-2.03)	1.08 (0.58-2.02)	**0.64 (0.42-0.99)**	0.63 (0.30-1.32)	0.63 (0.30-1.31)
LFOI	XB	**0.38 (0.20-0.72)**	**0.38 (0.17-0.85)**	**0.39 (0.17-0.86)**	**0.42 (0.21-0.83)**	0.42 (0.15-1.15)	0.42 (0.15-1.14)
LFOI	XIB	0.81 (0.60-1.08)	0.77 (0.49-1.18)	0.77 (0.49-1.17)	**0.58 (0.45-0.74)**	**0.56 (0.33-0.95)**	**0.56 (0.33-0.95)**
LFOI	XO	**0.71 (0.56-0.92)**	0.72 (0.49-1.07)	0.72 (0.49-1.07)	**0.60 (0.47-0.76)**	**0.59 (0.36-0.97)**	**0.59 (0.36-0.98)**
LFOI	XOB	0.78 (0.58-1.06)	0.74 (0.46-1.14)	0.74 (0.47-1.14)	**0.56 (0.43-0.73)**	**0.55 (0.32-0.94)**	**0.55 (0.32-0.94)**
LFOI	XOBC	**0.68 (0.47-0.98)**	0.65 (0.36-1.12)	0.64 (0.36-1.12)	**0.46 (0.34-0.62)**	**0.45 (0.22-0.90)**	**0.45 (0.22-0.91)**
LFOI	X	**0.48 (0.28-0.83)**	**0.48 (0.24-0.96)**	**0.48 (0.24-0.96)**	0.80 (0.42-1.50)	0.79 (0.33-1.89)	0.79 (0.33-1.88)
LFOIB	LFOIP	-	-	-	**1.84 (1.07-3.16)**	2.17 (0.95-5.03)	2.17 (0.96-4.98)
LFOIB	LF	0.84 (0.59-1.21)	0.97 (0.57-1.83)	0.97 (0.57-1.83)	1.12 (0.82-1.54)	1.33 (0.71-2.54)	1.33 (0.72-2.56)

T1	T2	Overall survival			Progression-free survival		
		**Fixed effects**	**Random effects**	**Meta-regression**	**Fixed effects**	**Random effects**	**Meta-regression**
LFOIB	LFC	0.80 (0.51-1.24)	0.91 (0.48-1.93)	0.91 (0.48-1.93)	1.26 (0.86-1.85)	1.49 (0.68-3.34)	1.49 (0.69-3.36)
LFOIB	LFP	0.48 (0.22-1.04)	0.55 (0.23-1.38)	0.55 (0.23-1.38)	0.97 (0.60-1.58)	1.15 (0.55-2.43)	1.14 (0.55-2.43)
LFOIB	IO	0.79 (0.59-1.05)	0.91 (0.59-1.57)	0.91 (0.59-1.56)	1.26 (1.00-1.59)	1.49 (0.92-2.47)	1.49 (0.92-2.49)
LFOIB	IOB	1.10 (0.71-1.72)	1.24 (0.70-2.40)	1.24 (0.69-2.39)	1.06 (0.73-1.54)	1.22 (0.67-2.27)	1.22 (0.67-2.28)
LFOIB	XB	0.38 (0.20-0.73)	0.44 (0.19-1.07)	0.44 (0.19-1.08)	0.70 (0.35-1.37)	0.81 (0.30-2.22)	0.80 (0.30-2.20)
LFOIB	XIB	0.81 (0.64-1.02)	0.88 (0.61-1.38)	0.88 (0.61-1.38)	0.95 (0.81-1.11)	1.08 (0.74-1.60)	1.08 (0.74-1.60)
LFOIB	XO	**0.71 (0.54-0.96)**	0.82 (0.53-1.42)	0.82 (0.53-1.42)	0.98 (0.78-1.24)	1.15 (0.72-1.87)	1.15 (0.72-1.87)
LFOIB	XOB	0.78 (0.61-1.01)	0.85 (0.58-1.34)	0.84 (0.58-1.34)	0.92 (0.78-1.08)	1.06 (0.75-1.55)	1.06 (0.75-1.55)
LFOIB	XOBC	**0.68 (0.49-0.94)**	0.73 (0.44-1.33)	0.73 (0.45-1.32)	**0.75 (0.61-0.94)**	0.87 (0.50-1.57)	0.87 (0.50-1.57)
LFOIB	X	**0.48 (0.27-0.85)**	0.55 (0.27-1.21)	0.56 (0.27-1.22)	1.31 (0.70-2.45)	1.53 (0.65-3.66)	1.52 (0.65-3.64)
LFOIP	LF	-	-	-	0.61 (0.35-1.07)	0.61 (0.25-1.46)	0.61 (0.26-1.48)
LFOIP	LFC	-	-	-	0.69 (0.38-1.25)	0.69 (0.25-1.85)	0.69 (0.26-1.87)
LFOIP	LFP	-	-	-	0.53 (0.27-1.04)	0.53 (0.19-1.44)	0.53 (0.19-1.44)
LFOIP	IO	-	-	-	0.69 (0.41-1.15)	0.69 (0.31-1.51)	0.69 (0.31-1.51)
LFOIP	IOB	-	-	-	0.57 (0.31-1.08)	0.56 (0.21-1.48)	0.56 (0.21-1.47)
LFOIP	XB	-	-	-	**0.38 (0.17-0.86)**	0.37 (0.11-1.23)	0.37 (0.11-1.23)
LFOIP	XIB	-	-	-	**0.51 (0.30-0.87)**	0.50 (0.22-1.14)	0.50 (0.22-1.13)
LFOIP	XO	-	-	-	**0.53 (0.32-0.90)**	0.53 (0.23-1.19)	0.53 (0.24-1.18)
LFOIP	XOB	-	-	-	**0.50 (0.29-0.85)**	0.49 (0.21-1.13)	0.49 (0.22-1.12)
LFOIP	XOBC	-	-	-	**0.41 (0.24-0.71)**	0.40 (0.16-1.04)	0.40 (0.16-1.03)
LFOIP	X	-	-	-	0.71 (0.33-1.56)	0.71 (0.24-2.08)	0.70 (0.24-2.08)
LF	LFC	0.94 (0.74-1.20)	0.94 (0.65-1.37)	0.94 (0.65-1.37)	1.12 (0.91-1.39)	1.12 (0.70-1.80)	1.12 (0.70-1.81)
LF	LFP	0.57 (0.26-1.23)	0.57 (0.23-1.37)	0.57 (0.23-1.37)	0.86 (0.51-1.45)	0.86 (0.37-2.02)	0.86 (0.37-2.01)
LF	IO	0.94 (0.71-1.24)	0.94 (0.60-1.46)	0.94 (0.60-1.47)	1.13 (0.86-1.48)	1.12 (0.63-2.01)	1.12 (0.63-1.99)
LF	IOB	1.31 (0.79-2.14)	1.28 (0.64-2.51)	1.27 (0.64-2.49)	0.94 (0.60-1.48)	0.92 (0.41-2.07)	0.91 (0.41-2.05)
LF	XB	**0.45 (0.23-0.87)**	0.45 (0.19-1.05)	0.46 (0.19-1.05)	0.62 (0.31-1.24)	0.61 (0.21-1.76)	0.60 (0.21-1.75)
LF	XIB	0.96 (0.69-1.32)	0.91 (0.54-1.49)	0.91 (0.54-1.48)	0.84 (0.63-1.13)	0.81 (0.43-1.53)	0.81 (0.43-1.52)
LF	XO	0.85 (0.63-1.14)	0.85 (0.53-1.36)	0.85 (0.53-1.35)	0.87 (0.66-1.16)	0.86 (0.47-1.58)	0.86 (0.47-1.57)
LF	XOB	0.93 (0.66-1.30)	0.87 (0.51-1.44)	0.87 (0.51-1.43)	0.82 (0.60-1.11)	0.80 (0.42-1.52)	0.80 (0.42-1.50)
LF	XOBC	0.81 (0.55-1.19)	0.76 (0.40-1.39)	0.76 (0.40-1.39)	0.67 (0.48-0.94)	0.66 (0.30-1.43)	0.65 (0.30-1.42)
LF	X	0.57 (0.32-1.01)	0.57 (0.27-1.19)	0.57 (0.27-1.19)	1.17 (0.61-2.23)	1.15 (0.45-2.93)	1.14 (0.45-2.91)
LFC	LFP	0.60 (0.27-1.36)	0.60 (0.23-1.57)	0.60 (0.23-1.57)	0.77 (0.44-1.35)	0.77 (0.29-2.04)	0.76 (0.29-2.02)
LFC	IO	0.99 (0.68-1.44)	0.99 (0.55-1.79)	0.99 (0.55-1.78)	1.00 (0.71-1.42)	1.00 (0.47-2.13)	0.99 (0.47-2.11)
LFC	IOB	1.39 (0.79-2.40)	1.36 (0.62-2.94)	1.35 (0.62-2.91)	0.84 (0.51-1.39)	0.82 (0.32-2.10)	0.81 (0.32-2.07)
LFC	XB	**0.48 (0.24-0.96)**	0.47 (0.19-1.20)	0.48 (0.19-1.21)	0.55 (0.27-1.14)	0.54 (0.17-1.74)	0.54 (0.17-1.71)
LFC	XIB	1.02 (0.68-1.52)	0.96 (0.50-1.79)	0.96 (0.50-1.77)	0.75 (0.52-1.08)	0.72 (0.33-1.61)	0.72 (0.32-1.58)
LFC	XO	0.90 (0.61-1.32)	0.90 (0.49-1.64)	0.90 (0.49-1.64)	0.78 (0.54-1.11)	0.77 (0.36-1.66)	0.77 (0.36-1.64)
LFC	XOB	0.98 (0.65-1.50)	0.93 (0.48-1.73)	0.92 (0.48-1.72)	0.73 (0.50-1.06)	0.71 (0.32-1.58)	0.71 (0.32-1.56)
LFC	XOBC	0.86 (0.54-1.35)	0.81 (0.38-1.64)	0.80 (0.38-1.63)	0.60 (0.40-0.89)	0.59 (0.23-1.46)	0.58 (0.23-1.44)
LFC	X	0.60 (0.32-1.12)	0.60 (0.26-1.38)	0.61 (0.26-1.37)	1.04 (0.52-2.06)	1.03 (0.36-2.92)	1.02 (0.36-2.90)
LFP	IO	1.64 (0.78-3.44)	1.65 (0.72-3.78)	1.66 (0.72-3.86)	1.30 (0.81-2.09)	1.30 (0.62-2.72)	1.30 (0.62-2.72)
LFP	IOB	2.30 (0.99-5.30)	2.26 (0.87-5.81)	2.25 (0.87-5.90)	1.09 (0.61-1.96)	1.06 (0.44-2.60)	1.06 (0.44-2.59)
LFP	XB	0.79 (0.31-2.02)	0.79 (0.27-2.37)	0.80 (0.27-2.38)	0.72 (0.33-1.57)	0.71 (0.23-2.17)	0.70 (0.23-2.16)
LFP	XIB	1.68 (0.79-3.57)	1.60 (0.68-3.72)	1.60 (0.68-3.75)	0.98 (0.61-1.57)	0.94 (0.45-1.98)	0.94 (0.44-1.98)
LFP	XO	1.49 (0.72-3.10)	1.50 (0.67-3.38)	1.50 (0.67-3.45)	1.01 (0.64-1.61)	1.00 (0.49-2.02)	1.00 (0.49-2.04)

T1	T2	Overall survival			Progression-free survival		
		**Fixed effects**	**Random effects**	**Meta-regression**	**Fixed effects**	**Random effects**	**Meta-regression**
LFP	XOB	1.63 (0.76-3.48)	1.54 (0.66-3.56)	1.54 (0.66-3.61)	0.95 (0.59-1.53)	0.93 (0.44-1.95)	0.93 (0.44-1.94)
LFP	XOBC	1.42 (0.65-3.09)	1.34 (0.53-3.31)	1.34 (0.53-3.36)	0.78 (0.47-1.28)	0.76 (0.32-1.81)	0.76 (0.32-1.80)
LFP	X	1.00 (0.41-2.41)	1.00 (0.37-2.76)	1.01 (0.37-2.78)	1.35 (0.64-2.83)	1.33 (0.49-3.64)	1.33 (0.49-3.64)
IO	IOB	1.40 (0.90-2.18)	1.37 (0.75-2.48)	1.36 (0.75-2.47)	0.84 (0.56-1.25)	0.82 (0.41-1.65)	0.82 (0.41-1.65)
IO	XB	**0.48 (0.26-0.89)**	0.48 (0.22-1.05)	0.49 (0.22-1.05)	0.55 (0.28-1.06)	0.54 (0.21-1.43)	0.54 (0.20-1.43)
IO	XIB	1.02 (0.81-1.30)	0.97 (0.64-1.43)	0.97 (0.64-1.42)	**0.75 (0.61-0.92)**	0.72 (0.44-1.18)	0.72 (0.44-1.18)
IO	XO	0.91 (0.76-1.09)	0.91 (0.65-1.27)	0.91 (0.65-1.26)	**0.78 (0.65-0.93)**	0.77 (0.50-1.18)	0.77 (0.50-1.18)
IO	XOB	0.99 (0.77-1.28)	0.93 (0.61-1.38)	0.93 (0.61-1.38)	**0.73 (0.59-0.90)**	0.71 (0.44-1.16)	0.71 (0.44-1.17)
IO	XOBC	0.86 (0.63-1.19)	0.81 (0.46-1.36)	0.81 (0.47-1.36)	**0.60 (0.46-0.78)**	0.58 (0.30-1.14)	0.59 (0.30-1.14)
IO	X	0.61 (0.36-1.03)	0.61 (0.31-1.17)	0.61 (0.31-1.17)	1.04 (0.57-1.90)	1.03 (0.45-2.37)	1.02 (0.44-2.35)
IOB	XB	**0.34 (0.17-0.72)**	**0.35 (0.14-0.89)**	**0.36 (0.14-0.90)**	0.66 (0.31-1.39)	0.66 (0.22-2.02)	0.66 (0.22-2.02)
IOB	XIB	0.73 (0.49-1.11)	0.71 (0.41-1.21)	0.71 (0.41-1.21)	0.90 (0.62-1.29)	0.88 (0.47-1.66)	0.89 (0.47-1.66)
IOB	XO	0.65 (0.42-1.01)	0.66 (0.37-1.21)	0.67 (0.37-1.21)	0.93 (0.62-1.39)	0.94 (0.47-1.86)	0.94 (0.48-1.87)
IOB	XOB	0.71 (0.48-1.06)	0.68 (0.40-1.13)	0.68 (0.41-1.13)	0.87 (0.61-1.25)	0.87 (0.48-1.60)	0.87 (0.48-1.60)
IOB	XOBC	**0.62 (0.40-0.96)**	0.59 (0.32-1.09)	0.60 (0.32-1.09)	0.71 (0.48-1.06)	0.71 (0.34-1.52)	0.72 (0.34-1.52)
IOB	X	**0.43 (0.23-0.84)**	0.45 (0.19-1.02)	0.45 (0.20-1.02)	1.24 (0.61-2.52)	1.25 (0.46-3.39)	1.25 (0.47-3.38)
XB	XIB	**2.13 (1.13-4.01)**	2.03 (0.89-4.54)	2.00 (0.88-4.55)	1.36 (0.70-2.67)	1.33 (0.49-3.61)	1.34 (0.50-3.59)
XB	XO	**1.89 (1.04-3.40)**	1.90 (0.93-3.86)	1.87 (0.93-3.88)	1.41 (0.75-2.68)	1.42 (0.59-3.38)	1.43 (0.59-3.41)
XB	XOB	**2.07 (1.09-3.91)**	1.95 (0.85-4.37)	1.92 (0.84-4.38)	1.32 (0.68-2.60)	1.31 (0.49-3.54)	1.33 (0.49-3.54)
XB	XOBC	1.80 (0.92-3.51)	1.70 (0.69-4.10)	1.67 (0.69-4.06)	1.08 (0.55-2.16)	1.08 (0.36-3.21)	1.09 (0.36-3.19)
XB	X	1.27 (0.92-1.75)	1.27 (0.82-1.95)	1.26 (0.82-1.95)	**1.89 (1.45-2.45)**	**1.89 (1.15-3.09)**	**1.89 (1.15-3.12)**
XIB	XO	0.89 (0.70-1.13)	0.93 (0.63-1.43)	0.94 (0.64-1.42)	1.04 (0.84-1.28)	1.06 (0.66-1.72)	1.07 (0.66-1.71)
XIB	XOB	0.97 (0.81-1.16)	0.96 (0.74-1.25)	0.96 (0.74-1.24)	0.97 (0.87-1.08)	0.99 (0.74-1.32)	0.99 (0.74-1.32)
XIB	XOBC	0.84 (0.65-1.10)	0.84 (0.54-1.30)	0.84 (0.54-1.29)	**0.80 (0.66-0.96)**	0.81 (0.47-1.38)	0.81 (0.47-1.39)
XIB	X	0.59 (0.35-1.03)	0.63 (0.31-1.27)	0.63 (0.31-1.27)	1.39 (0.75-2.57)	1.42 (0.60-3.37)	1.41 (0.60-3.35)
XO	XOB	1.10 (0.85-1.41)	1.03 (0.67-1.52)	1.03 (0.67-1.52)	0.94 (0.76-1.16)	0.93 (0.58-1.48)	0.93 (0.58-1.48)
XO	XOBC	0.95 (0.69-1.31)	0.90 (0.51-1.51)	0.89 (0.51-1.50)	0.77 (0.59-1.00)	0.76 (0.40-1.46)	0.76 (0.40-1.45)
XO	X	0.67 (0.41-1.10)	0.67 (0.38-1.19)	0.67 (0.38-1.18)	1.34 (0.75-2.38)	1.33 (0.65-2.73)	1.33 (0.65-2.72)
XOB	XOBC	0.87 (0.72-1.06)	0.87 (0.61-1.24)	0.87 (0.61-1.23)	**0.82 (0.71-0.95)**	0.82 (0.52-1.28)	0.82 (0.52-1.29)
XOB	X	0.61 (0.35-1.07)	0.65 (0.33-1.32)	0.66 (0.33-1.32)	1.43 (0.76-2.65)	1.44 (0.61-3.39)	1.43 (0.61-3.38)
XOBC	X	0.70 (0.39-1.27)	0.75 (0.34-1.65)	0.76 (0.35-1.64)	1.74 (0.92-3.28)	1.75 (0.66-4.64)	1.74 (0.67-4.59)
LFI	LFIC	0.94 (0.74-1.20)	0.94 (0.65-1.37)	0.94 (0.65-1.37)	**0.69 (0.59-0.80)**	**0.64 (0.47-0.87)**	**0.64 (0.47-0.87)**

X, capecitabine; B, bevacizumab; C, cetuximab; F, 5-fluorouracil; I, irinotecan; L, leucovorin; O, oxaliplatin; P, panitumumab. Bold fonts indicate significant difference.

Supplementary Table S6. Odds ratios and 95% credible intervals for pairwise estimates of adverse events grade ≥3 and serious adverse events.

T1	T2	Adverse events grade ≥3			Serious adverse events		
		**Fixed effects**	**Random effects**	**Meta-regression**	**Fixed effects**	**Random effects**	**Meta-regression**
LFI	LFIB	**0.77 (0.61-0.98)**	0.68 (0.28-1.69)	0.71 (0.22-2.30)	0.80 (0.62-1.02)	0.79 (0.50-1.23)	0.72 (0.37-1.23)
LFI	LFIC	**1.79 (1.45-2.22)**	1.50 (0.50-4.48)	1.52 (0.46-5.03)	1.05 (0.46-2.45)	1.03 (0.34-2.91)	0.88 (0.23-2.86)
LFI	LFIP	**2.12 (1.68-2.67)**	2.04 (0.78-5.35)	2.18 (0.50-9.40)	**1.50 (1.20-1.87)**	1.46 (0.88-2.14)	1.06 (0.42-2.33)
LFI	LFIBP	0.80 (0.52-1.22)	2.14 (0.73-7.20)	2.34 (0.41-15.3)	-	-	-
LFI	LFO	1.13 (0.87-1.47)	1.18 (0.52-2.69)	1.19 (0.47-3.03)	**0.54 (0.33-0.88)**	**0.53 (0.27-0.97)**	**0.45 (0.19-0.92)**
LFI	LFOB	1.69 (0.42-6.44)	1.60 (0.09-28.1)	1.81 (0.08-39.2)	0.67 (0.44-1.01)	0.67 (0.35-1.25)	0.60 (0.26-1.26)
LFI	LFOC	**1.87 (1.26-2.77)**	1.72 (0.47-6.22)	1.74 (0.43-6.87)	0.96 (0.47-2.00)	0.93 (0.35-2.33)	0.80 (0.24-2.25)
LFI	LFOP	**3.45 (1.09-11.4)**	3.33 (0.36-30.7)	3.66 (0.29-44.9)	0.88 (0.55-1.40)	0.89 (0.48-1.65)	0.76 (0.32-1.65)
LFI	LFOIB	3.51 (0.83-14.3)	3.99 (0.17-96.5)	4.55 (0.15-137)	0.58 (0.32-1.07)	0.58 (0.25-1.35)	0.52 (0.18-1.34)
LFI	LF	**0.47 (0.34-0.66)**	0.45 (0.16-1.29)	0.46 (0.15-1.38)	-	-	-
LFI	LFB	1.44 (0.32-6.24)	1.40 (0.05-36.7)	1.58 (0.05-51.3)	-	-	-
LFI	LFBC	-	-	-	0.89 (0.45-1.74)	0.89 (0.34-2.29)	0.79 (0.25-2.33)
LFI	LFP	2.18 (0.50-9.82)	2.08 (0.12-37.5)	2.29 (0.10-53.1)	0.79 (0.31-2.00)	0.78 (0.26-2.38)	0.68 (0.19-2.40)
LFI	XB	-	-	-	0.55 (0.23-1.33)	0.54 (0.17-1.75)	0.50 (0.13-1.80)
LFI	XI	3.80 (0.84-21.6)	3.45 (0.19-68.6)	3.53 (0.17-81.9)	-	-	-
LFI	XIB	1.05 (0.63-1.75)	0.93 (0.14-6.36)	0.96 (0.12-7.76)	1.28 (0.64-2.60)	1.27 (0.50-3.16)	1.16 (0.39-3.23)
LFI	XO	**0.65 (0.47-0.90)**	0.73 (0.21-2.56)	0.74 (0.20-2.81)	-	-	-
LFI	XOB	1.39 (0.34-5.47)	1.41 (0.07-28.7)	1.59 (0.06-40.2)	**0.29 (0.09-0.88)**	0.29 (0.07-1.08)	0.26 (0.06-1.07)
LFI	XOBC	2.30 (0.55-9.50)	2.34 (0.07-71.8)	2.61 (0.07-101)	0.47 (0.14-1.50)	0.46 (0.11-1.99)	0.42 (0.08-1.98)
LFI	X	-	-	-	0.58 (0.21-1.61)	0.57 (0.14-2.24)	0.52 (0.11-2.37)
LFI	O	**0.47 (0.29-0.76)**	0.47 (0.09-2.52)	0.47 (0.08-2.77)	-	-	-
LFIB	LFIC	**2.32 (1.80-3.00)**	2.20 (0.68-7.08)	2.15 (0.61-7.64)	1.32 (0.58-3.07)	1.31 (0.42-3.81)	1.22 (0.33-4.06)
LFIB	LFIP	**2.73 (2.02-3.70)**	**2.99 (1.11-8.11)**	**3.07 (1.05-9.13)**	1.88 (1.39-2.55)	1.83 (1.07-2.89)	1.47 (0.72-2.87)
LFIB	LFIBP	1.03 (0.63-1.67)	3.13 (0.78-14.5)	3.30 (0.69-18.1)	-	-	-
LFIB	LFO	**1.47 (1.04-2.06)**	1.71 (0.53-5.64)	1.69 (0.47-6.12)	0.68 (0.42-1.11)	0.67 (0.33-1.29)	0.63 (0.28-1.30)
LFIB	LFOB	2.19 (0.54-8.42)	2.35 (0.14-41.8)	2.56 (0.13-47.3)	0.84 (0.58-1.21)	0.85 (0.49-1.45)	0.83 (0.45-1.54)
LFIB	LFOC	**2.41 (1.56-3.74)**	2.51 (0.60-10.4)	2.47 (0.54-11.4)	1.20 (0.59-2.51)	1.18 (0.43-3.05)	1.11 (0.36-3.21)
LFIB	LFOP	**4.47 (1.38-14.9)**	4.87 (0.53-45.8)	5.16 (0.51-52.8)	1.10 (0.69-1.75)	1.12 (0.60-2.10)	1.05 (0.53-2.17)
LFIB	LFOIB	**4.55 (1.06-18.8)**	5.85 (0.25-143)	6.46 (0.24-168)	0.73 (0.41-1.30)	0.73 (0.33-1.60)	0.72 (0.30-1.70)
LFIB	LF	**0.61 (0.41-0.91)**	0.66 (0.17-2.57)	0.64 (0.15-2.82)	-	-	-
LFIB	LFB	1.86 (0.41-8.19)	2.04 (0.08-54.2)	2.24 (0.07-63.0)	-	-	-
LFIB	LFBC	-	-	-	1.12 (0.59-2.12)	1.13 (0.46-2.75)	1.10 (0.41-3.01)
LFIB	LFP	2.81 (0.64-12.8)	3.04 (0.17-55.6)	3.22 (0.16-64.6)	0.99 (0.39-2.50)	0.99 (0.33-3.04)	0.94 (0.28-3.27)
LFIB	XB	-	-	-	0.69 (0.30-1.60)	0.68 (0.24-2.04)	0.69 (0.22-2.27)
LFIB	XI	4.90 (1.10-27.6)	5.05 (0.32-86.9)	4.99 (0.31-90.9)	-	-	-
LFIB	XIB	1.36 (0.86-2.14)	1.36 (0.25-7.43)	1.35 (0.24-7.67)	1.61 (0.84-3.12)	1.60 (0.71-3.60)	1.61 (0.67-3.92)
LFIB	XO	0.84 (0.57-1.25)	1.07 (0.24-4.91)	1.05 (0.21-5.25)	-	-	-
LFIB	XOB	1.80 (0.43-7.12)	2.06 (0.10-42.7)	2.24 (0.10-48.5)	0.37 (0.11-1.09)	0.36 (0.10-1.30)	0.36 (0.09-1.43)
LFIB	XOBC	2.97 (0.69-12.4)	3.41 (0.11-107)	3.70 (0.10-127)	0.59 (0.18-1.86)	0.58 (0.14-2.43)	0.59 (0.13-2.68)
LFIB	X	-	-	-	0.73 (0.27-1.97)	0.72 (0.20-2.64)	0.72 (0.18-3.06)
LFIB	O	0.61 (0.36-1.04)	0.68 (0.10-4.54)	0.67 (0.09-4.89)	-	-	-
LFIC	LFIP	1.18 (0.87-1.60)	1.36 (0.35-5.25)	1.43 (0.30-6.88)	1.42 (0.61-3.31)	1.40 (0.46-4.32)	1.20 (0.34-4.51)

T1	T2	Adverse events grade ≥3			Serious adverse events		
		Fixed effects	Random effects	Meta-regression	Fixed effects	Random effects	Meta-regression
LFIC	LFIBP	**0.44 (0.28-0.71)**	1.42 (0.32-7.54)	1.53 (0.24-11.3)	-	-	-
LFIC	LFO	**0.63 (0.46-0.87)**	0.78 (0.23-2.72)	0.78 (0.22-2.84)	0.51 (0.26-1.00)	0.51 (0.21-1.23)	0.51 (0.19-1.39)
LFIC	LFOB	0.94 (0.23-3.62)	1.06 (0.05-22.3)	1.19 (0.05-27.4)	0.63 (0.28-1.43)	0.65 (0.22-2.07)	0.68 (0.20-2.52)
LFIC	LFOC	1.04 (0.70-1.54)	1.15 (0.38-3.42)	1.14 (0.37-3.56)	0.91 (0.60-1.37)	0.91 (0.54-1.56)	0.91 (0.51-1.65)
LFIC	LFOP	1.93 (0.60-6.46)	2.21 (0.20-25.1)	2.40 (0.18-31.7)	0.83 (0.40-1.69)	0.86 (0.33-2.44)	0.86 (0.29-2.84)
LFIC	LFOIB	1.96 (0.46-8.07)	2.65 (0.10-74.4)	2.99 (0.09-94.6)	0.55 (0.22-1.39)	0.57 (0.16-2.08)	0.58 (0.15-2.48)
LFIC	LF	**0.26 (0.18-0.39)**	0.30 (0.07-1.26)	0.30 (0.07-1.29)	-	-	-
LFIC	LFB	0.80 (0.18-3.53)	0.93 (0.03-28.3)	1.04 (0.03-35.4)	-	-	-
LFIC	LFBC	-	-	-	0.84 (0.32-2.22)	0.87 (0.24-3.32)	0.90 (0.21-4.22)
LFIC	LFP	1.22 (0.28-5.52)	1.39 (0.07-29.6)	1.50 (0.06-37.0)	0.75 (0.26-2.16)	0.77 (0.21-3.10)	0.78 (0.18-3.69)
LFIC	XB	-	-	-	0.52 (0.16-1.72)	0.52 (0.11-2.57)	0.56 (0.11-3.27)
LFIC	XI	2.12 (0.46-12.1)	2.31 (0.11-50.4)	2.32 (0.11-55.6)	-	-	-
LFIC	XIB	**0.58 (0.35-0.99)**	0.62 (0.08-4.87)	0.63 (0.07-5.32)	1.22 (0.42-3.55)	1.23 (0.32-5.07)	1.32 (0.30-6.35)
LFIC	XO	**0.36 (0.25-0.53)**	0.49 (0.11-2.31)	0.49 (0.10-2.46)	-	-	-
LFIC	XOB	0.77 (0.19-3.06)	0.94 (0.04-22.5)	1.04 (0.04-27.8)	0.28 (0.07-1.03)	0.28 (0.06-1.37)	0.30 (0.05-1.77)
LFIC	XOBC	1.28 (0.30-5.33)	1.55 (0.04-55.1)	1.72 (0.04-69.9)	0.45 (0.11-1.75)	0.45 (0.08-2.45)	0.48 (0.08-3.28)
LFIC	X	-	-	-	0.55 (0.15-2.02)	0.55 (0.10-3.12)	0.58 (0.09-4.12)
LFIC	O	**0.26 (0.16-0.44)**	0.31 (0.05-2.14)	0.31 (0.04-2.29)	-	-	-
LFIP	LFIBP	**0.38 (0.23-0.61)**	1.04 (0.25-5.05)	1.07 (0.24-5.50)	-	-	-
LFIP	LFO	**0.54 (0.38-0.76)**	0.57 (0.16-2.00)	0.55 (0.12-2.43)	**0.36 (0.22-0.60)**	**0.37 (0.18-0.73)**	**0.43 (0.18-0.95)**
LFIP	LFOB	0.80 (0.20-3.00)	0.79 (0.05-11.6)	0.83 (0.05-12.6)	**0.45 (0.29-0.70)**	**0.46 (0.25-0.93)**	0.56 (0.26-1.30)
LFIP	LFOC	0.88 (0.56-1.39)	0.84 (0.18-3.92)	0.80 (0.14-4.67)	0.64 (0.31-1.35)	0.65 (0.24-1.73)	0.76 (0.23-2.30)
LFIP	LFOP	1.63 (0.53-5.30)	1.63 (0.22-12.2)	1.68 (0.22-13.1)	**0.59 (0.36-0.95)**	0.61 (0.33-1.20)	0.72 (0.34-1.56)
LFIP	LFOIB	1.66 (0.40-6.68)	1.95 (0.10-40.2)	2.10 (0.09-45.6)	**0.39 (0.21-0.73)**	**0.40 (0.17-0.99)**	0.49 (0.18-1.39)
LFIP	LF	**0.22 (0.15-0.33)**	**0.22 (0.05-0.90)**	0.21 (0.04-1.10)	-	-	-
LFIP	LFB	0.68 (0.16-2.91)	0.69 (0.03-15.4)	0.73 (0.03-17.4)	-	-	-
LFIP	LFBC	-	-	-	0.59 (0.30-1.18)	0.61 (0.24-1.67)	0.74 (0.25-2.43)
LFIP	LFP	1.03 (0.24-4.55)	1.02 (0.07-15.6)	1.05 (0.06-17.2)	0.53 (0.21-1.34)	0.54 (0.18-1.71)	0.65 (0.19-2.26)
LFIP	XB	-	-	-	**0.37 (0.15-0.90)**	0.37 (0.12-1.28)	0.47 (0.12-1.91)
LFIP	XI	1.80 (0.39-10.3)	1.69 (0.09-34.3)	1.63 (0.08-36.3)	-	-	-
LFIP	XIB	**0.50 (0.29-0.86)**	0.45 (0.06-3.21)	0.44 (0.06-3.36)	0.86 (0.42-1.76)	0.87 (0.35-2.34)	1.10 (0.36-3.43)
LFIP	XO	**0.31 (0.21-0.46)**	0.36 (0.08-1.69)	0.34 (0.06-2.02)	-	-	-
LFIP	XOB	0.66 (0.16-2.53)	0.69 (0.04-11.9)	0.73 (0.04-13.2)	**0.19 (0.06-0.59)**	**0.20 (0.05-0.76)**	0.25 (0.06-1.09)
LFIP	XOBC	1.09 (0.26-4.41)	1.14 (0.04-30.5)	1.21 (0.04-35.1)	0.31 (0.09-1.02)	0.32 (0.07-1.41)	0.40 (0.08-2.02)
LFIP	X	-	-	-	0.39 (0.14-1.09)	0.39 (0.10-1.63)	0.49 (0.10-2.47)
LFIP	O	**0.22 (0.13-0.38)**	0.23 (0.03-1.55)	0.22 (0.03-1.82)	-	-	-
LFIBP	LFO	1.42 (0.87-2.34)	0.55 (0.13-2.09)	0.51 (0.08-2.85)	-	-	-
LFIBP	LFOB	2.12 (0.50-8.67)	0.75 (0.03-15.4)	0.77 (0.03-16.3)	-	-	-
LFIBP	LFOC	**2.34 (1.31-4.19)**	0.80 (0.13-4.21)	0.75 (0.09-5.42)	-	-	-
LFIBP	LFOP	**4.33 (1.27-15.4)**	1.56 (0.12-17.8)	1.57 (0.11-19.2)	-	-	-
LFIBP	LFOIB	4.40 (0.98-19.3)	1.87 (0.06-51.4)	1.94 (0.06-56.9)	-	-	-
LFIBP	LF	0.60 (0.35-1.02)	0.21 (0.04-0.92)	0.19 (0.03-1.27)	-	-	-
LFIBP	LFB	1.80 (0.38-8.33)	0.66 (0.02-19.4)	0.68 (0.02-21.1)	-	-	-
LFIBP	LFP	2.72 (0.59-13.0)	0.97 (0.04-20.8)	0.98 (0.04-22.2)	-	-	-
LFIBP	XI	4.77 (0.99-28.5)	1.61 (0.07-37.1)	1.51 (0.06-39.7)	-	-	-

T1	T2	Adverse events grade ≥3			Serious adverse events		
		Fixed effects	Random effects	Meta-regression	Fixed effects	Random effects	Meta-regression
LFIBP	XIB	1.32 (0.68-2.58)	0.44 (0.04-3.75)	0.41 (0.04-4.09)	-	-	-
LFIBP	XO	0.82 (0.48-1.40)	0.34 (0.06-1.71)	0.32 (0.04-2.26)	-	-	-
LFIBP	XOB	1.74 (0.40-7.33)	0.66 (0.02-15.4)	0.68 (0.02-16.4)	-	-	-
LFIBP	XOBC	2.88 (0.64-12.7)	1.10 (0.03-37.3)	1.13 (0.02-41.2)	-	-	-
LFIBP	O	0.59 (0.31-1.13)	0.22 (0.03-1.53)	0.20 (0.02-1.97)	-	-	-
LFO	LFOB	1.49 (0.37-5.78)	1.37 (0.07-26.8)	1.52 (0.06-33.3)	1.24 (0.79-1.95)	1.27 (0.65-2.66)	1.32 (0.64-2.99)
LFO	LFOC	**1.65 (1.12-2.41)**	1.46 (0.40-5.23)	1.46 (0.38-5.46)	**1.77 (1.05-3.06)**	1.77 (0.87-3.59)	1.77 (0.80-3.90)
LFO	LFOP	3.05 (0.94-10.3)	2.83 (0.27-30.2)	3.06 (0.24-38.2)	**1.62 (1.29-2.04)**	**1.67 (1.09-2.88)**	**1.67 (1.01-3.16)**
LFO	LFOIB	3.10 (0.71-12.9)	3.40 (0.13-90.7)	3.80 (0.12-117)	1.07 (0.57-2.02)	1.10 (0.46-2.83)	1.15 (0.44-3.19)
LFO	LF	**0.42 (0.32-0.55)**	**0.38 (0.16-0.91)**	**0.38 (0.15-0.92)**	-	-	-
LFO	LFB	1.27 (0.28-5.62)	1.20 (0.04-34.2)	1.32 (0.04-42.8)	-	-	-
LFO	LFBC	-	-	-	1.64 (0.82-3.30)	1.69 (0.65-4.62)	1.75 (0.61-5.57)
LFO	LFP	1.92 (0.43-8.81)	1.78 (0.09-35.5)	1.91 (0.08-44.6)	1.46 (0.63-3.35)	1.49 (0.55-4.32)	1.52 (0.50-4.86)
LFO	XB	-	-	-	1.02 (0.39-2.72)	1.03 (0.30-3.83)	1.10 (0.27-4.79)
LFO	XI	3.35 (0.73-19.3)	2.96 (0.15-64.5)	2.96 (0.14-71.1)	-	-	-
LFO	XIB	0.93 (0.52-1.64)	0.79 (0.10-6.25)	0.80 (0.09-6.84)	**2.37 (1.05-5.43)**	2.39 (0.84-7.17)	2.59 (0.81-8.71)
LFO	XO	**0.57 (0.47-0.70)**	0.62 (0.24-1.63)	0.62 (0.23-1.68)	-	-	-
LFO	XOB	1.23 (0.29-4.88)	1.20 (0.05-27.0)	1.33 (0.05-33.8)	0.54 (0.16-1.68)	0.54 (0.14-2.08)	0.59 (0.14-2.44)
LFO	XOBC	2.02 (0.47-8.52)	2.00 (0.06-65.9)	2.19 (0.05-85.6)	0.87 (0.25-2.87)	0.87 (0.20-3.83)	0.95 (0.19-4.66)
LFO	X	-	-	-	1.07 (0.36-3.25)	1.07 (0.25-4.77)	1.16 (0.23-6.18)
LFO	O	**0.42 (0.27-0.64)**	0.40 (0.09-1.81)	0.39 (0.08-1.90)	-	-	-
LFOB	LFOC	1.11 (0.28-4.66)	1.07 (0.05-23.5)	0.96 (0.04-25.6)	1.43 (0.72-2.92)	1.40 (0.50-3.64)	1.34 (0.42-3.90)
LFOB	LFOP	**2.05 (1.02-4.32)**	2.07 (0.35-12.5)	2.03 (0.33-12.8)	1.31 (0.87-1.98)	1.33 (0.74-2.36)	1.26 (0.68-2.42)
LFOB	LFOIB	**2.07 (1.34-3.25)**	2.48 (0.65-9.88)	2.52 (0.64-10.4)	0.87 (0.55-1.35)	0.87 (0.49-1.53)	0.86 (0.46-1.60)
LFOB	LF	0.28 (0.07-1.16)	0.28 (0.01-5.81)	0.25 (0.01-6.43)	-	-	-
LFOB	LFB	0.85 (0.47-1.56)	0.87 (0.18-4.29)	0.87 (0.17-4.50)	-	-	-
LFOB	LFBC	-	-	-	1.33 (0.78-2.26)	1.34 (0.66-2.71)	1.32 (0.61-2.92)
LFOB	LFP	1.30 (0.40-4.13)	1.30 (0.10-17.2)	1.27 (0.09-18.1)	1.18 (0.48-2.90)	1.18 (0.40-3.49)	1.14 (0.35-3.66)
LFOB	XB	-	-	-	0.82 (0.33-2.07)	0.81 (0.24-2.70)	0.83 (0.22-3.21)
LFOB	XI	2.27 (0.30-20.1)	2.17 (0.04-119)	1.98 (0.03-121)	-	-	-
LFOB	XIB	0.62 (0.15-2.71)	0.58 (0.02-15.8)	0.53 (0.02-16.1)	1.92 (0.91-4.09)	1.89 (0.72-4.98)	1.95 (0.66-5.74)
LFOB	XO	0.39 (0.10-1.59)	0.45 (0.02-10.3)	0.41 (0.02-11.2)	-	-	-
LFOB	XOB	0.82 (0.63-1.08)	0.87 (0.33-2.33)	0.88 (0.32-2.41)	0.43 (0.14-1.23)	0.42 (0.13-1.35)	0.44 (0.13-1.44)
LFOB	XOBC	1.36 (0.88-2.13)	1.45 (0.21-9.95)	1.45 (0.20-10.6)	0.70 (0.22-2.10)	0.69 (0.18-2.51)	0.71 (0.18-2.76)
LFOB	X	-	-	-	0.86 (0.30-2.50)	0.85 (0.21-3.44)	0.87 (0.19-4.15)
LFOB	O	0.28 (0.07-1.20)	0.29 (0.01-7.90)	0.26 (0.01-8.63)	-	-	-
LFOC	LFOP	1.85 (0.55-6.48)	1.94 (0.16-24.8)	2.10 (0.14-31.0)	0.91 (0.51-1.62)	0.95 (0.42-2.30)	0.94 (0.38-2.62)
LFOC	LFOIB	1.88 (0.42-8.09)	2.32 (0.08-71.1)	2.60 (0.08-91.4)	0.60 (0.26-1.38)	0.63 (0.20-2.01)	0.64 (0.18-2.42)
LFOC	LF	**0.25 (0.16-0.40)**	0.26 (0.06-1.18)	0.26 (0.06-1.22)	-	-	-
LFOC	LFB	0.77 (0.16-3.53)	0.82 (0.03-26.9)	0.91 (0.02-33.7)	-	-	-
LFOC	LFBC	-	-	-	0.93 (0.38-2.22)	0.96 (0.29-3.29)	0.99 (0.27-4.10)
LFOC	LFP	1.17 (0.26-5.53)	1.21 (0.05-28.4)	1.31 (0.05-35.5)	0.82 (0.30-2.20)	0.84 (0.25-3.15)	0.85 (0.22-3.60)
LFOC	XB	-	-	-	0.58 (0.19-1.75)	0.58 (0.14-2.57)	0.62 (0.13-3.31)
LFOC	XI	2.04 (0.43-12.1)	2.01 (0.09-49.0)	2.04 (0.08-53.8)	-	-	-
LFOC	XIB	0.56 (0.30-1.06)	0.54 (0.06-5.02)	0.55 (0.06-5.44)	1.34 (0.50-3.57)	1.35 (0.38-4.99)	1.46 (0.37-6.18)

T1	T2	Adverse events grade ≥3			Serious adverse events		
		Fixed effects	Random effects	Meta-regression	Fixed effects	Random effects	Meta-regression
LFOC	XO	**0.35 (0.23-0.54)**	0.43 (0.09-2.11)	0.43 (0.08-2.24)	-	-	-
LFOC	XOB	0.74 (0.17-3.07)	0.82 (0.03-21.6)	0.91 (0.03-27.0)	0.30 (0.08-1.07)	0.30 (0.07-1.36)	0.33 (0.06-1.73)
LFOC	XOBC	1.23 (0.28-5.33)	1.36 (0.03-51.7)	1.51 (0.03-66.0)	0.49 (0.13-1.82)	0.49 (0.10-2.48)	0.53 (0.09-3.26)
LFOC	X	-	-	-	0.60 (0.18-2.07)	0.61 (0.12-3.11)	0.65 (0.11-4.12)
LFOC	O	**0.25 (0.14-0.45)**	0.27 (0.04-1.96)	0.27 (0.04-2.09)	-	-	-
LFOP	LFOIB	1.01 (0.42-2.30)	1.20 (0.13-11.5)	1.24 (0.13-12.9)	0.66 (0.36-1.21)	0.65 (0.29-1.50)	0.68 (0.28-1.64)
LFOP	LF	**0.14 (0.04-0.45)**	0.13 (0.01-1.54)	0.12 (0.01-1.73)	-	-	-
LFOP	LFB	0.41 (0.16-1.05)	0.42 (0.04-4.50)	0.43 (0.04-4.99)	-	-	-
LFOP	LFBC	-	-	-	1.01 (0.52-1.98)	1.01 (0.40-2.46)	1.05 (0.38-2.87)
LFOP	LFP	0.63 (0.25-1.54)	0.63 (0.10-3.97)	0.62 (0.09-4.20)	0.90 (0.40-2.00)	0.89 (0.36-2.23)	0.90 (0.33-2.42)
LFOP	XB	-	-	-	0.63 (0.24-1.66)	0.61 (0.18-2.12)	0.66 (0.17-2.63)
LFOP	XI	1.11 (0.16-8.78)	1.04 (0.03-38.2)	0.97 (0.03-38.7)	-	-	-
LFOP	XIB	0.30 (0.08-1.08)	0.28 (0.02-4.48)	0.26 (0.01-4.67)	1.46 (0.66-3.30)	1.43 (0.51-3.99)	1.54 (0.49-4.80)
LFOP	XO	**0.19 (0.05-0.62)**	0.22 (0.02-2.78)	0.20 (0.01-3.06)	-	-	-
LFOP	XOB	**0.40 (0.18-0.85)**	0.42 (0.06-3.19)	0.43 (0.05-3.50)	0.33 (0.10-1.02)	0.32 (0.08-1.15)	0.35 (0.09-1.32)
LFOP	XOBC	0.66 (0.28-1.52)	0.70 (0.05-9.48)	0.71 (0.05-10.7)	0.54 (0.16-1.75)	0.52 (0.12-2.12)	0.56 (0.12-2.50)
LFOP	X	-	-	-	0.66 (0.22-1.98)	0.64 (0.15-2.69)	0.69 (0.14-3.43)
LFOP	O	**0.14 (0.04-0.48)**	0.14 (0.01-2.24)	0.13 (0.01-2.53)	-	-	-
LFOIB	LF	**0.14 (0.03-0.60)**	0.11 (0.00-3.07)	0.10 (0.00-3.33)	-	-	-
LFOIB	LFB	**0.41 (0.19-0.87)**	0.35 (0.04-2.78)	0.35 (0.04-2.90)	-	-	-
LFOIB	LFBC	-	-	-	1.54 (0.77-3.07)	1.54 (0.62-3.84)	1.53 (0.57-4.20)
LFOIB	LFP	0.62 (0.18-2.18)	0.52 (0.03-9.46)	0.50 (0.02-9.95)	1.36 (0.50-3.71)	1.36 (0.39-4.71)	1.33 (0.35-5.00)
LFOIB	XB	-	-	-	0.95 (0.34-2.63)	0.94 (0.25-3.55)	0.96 (0.22-4.28)
LFOIB	XI	1.10 (0.14-10.1)	0.87 (0.01-61.0)	0.79 (0.01-60.0)	-	-	-
LFOIB	XIB	0.30 (0.07-1.38)	0.23 (0.01-8.33)	0.21 (0.01-8.39)	2.22 (0.93-5.32)	2.19 (0.71-6.73)	2.24 (0.65-7.93)
LFOIB	XO	**0.19 (0.04-0.81)**	0.18 (0.01-5.53)	0.16 (0.00-5.77)	-	-	-
LFOIB	XOB	**0.40 (0.23-0.67)**	0.35 (0.06-1.82)	0.35 (0.06-1.90)	0.50 (0.15-1.56)	0.49 (0.13-1.78)	0.51 (0.13-1.96)
LFOIB	XOBC	0.66 (0.35-1.23)	0.58 (0.05-5.92)	0.58 (0.05-6.36)	0.81 (0.24-2.66)	0.79 (0.18-3.28)	0.83 (0.18-3.66)
LFOIB	X	-	-	-	1.00 (0.32-3.16)	0.97 (0.22-4.42)	1.01 (0.19-5.52)
LFOIB	O	**0.14 (0.03-0.62)**	0.12 (0.00-4.17)	0.10 (0.00-4.42)	-	-	-
LF	LFB	3.03 (0.66-13.7)	3.12 (0.10-96.1)	3.47 (0.09-124)	-	-	-
LF	LFP	**4.59 (1.03-21.5)**	4.65 (0.22-99.8)	5.00 (0.19-131)	-	-	-
LF	XI	**8.04 (1.70-46.8)**	7.72 (0.36-178)	7.75 (0.33-203)	-	-	-
LF	XIB	**2.21 (1.21-4.07)**	2.06 (0.24-18.2)	2.11 (0.22-20.3)	-	-	-
LF	XO	1.37 (0.98-1.92)	1.62 (0.51-5.36)	1.63 (0.49-5.62)	-	-	-
LF	XOB	2.93 (0.69-11.9)	3.15 (0.13-76.0)	3.47 (0.12-98.0)	-	-	-
LF	XOBC	4.84 (1.11-20.6)	5.19 (0.14-187)	5.74 (0.13-244)	-	-	-
LF	O	1.00 (0.65-1.52)	1.03 (0.23-4.77)	1.03 (0.22-5.03)	-	-	-
LFB	LFP	1.53 (0.41-5.63)	1.49 (0.07-30.8)	1.45 (0.06-33.4)	-	-	-
LFB	XI	2.69 (0.32-25.6)	2.48 (0.03-184)	2.27 (0.03-188)	-	-	-
LFB	XIB	0.73 (0.16-3.54)	0.66 (0.02-26.0)	0.61 (0.01-26.6)	-	-	-
LFB	XO	0.45 (0.10-2.08)	0.52 (0.02-17.1)	0.47 (0.01-18.7)	-	-	-
LFB	XOB	0.97 (0.53-1.76)	1.00 (0.20-4.88)	1.00 (0.20-5.15)	-	-	-
LFB	XOBC	1.60 (0.79-3.22)	1.65 (0.17-16.5)	1.66 (0.16-17.7)	-	-	-
LFB	O	0.33 (0.07-1.58)	0.33 (0.01-13.2)	0.30 (0.01-14.2)	-	-	-

T1	T2	Adverse events grade ≥3			Serious adverse events		
		Fixed effects	Random effects	Meta-regression	Fixed effects	Random effects	Meta-regression
LFBC	LFP	-	-	-	0.88 (0.31-2.52)	0.88 (0.25-3.22)	0.86 (0.21-3.47)
LFBC	XB	-	-	-	0.62 (0.22-1.78)	0.61 (0.15-2.46)	0.63 (0.14-2.96)
LFBC	XIB	-	-	-	1.44 (0.58-3.61)	1.43 (0.42-4.68)	1.46 (0.39-5.55)
LFBC	XOB	-	-	-	0.33 (0.10-1.06)	0.32 (0.08-1.24)	0.33 (0.08-1.36)
LFBC	XOBC	-	-	-	0.53 (0.15-1.80)	0.51 (0.12-2.24)	0.54 (0.11-2.56)
LFBC	X	-	-	-	0.65 (0.20-2.12)	0.64 (0.13-3.05)	0.66 (0.12-3.73)
LFP	XB	-	-	-	0.70 (0.20-2.45)	0.69 (0.15-3.24)	0.73 (0.13-4.03)
LFP	XI	1.77 (0.21-16.9)	1.67 (0.03-94.7)	1.56 (0.03-99.7)	-	-	-
LFP	XIB	0.48 (0.10-2.28)	0.45 (0.02-12.7)	0.42 (0.01-13.3)	1.63 (0.53-5.09)	1.61 (0.41-6.46)	1.71 (0.36-7.71)
LFP	XO	0.30 (0.06-1.33)	0.35 (0.02-8.16)	0.33 (0.01-8.81)	-	-	-
LFP	XOB	0.63 (0.19-2.09)	0.67 (0.04-10.6)	0.69 (0.04-11.8)	0.37 (0.09-1.47)	0.36 (0.07-1.73)	0.39 (0.07-2.03)
LFP	XOBC	1.05 (0.30-3.63)	1.11 (0.04-27.5)	1.14 (0.04-31.7)	0.60 (0.14-2.50)	0.58 (0.10-3.14)	0.63 (0.10-3.78)
LFP	X	-	-	-	0.73 (0.19-2.85)	0.72 (0.13-3.91)	0.76 (0.11-5.05)
LFP	O	0.22 (0.04-1.01)	0.22 (0.01-6.22)	0.21 (0.01-6.97)	-	-	-
XB	XIB	-	-	-	**2.33 (1.37-3.95)**	**2.34 (1.14-4.73)**	**2.33 (1.07-5.07)**
XB	XOB	-	-	-	0.53 (0.13-2.07)	0.52 (0.10-2.83)	0.53 (0.09-3.18)
XB	XOBC	-	-	-	0.85 (0.20-3.51)	0.84 (0.14-5.06)	0.85 (0.12-5.75)
XB	X	-	-	-	1.05 (0.62-1.77)	1.04 (0.52-2.12)	1.04 (0.48-2.29)
XI	XIB	0.28 (0.05-1.13)	0.27 (0.03-2.37)	0.27 (0.03-2.43)	-	-	-
XI	XO	**0.17 (0.03-0.81)**	0.21 (0.01-4.95)	0.21 (0.01-5.23)	-	-	-
XI	XOB	0.36 (0.04-2.82)	0.40 (0.01-24.2)	0.44 (0.01-29.6)	-	-	-
XI	XOBC	0.60 (0.06-4.84)	0.67 (0.01-55.0)	0.73 (0.01-67.7)	-	-	-
XI	O	**0.12 (0.02-0.61)**	0.13 (0.00-3.81)	0.13 (0.00-4.13)	-	-	-
XIB	XO	0.62 (0.34-1.14)	0.79 (0.08-7.65)	0.78 (0.07-8.15)	-	-	-
XIB	XOB	1.33 (0.30-5.60)	1.51 (0.05-48.7)	1.64 (0.05-58.3)	**0.23 (0.06-0.80)**	0.22 (0.05-1.03)	0.23 (0.04-1.16)
XIB	XOBC	2.20 (0.48-9.73)	2.51 (0.05-117)	2.72 (0.05-140)	0.36 (0.09-1.36)	0.36 (0.07-1.85)	0.36 (0.06-2.11)
XIB	X	-	-	-	**0.45 (0.21-0.95)**	0.44 (0.17-1.22)	0.45 (0.15-1.36)
XIB	O	**0.45 (0.22-0.90)**	0.50 (0.04-6.37)	0.49 (0.04-6.89)	-	-	-
XO	XOB	2.13 (0.51-8.60)	1.93 (0.07-48.6)	2.14 (0.07-62.4)	-	-	-
XO	XOBC	3.53 (0.81-15.1)	3.23 (0.08-119)	3.52 (0.08-155)	-	-	-
XO	O	0.73 (0.46-1.16)	0.63 (0.11-3.67)	0.63 (0.10-3.86)	-	-	-
XOB	XOBC	**1.66 (1.17-2.37)**	1.65 (0.31-8.74)	1.66 (0.30-9.22)	**1.62 (1.15-2.30)**	1.62 (0.89-2.94)	1.62 (0.82-3.24)
XOB	X	-	-	-	2.00 (0.46-8.93)	2.00 (0.31-12.7)	1.96 (0.28-14.6)
XOB	O	0.34 (0.08-1.50)	0.33 (0.01-10.5)	0.30 (0.01-11.2)	-	-	-
XOBC	X	-	-	-	1.24 (0.27-5.74)	1.25 (0.18-8.55)	1.21 (0.16-9.94)
XOBC	O	**0.21 (0.05-0.94)**	0.20 (0.00-9.35)	0.18 (0.00-10.1)	-	-	-

X, capecitabine; B, bevacizumab; C, cetuximab; F, 5-fluorouracil; I, irinotecan; L, leucovorin; O, oxaliplatin; P, panitumumab. Bold fonts indicate significant difference.

Supplementary Table S7. Node-splitting model for testing of consistency assumption.

ORR		DCR		OS		PFS		AEs grade ≥3		SAEs	
**Comparison**	**p-value**	**Comparison**	**p-value**	**Comparison**	**p-value**	**Comparison**	**p-value**	**Comparison**	**p-value**	**Comparison**	**p-value**
I vs CI	0.80	I vs CI	0.76	LFO vs IO	0.65	LFO vs IO	0.87	LFIB vs LFI	0.97	LFIB vs LFI	0.97
IP vs CI	0.80	IP vs CI	0.76	LFIB vs LFI	0.07	LFIB vs LFI	0.10	LFIP vs LFI	0.41	LFIP vs LFI	0.39
IO vs I	0.07	IO vs I	0.05	LFIC vs LFI	0.06	LFIC vs LFI	0.17	LFO vs LFI	0.14	LFO vs LFI	0.13
IP vs I	0.80	IP vs I	0.75	LFIP vs LFI	0.84	LFIP vs LFI	0.31	LFIP vs LFIB	0.91	LFIP vs LFIB	0.91
LFI vs I	0.06	LFI vs I	0.05	LFO vs LFI	0.56	LFO vs LFI	0.49	LFOB vs LFIB	0.88	LFOB vs LFIB	0.85
LFI vs IO	0.07	LFI vs IO	0.05	LFIC vs LFIB	0.67	LFIC vs LFIB	0.96	LFOP vs LFIP	0.30	LFOP vs LFIP	0.24
LFO vs IO	0.37	LFO vs IO	0.84	LFIP vs LFIB	0.76	LFIP vs LFIB	0.36	LFOP vs LFO	0.18	LFOP vs LFO	0.13
LFI vs LF	0.47	LFI vs LF	0.26	LFOB vs LBIB	0.11	LFOB vs LFIB	0.23	LFOP vs LFOB	0.86	LFOP vs LFOB	0.86
LFO vs LF	0.53	LFO vs LF	0.27	**LFOIB vs LFIB**	**0.01**	LFOIB vs LFIB	0.09	LFIB vs LFI	0.97		
XO vs LF	0.24	XO vs LF	0.44	XIB vs LFIB	0.39	XIB vs LFIB	0.43	LFIP vs LFI	0.41		
LFIB vs LFI	0.97	LFIB vs LFI	0.02	LFOC vs LFIC	0.06	LFOC vs LFIC	0.17	LFO vs LFI	0.14		
LFIC vs LFI	0.06	LFIC vs LFI	0.29	LFOP vs LFIP	0.55	LFOP vs LFIP	0.77				
LFIP vs LFI	0.80	LFIP vs LFI	0.62	LFOC vs LFO	0.07	LFOC vs LFO	0.30				
LFO vs LFI	0.29	LFO vs LFI	0.62	LFOP vs LFO	0.34	LFOP vs LFO	0.82				
LFOI vs LFI	0.33	LFOI vs LFI	0.82	**LFOIB vs LFOB**	**0.01**	LFOC vs LFOB	0.79				
LFIC vs LFIB	0.98	LFIC vs LFIB	0.18	LFOP vs LFOB	0.10	LFOIB vs LFOB	0.09				
**LFIP vs LFIB**	**0.01**	LFIP vs LFIB	0.61	XOB vs LFOB	0.40	LFOP vs LFOB	0.60				
LFOB vs LFIB	0.33	LFOB vs LFIB	0.32	XOB vs XIB	0.40	XOB vs LFOB	0.44				
LFOIB vs LFIB	0.89	XIB vs LFIB	0.61			XOB vs XIB	0.44				
XIB vs LFIB	0.70	LFOC vs LFIC	0.83								
LFOC vs LFIC	0.24	LFOC vs LFO	0.84								
LFOIC vs LFIC	0.21	**LFOP vs LFO**	**0.04**								
**LFOP vs LFIP**	**0.01**	XO vs LFO	0.95								
LFOB vs LFO	0.23	LFOIB vs LFOB	0.82								
LFOC vs LFO	0.31	LFOP vs LFOB	0.05								
LFOP vs LFO	0.08	XOB vs LFOB	0.57								

ORR		DCR		OS		PFS		AEs grade ≥3		SAEs	
**Comparison**	**p-value**	**Comparison**	**p-value**	**Comparison**	**p-value**	**Comparison**	**p-value**	**Comparison**	**p-value**	**Comparison**	**p-value**
XO vs LFO	0.37	LFOIB vs LFOI	0.82								
LFOC vs LFOB	0.88	XB vs X	0.32								
LFOIB vs LFOB	0.92	XO vs X	0.31								
LFOP vs LFOB	0.88	XIB vs XB	0.33								
XOB vs LFOB	0.95	XOB vs XIB	0.23								
LFOIB vs LFOI	0.98	**XOC vs XO**	**0.01**								
LFOIC vs LFOI	0.21	**XOBC vs XOB**	**0.01**								
XB vs X	0.35	**XOC vs XOBC**	**0.004**								
XO vs X	0.36										
XIB vs XB	0.36										
XOB vs XIB	0.99										
XOC vs XO	0.80										
XOBC vs XOB	0.82										
XOC vs XOBC	0.82										

ORR, overall response rate; DCR, disease control rate; OS, overall survival; PFS, progression-free survival; AEs, adverse events; SAEs, serious adverse events; X, capecitabine; B, bevacizumab; C, cetuximab; F, 5-fluorouracil; I, irinotecan; L, leucovorin; O, oxaliplatin; P, panitumumab. Bold fonts indicate significant difference.

Supplementary Table S8. Global I^2 for between-trial heterogeneity.

**Outcome**	**ORR**	**DCR**	**OS**	**PFS**	**AEs grade ≥3**	**SAEs**
Pairwise I^2	39.02	31.85	30.36	69.24	84.41	0
Consistent I^2	37.70	49.46	53.18	70.08	81.54	0

ORR, overall response rate; DCR, disease control rate; OS, overall survival; PFS, progression-free survival; AEs, adverse events; SAEs, serious adverse events.

Supplementary Table S9. Treatment ranking probability for primary and secondary treatments of colorectal cancer.

Treatment	ORR		DCR		OS		PFS		AEs grade ≥3		SAEs	
	1st line	2nd line	1st line	2nd line	1st line	2nd line	1st line	2nd line	1st line	2nd line	1st line	2nd line
LFI	0.00	0.00	0.00	0.00	0.00	0.00	0.00	0.00	0.14	0.80	0.02	0.08
LFIB	0.00	0.00	0.74	9.30	0.00	0.00	0.56	2.41	5.23	9.48	0.09	0.37
LFIC	0.00	0.00	0.00	0.00	0.26	1.22	0.54	1.82	0.34	0.80	0.92	1.26
LFIP	0.21	1.51	0.00	0.03	0.00	0.01	0.05	0.19	0.02	0.05	0.01	0.02
**LFIBP**	0.02	0.16	0.24	2.79	4.40	8.94	18.45	**19.08**	0.16	0.38		
LFO	0.00	0.00	0.00	0.00	0.00	0.02	0.06	0.51	0.02	0.21	6.23	10.48
LFOB	0.00	0.00	0.26	3.21	0.00	0.01	0.04	0.26	1.73	3.91	0.11	0.80
LFOC	0.06	0.53	0.05	0.60	2.13	6.68	3.69	10.36	0.43	0.89	0.44	1.20
LFOP	0.00	0.01	0.06	0.73	0.06	0.54	0.55	2.25	0.06	0.23	0.01	0.14
**LFOI**	0.00	0.00	0.00	0.40	0.00	0.01	0.00	0.00				
**LFOIB**	0.05	0.59	7.19	**47.04**	0.16	0.40	2.89	6.98	0.60	0.83	4.00	7.48
LFOIC	7.50	15.65										
**LFOIP**	**43.00**	23.21	**85.62**	7.20			0.19	0.27				
**LF**	0.00	0.00	0.00	0.00	0.05	0.23	0.69	1.73	21.03	**25.83**		
LFB	0.00	0.01	0.08	0.50					9.62	6.26		
LFC	0.00	0.00			0.97	1.84	1.5	2.12				
LFBC	0.00	0.00	21.12	6.16							0.61	1.13
LFP	0.08	0.44			28.00	15.77	6.23	7.56	5.10	3.36	3.23	4.19
CB	0.10	0.31										
CI	0.04	0.33	0.00	0.06								
CIB	1.53	4.26	0.19	1.24								
IO	0.00	0.00	0.00	0.00	0.02	0.07	0.01	0.05				
IOB	0.03	0.12	0.03	0.17	0.05	0.13	3.02	4.46				
IP	0.07	0.24	0.00	0.02								
FI	0.00	0.00	0.01	0.10								
XB	0.00	0.00	0.02	0.19	**55.80**	21.74	**39.05**	11.95			8.34	13.48
XC	25.89	9.62	1.17	4.06								
XI	0.23	0.68	1.76	7.41					2.84	2.64		
XIB	0.00	0.00	0.03	0.37	0.05	0.27	0.83	2.62	9.22	7.70	0.02	0.06
XIC	6.49	15.97	0.11	0.65								
XO	0.00	0.00	0.00	0.05	0.01	0.09	0.18	0.94	5.33	9.54		
**XOB**	0.00	0.00	0.01	0.11	0.06	0.38	0.38	2.48	4.68	7.54	**61.97**	11.85
**XOC**	14.70	**26.29**	0.98	5.59								
**XOBC**	0.00	0.03	0.31	1.97	3.13	5.98	21.06	18.63	4.64	3.80	2.72	**35.82**
X	0.00	0.00	0.00	0.00	4.83	**35.67**	0.03	3.34			11.31	11.66

Treatment	ORR		DCR		OS		PFS		AEs grade ≥3		SAEs	
	**1st line**	**2nd line**	**1st line**	**2nd line**	**1st line**	**2nd line**	**1st line**	**2nd line**	**1st line**	**2nd line**	**1st line**	**2nd line**
C	0.00	0.00	0.00	0.00								
I	0.00	0.00	0.00	0.00								
**O**	0.00	0.00	0.00	0.00					**28.84**	15.74		
P	0.01	0.04	0.01	0.06								

ORR, overall response rate; DCR, disease control rate; OS, overall survival; PFS, progression-free survival; AEs, adverse events; SAEs, serious adverse events; X, capecitabine; B, bevacizumab; C, cetuximab; F, 5-fluorouracil; I, irinotecan; L, leucovorin; O, oxaliplatin; P, panitumumab. Bold fonts indicate highest probabilities.

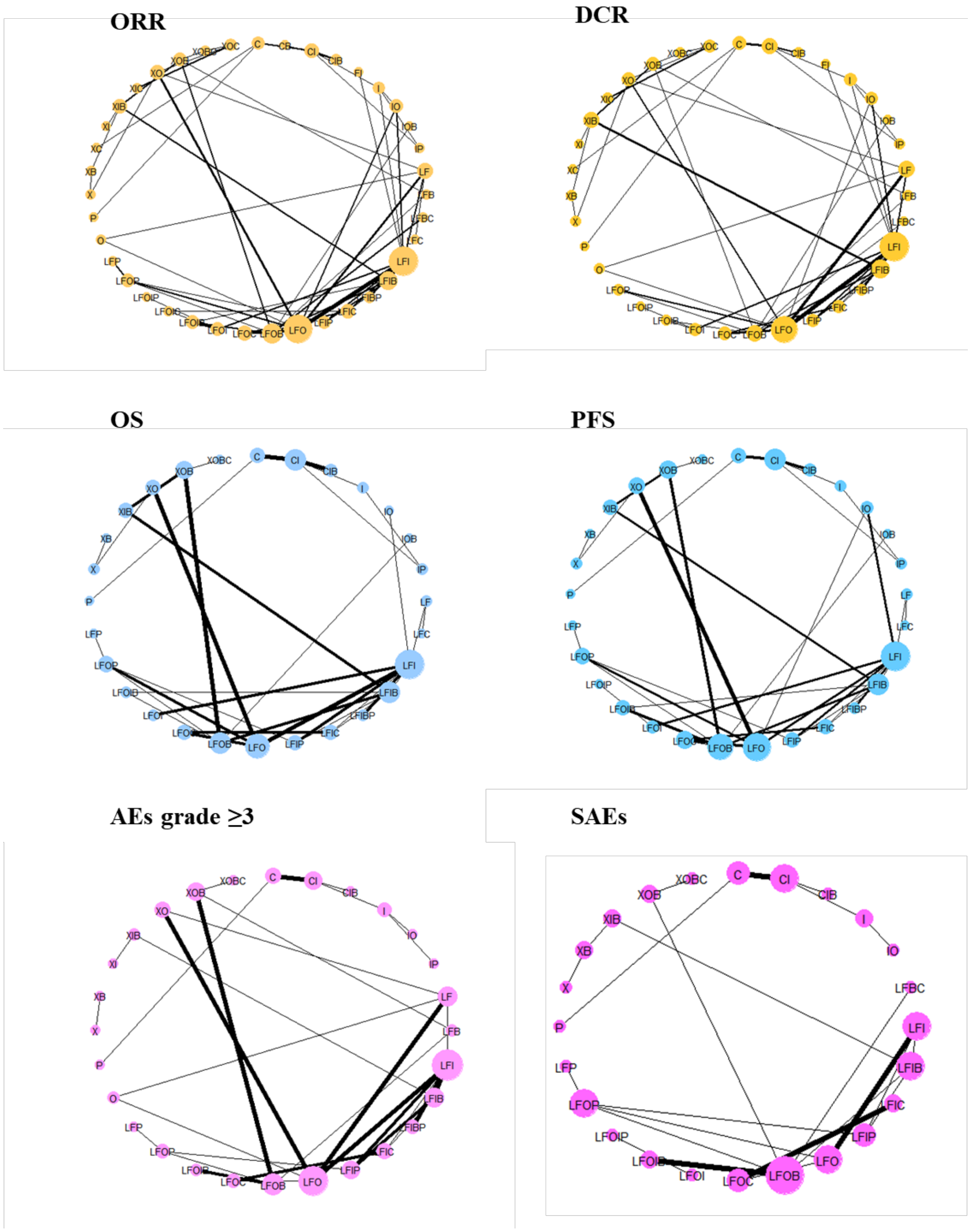

Supplementary Figure S1. Network geometry of all possible treatments.

ORR, overall response rate; DCR, disease control rate; OS, overall survival; PFS, progression-free survival; AEs, adverse events; SAEs, serious adverse events; X, capecitabine; B, bevacizumab; C, cetuximab; F, 5-fluorouracil; I, irinotecan; L, leucovorin; O, oxaliplatin; P, panitumumab. The network plots are based on treatments performing a connected network according to different types of outcomes. The size of node relates the number of participants for each treatment, and the thickness of line illustrates the number of studies for each comparison.
